# Structural impact of thioamide incorporation into a β-hairpin†

**DOI:** 10.1039/d1cb00229e

**Published:** 2022-04-05

**Authors:** Kristen E. Fiore, Martijn J. Patist, Sam Giannakoulias, Cheng-Hsin Huang, Hitesh Verma, Bhavesh Khatri, Richard P. Cheng, Jayanta Chatterjee, E. James Petersson

**Affiliations:** Department of Chemistry, University of Pennsylvania 231 S. 34th Street Philadelphia 19104 USA ejpetersson@sas.upenn.edu; Department of Chemistry, National Taiwan University No. 1, Sec. 4, Roosevelt Road Taipei 10617 Taiwan; Molecular Biophysics Unit, Indian Institute of Science Bangalore 560012 India

## Abstract

The thioamide is a naturally-occurring single atom substitution of the canonical amide bond. The exchange of oxygen to sulfur alters the amide's physical and chemical characteristics, thereby expanding its functionality. Incorporation of thioamides in prevalent secondary structures has demonstrated that they can either have stabilizing, destabilizing, or neutral effects. We performed a systematic investigation of the structural impact of thioamide incorporation in a β-hairpin scaffold with nuclear magnetic resonance (NMR). Thioamides as hydrogen bond donors did not increase the foldedness of the more stable “YKL” variant of this scaffold. In the less stable “HPT” variant of the scaffold, the thioamide could be stabilizing as a hydrogen bond donor and destabilizing as a hydrogen bond acceptor, but the extent of the perturbation depended upon the position of incorporation. To better understand these effects we performed structural modelling of the macrocyclic folded HPT variants. Finally, we compare the thioamide effects that we observe to previous studies of both side-chain and backbone perturbations to this β-hairpin scaffold to provide context for our observations.

## Abstract 2

NMR studies of macrocyclic β-hairpin model systems demonstrate that thioamides can be tolerated at both hydrogen bond donor and hydrogen bond acceptor positions.

## Graphical abstract

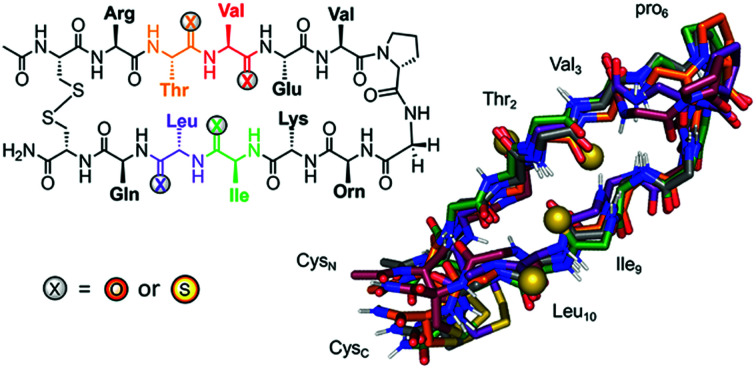

## Introduction

The thioamide is an intriguing isostere of the canonical amide bond. Although it differs by only a single atom, the thioamide has unique chemical and physical properties that have been employed by chemists and biophysicists. For example, the thioamide has a lower oxidation potential.^1^ Consequently, thioamides can quench fluorescence in a distance dependent manner: Förster resonance energy transfer (FRET)-based quenching of UV fluorophores and photoinduced electron transfer (PeT)-based quenching of visible fluorophores.^2^ Therefore, fluorophore/thioamide pairs can be utilized as minimal biophysical probes to study protein folding or dynamics. This has been done to monitor peptide–protein binding,^3–5^ to monitor protease activity in real time,^6^ and to monitor protein conformational changes during refolding,^3^ unfolding,^7,8^ or misfolding.^5^

Another unique property of the thioamide is that is has a red-shifted π-to-π* absorption,^9^ giving it a unique circular dichroism (CD) signature.^10^ This red-shifted absorption also lowers the excitation energy required for photoisomerization.^11^ Therefore, upon irradiation the thioamide can selectivity photoisomerize from *trans*-to-*cis*, enabling its use as a photoswitch in peptides.^12–15^

Nature installs thioamides in ribosomally synthesized and post-translationally modified peptides (RiPPs),^16^ as well as in at least two proteins: methyl coenzyme M reductase (MCR)^17,18^ and the uL16 protein of the *E. coli* 70S ribosome.^19^ Although the method of installation is well-studied, the effect of thioamidation on protein function is relatively unknown.

**Fig. 1:**
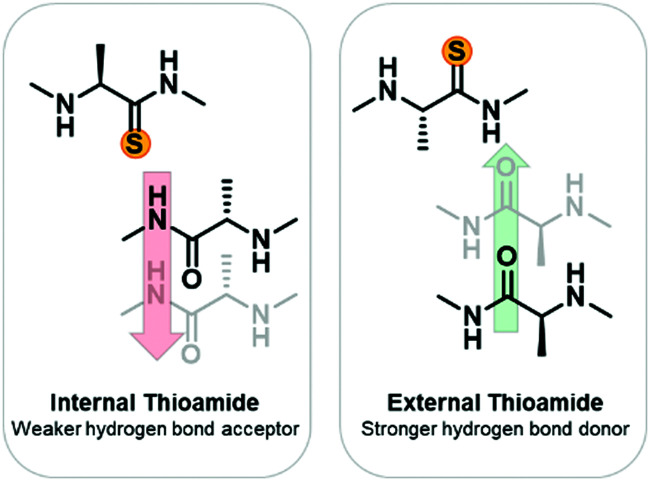
Expected thioamide effects on hydrogen bonding. Based on previous small molecule studies, it is expected that an internal thioamide will be disruptive to β-hairpin structure due to the thioamide being a weaker hydrogen bond acceptor and having a larger van der Waals radius. An external thioamide will be stabilizing since it is a stronger hydrogen bond donor than an amide.

To develop a systematic understanding of how the thioamide can affect biological activity, as well as to promote its utility as a biophysical probe, a more fundamental understanding of the structural impact of thioamide incorporation is needed. Previously, small molecule studies have suggested that the lower electronegativity of the thioamide sulfur results in it being a weaker hydrogen bond acceptor.^20–22^ Conversely, the lower N–H p*K*_a_ (12 *versus* 14)^23^ should result in the thioamide being a stronger hydrogen bond donor (Fig. 1).^24,25^ Additionally the larger van der Waals radius of sulfur^26^ results in a longer C=S double bond^27,28^ which could be perturbative depending upon the environment. To determine the impact on protein thermodynamic stability, we incorporated thioamides into native proteins of different secondary structures: calmodulin (α-helical), the B1 domain of protein G (GB1, β-sheet), and collagen (PPII triple-helix).^29^ In some cases, reasonable explanations could be made as to why incorporation at some positions was more destabilizing than others based on the existing structures of the native proteins. However, many seemingly similar thioamide substitutions resulted in very different effects on protein stability. We were particularly intrigued by the effects on GB1, where substitution in the same β-strand had destabilizing effects differing by 2 kcal mol^−1^. The same variation was observed in another β-sheet structure, the Pin1 WW domain (three β-strands), where the thermostability (Δ*T*_M(thio–oxo)_) varied from −0.9 to 14.8 °C depending upon the microenvironment of the position.^30^ To further investigate the effects of thioamides in β-sheets, we turned to model peptide systems, which have proven to be valuable for rigorous investigation of protein modifications.

Thioamides have been previously investigated in α-helical^31,32^ and poly-proline II (PPII) helical model systems.^33^ The structural impacts were scaffold and position specific. In contrast, there has been very limited study of thioamides in β-sheet model systems with only four examples to our knowledge. In two of the studies, thioamide substitution occurred in the turn, and therefore is not informative on how the chemical and physical properties of the thioamide affect cross-strand β-sheet interactions.^34,35^ In a well-studied β-hairpin, the tryptophan zipper (Trpzip), thioamides were incorporated as hydrogen bond acceptors and the thermodynamic stability measured using CD spectroscopy.^36^ Overall, the thioamides within the strands were destabilizing by about 1 kcal mol^−1^, with the terminal position being the least perturbative. Thioamides were also incorporated into a TrpZip with an azobenzene derivative at the β-turn.^37^ This allowed for control of the folding state of the peptide by photo-initiated *cis*/*trans* isomerization (*cis* = folded, *trans* = unfolded). Folding was observed with time-resolved IR spectroscopy and CD. For these TrpZips, two thioamides were incorporated to observe site-specific coupling spectroscopically. Thioamides on the same strand serving as hydrogen bond donors were minimally perturbative with unfolding rates similar to the all-amide reference. Thioamides on opposite strands serving as hydrogen bond donors stabilized the β-hairpin relative to the reference. Thioamides on opposite strands as hydrogen bond acceptors strongly destabilized the β-hairpin.

In these studies, the impact of thioamides on β-sheets are largely interpreted in terms of differing hydrogen bonding properties. However, such a simple interpretation is inconsistent with our observations in GB1, where local structure significantly altered the impact of thioamide substitution. Moreover, although these two studies include elegant kinetic and thermodynamic studies, they lack any direct structural information. Although TrpZips have been well-characterized, we were interested in studying a less-folded β-hairpin scaffold that might be more sensitive to effects of the thioamide and one for which an extensive body of literature on other non-covalent interactions is available for comparison. Therefore, we have designed a systematic investigation of thioamide incorporation using a model β-hairpin system that meets these requirements and performed structural analysis with NMR. This experimental data is supplemented with structural modelling of the macrocyclic folded variants.

## Results and discussion

### Scaffold design

**Fig. 2:**
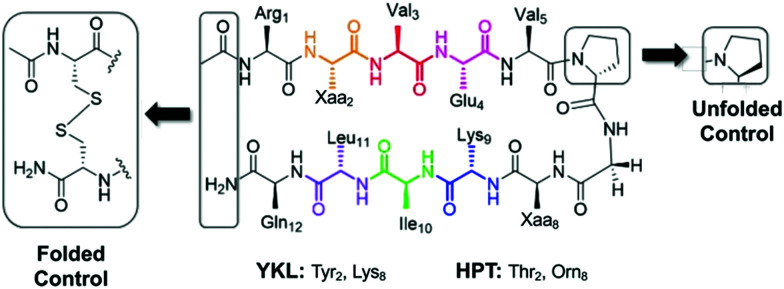
YKL and HPT test β-hairpin scaffolds. The unfolded control has a ProGly β-turn. The folded control has terminal cysteines that are oxidized to form a disulfide linked cyclic peptide. The folded control was only synthesized for the HPT scaffold.

In choosing a host scaffold for our thioamide guests, we analyzed β-hairpins that are water soluble, monomeric, and have significant β-sheet character. A well-established construct that meets these characteristics is the Gellman “YKL” β-hairpin.^38,39^ This scaffold has been utilized to study the β-hairpin stabilization of cross-strand interactions^38^ and strand length,^40^ as well as the β-sheet propensity of charged derivatives of β-branched-amino acids^41^ and aza-amino acids.^42^ This extensively studied scaffold is also a good starting point for structural characterization as several previous studies have reported structural models based on NMR data.^38–40,43^ This β-hairpin is enforced with a stabilized two-residue β-turn, proGly (where pro is d-proline). Examination of 2 residue β-hairpin loops in proteins determined that there is a preference for type I′ and type II′ β-turns^44^ and pro promotes the right-handed twist needed for this biologically relevant β-turn.^45^ Conversely, ProGly incorporation results in a left-handed turn that eradicates the β-sheet structure, which can be utilized for synthesis of an unfolded control peptide.^39^ This 12 residue anti-parallel “YKL” β-sheet is stabilized by a diagonal cation–π interaction (Tyr_2_ and Lys_9_). To avoid electrostatic associations from the termini, the N-terminus is acetylated, and the C-terminus is a carboxamide (Fig. 2). We hypothesized that the β-hairpin would be stabilized when the thioamide is positioned as a hydrogen bond donor.

### YKL scaffold CSD analysis

We initially chose to incorporate thioamides at the following hydrogen bond donor positions: Glu_4_, Lys_9_ and Leu_11_. These constructs are denoted YKL-Glu^S^_4_, YKL-Lys^S^_9_, and YKL-Leu^S^_11_-OH, using the superscript S convention for naming thioamides from Mahanta *et al.*^16^ The Leu^S^_11_ peptide was synthesized with a C-terminal carboxylate (indicated as –OH) due to the propensity for thioamides at the penultimate position to cause hydrolysis and epimerization at C-terminal amides (Fig. S4, ESI†).^3^ For each thioamide position of interest as well as the all-amide reference peptide denoted “YKL”, two peptides were synthesized, the test peptide (with proGly at the turn) and the corresponding unfolded control peptide. The unfolded control has the same sequence as the test peptide but has a ProGly β-turn so it does not have any β-sheet secondary structure.

Except for Glu^S^_4_, the test peptides displayed significant anti-parallel β-sheet character (minimum at 215 nm) in the far-UV region of their CD spectra (Fig. S6A, ESI†). This indicated that the level of folding in the thioamide peptides was comparable to the YKL reference peptide. For YKL-Glu^S^_4_, the π-to-π* absorbance of the thioamide has a greater intensity, which complicates the spectra and could explain the lack of a minimum at 215 nm. Since the contribution of a thioamide residue to the CD spectrum is not well-defined, we sought other measurements to quantify the relative stabilities of the thioamide variants. CD thermal melts measured at the 215 nm signature were linear, and therefore could not be fit to two-state models to derive folding energetics (Fig. S6B, ESI†). Therefore, we turned to 2D NMR measurements instead. Peptides were dissolved in sodium deuteroacetate buffer (Table S3, ESI†)‡‡For NMR experiments, the YKL peptides were dissolved in 100 mM sodium deuteroacetate buffer pH 3.8 (9 : 1 v/v H_2_O/D_2_O). The HPT peptides were dissolved in 50 mM sodium deuteroacetate pH 5.5 (9 : 1 v/v H_2_O/D_2_O) or 50 mM NaH_2_PO_4_ pH 5.5 (9 : 1 v/v H_2_O/D_2_O). Variability in solubility based on construct and experimental time required for data collection is the reason different buffers and pH values were used. Since the buffer remained consistent for the test, unfolded control, and folded control peptides of each HPT thioamide position, the difference in salt should have minimal to no effect on fraction folded and ΔΔ*G* analysis. Discussion of NMR collection across the different universities can be found in Table S3 and Fig. S15 (ESI†). and TOCSY and ROESY were collected at 10 °C. Lateral NOEs between Tyr_2_ and Leu_11_, as well as diagonal NOEs between Tyr_2_ and Lys_9_ were observed for all test β-hairpins (Fig. S7, ESI†). This suggests that all thioamide-containing variants are in a β-hairpin conformation. Although the connectivities of the observed NOEs differ slightly, the similarity of the overall patterns is enough to deem the structures alike.

**Fig. 3:**
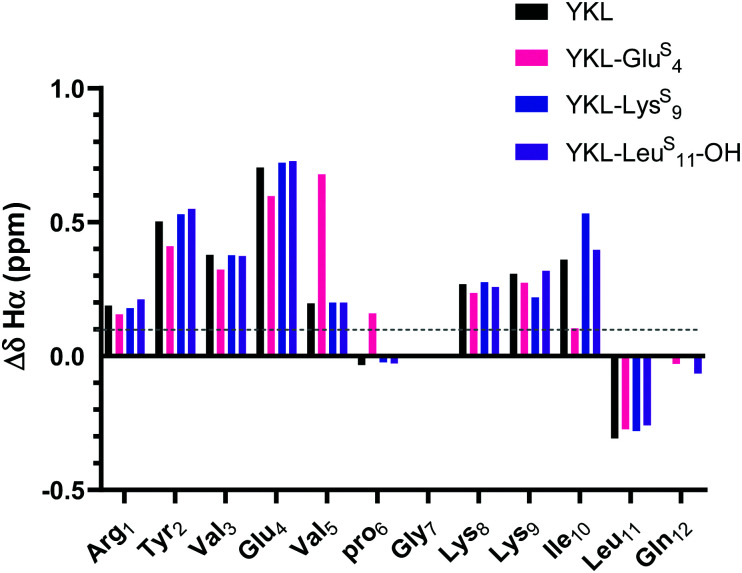
Δ*δ*_Hα_ (test *δ*_Hα_ − unfolded control *δ*_Hα_) for YKL-Glu^S^_4_, YKL-Lys^S^_9_, and YKL-Leu^S^_11_-OH in comparison to YKL. Three consecutive Δ*δ*_Hα_ values of greater than 0.1 ppm (shown in figure) is indicative of β-sheet structure. Besides slight variations around where the thioamide is incorporated, the Δ*δ*_Hα_ values are like YKL for all positions tested suggesting that the thioamide is not increasing foldedness. Δ*δ*_NH_ analysis also demonstrates the same trends (Fig. S9, ESI†).

As expected, the unfolded controls lack cross-strand NOEs (Fig. S8, ESI†). Inclusion of the ProGly β-hairpin allows calculation of the chemical shift deviation (CSD or Δ*δ* = test *δ* − random coil *δ*) with the ProGly unfolded control (Δ*δ* = test *δ* − unfolded control *δ*). β-Hairpins are dynamic structures, and CSD analysis is reflective of the global average, providing a quantitative measure indicative of secondary structure. A Δ*δ*_Hα_ of greater than 0.1 ppm for three consecutive residues is considered to be evidence of a β-sheet.^46^ The Δ*δ*_Hα_ data suggest that all tested thioamide-peptides have β-sheet character comparable to the YKL reference peptide (Fig. 3). At each thioamide position, the Δ*δ*_Hα_ for the *n* + 1 residue is significantly different in comparison to other peptides. However, this is merely indicative of a local perturbation of the electronic environment since Δ*δ*_Hα_ is not increased for other residues. Although YKL-Glu^S^_4_ has β-sheet character according to Δ*δ*_Hα_, the values are lower than the other positions tested. This could be due to additional conformational rigidity from the nearby β-turn, which results in a less favorable β-hairpin structure with a thioamide at Glu_4_. Thus, we conclude from these data as well as variable temperature NMR experiments that the thioamide hydrogen bond donor substitutions do not significantly increase the stability of the β-hairpin (Table S4, ESI†).

### HPT scaffold CSD analysis

In light of the unexpected failure of the thioamide hydrogen bond donor to increase foldedness, we questioned whether the YKL scaffold was too stable to observe potential perturbations to structure due to thioamide incorporation. Removal of the cation–π interaction would decrease the stability of the scaffold, making it more sensitive to perturbation. Indeed, the Cheng lab previously replaced Tyr_2_ with Thr to achieve this purpose and used Orn_8_ instead of Lys_8_ to help with chemical shift assignment.^47–49^ Also, the internal Tyr side-chain causes ring-currents that result in upfield shifts of other internal protons. Therefore, removal of Tyr additionally leaves the internal chemical shifts unaffected by the ring-currents and allows for more accurate determination of the effect of incorporation of an internal thioamide. This variant of the YKL scaffold is referred to as HPT (HairPins with Thr at position 2) (Fig. 2). The hypothesis remained that incorporation of a thioamide as a hydrogen bond donor would increase foldedness, whereas a thioamide as a hydrogen bond acceptor would decrease foldedness.

Thioamides were incorporated at Thr_2_, Val_3_, Ile_10_, and Leu_11_ in both test (proGly) and unfolded (ProGly) forms: HPT-Thr^S^_2_, HPT-Val^S^_3_, HPT-Ile^S^_10_, and HPT-Leu^S^_11_-OH. Again, a carboxylate was included at the C-terminus of the Leu^S^_11_ peptide, in this case due to hydrolysis of the amide form (Fig. S4, S5 and Tables S6, S7, ESI†). The test β-hairpins were first examined by CD and have a significant thioamide π-to-π* absorption band (Fig. S10, ESI†). However, the less stable test HPT peptides do not have as strong a β-sheet signature at 218 nm, which is further complicated by the strong thioamide absorbance. Therefore, the effect of thioamide incorporation on global secondary structure could not be determined using CD. As a result, we again relied on NMR for information.

HPT, HPT-Thr^S^_2_, HPT-Val^S^_3_, HPT-Ile^S^_10_, and HPT-Leu^S^_11_-OH were dissolved in sodium deuteroacetate or phosphate buffer to 1–10 mM concentration (Table S3, ESI†).^2^ TOCSY and ROESY spectra were collected at 25 °C. The HPT NMR data was obtained from a previous publication.^49^

For the test peptide, the HPT β-hairpin has both lateral NOEs between Thr_2_ and Leu_11_, as well as diagonal NOEs between Thr_2_ and Lys_9_. All thioamide test β-hairpins have mainly lateral NOEs between Glu_4_ and Lys_9_, with HPT-Thr^S^_2_ having a second cross-strand NOE between Thr_2_ and Leu_11_ (Fig. S11, ESI†). The presence of cross-strand NOEs for all positions tested is suggestive of β-hairpin conformation. As expected, all unfolded controls lack cross-strand NOEs (Fig. S12, ESI†). For a more quantitative measure of β-hairpin structure, we again used CSD analysis.

**Fig. 4:**
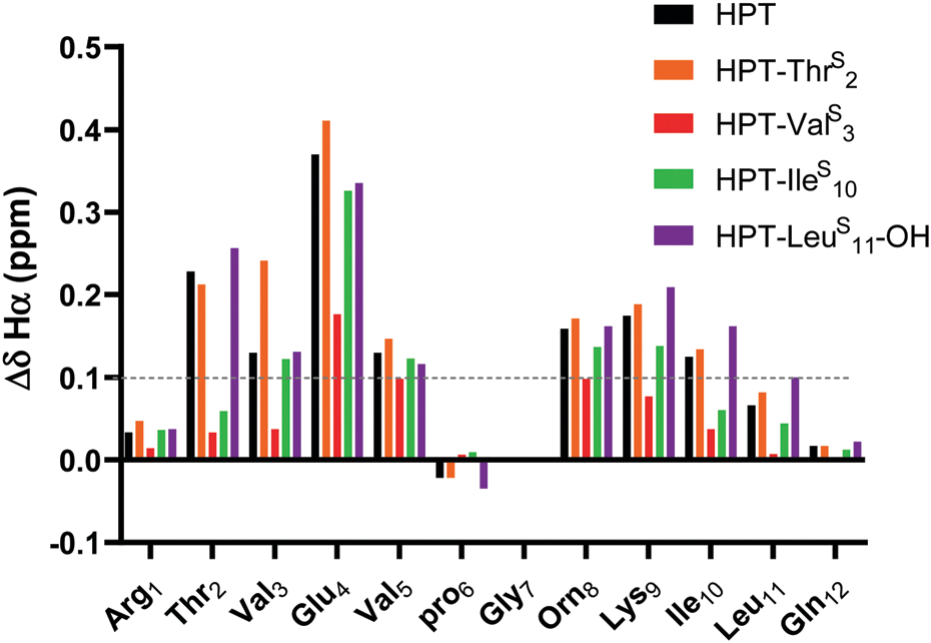
Δ*δ*_Hα_ (test *δ*_Hα_ − unfolded control *δ*_Hα_) for HPT-Thr^S^_2_, HPT-Val^S^_3_, HPT-Ile^S^_10_, and HPT-Leu^S^_11_-OH in comparison to HPT. Three consecutive Δ*δ*_Hα_ values of greater than 0.1 ppm (shown in figure) is indicative of β-sheet structure. The Δ*δ*_Hα_ data demonstrates that HPT-Thr^S^_2_, HPT-Ile^S^_10_, and HPT-Leu^S^_11_-OH have β-sheet character, whereas HPT-Val^S^_3_ does not. Discussion of Δ*δ*_NH_ is in the ESI† (Fig. S13).

The Δ*δ*_Hα_ values for HPT are generally lower than those for YKL, indicating that it has less β-sheet structure, in agreement with the CD data and expectations based on prior literature.^49^ The Δ*δ*_Hα_ values for the HPT-Thr^S^_2_, HPT-Ile^S^_10_ and HPT-Leu^S^_11_-OH peptides demonstrate that all have β-sheet structure, whereas HPT-Val^S^_3_ does not (Fig. 4). Besides HPT-Ile^S^_10_, these observations agree with our YKL work that a thioamide as a hydrogen bond donor is neutral, and additionally agrees with our hypothesis that an internal thioamide is destabilizing. We did not expect that an internal thioamide (Ile^S^_10_) could be minimally perturbing. The difference observed for both internal thioamide positions (Val^S^_3_ and Ile^S^_10_) could be due to the right-handed twist of the β-hairpin,^50^ which would better accept the additional steric bulk at Ile_10_, but not Val_3_.

### HPT scaffold ΔΔ*G* analysis

**Fig. 5:**
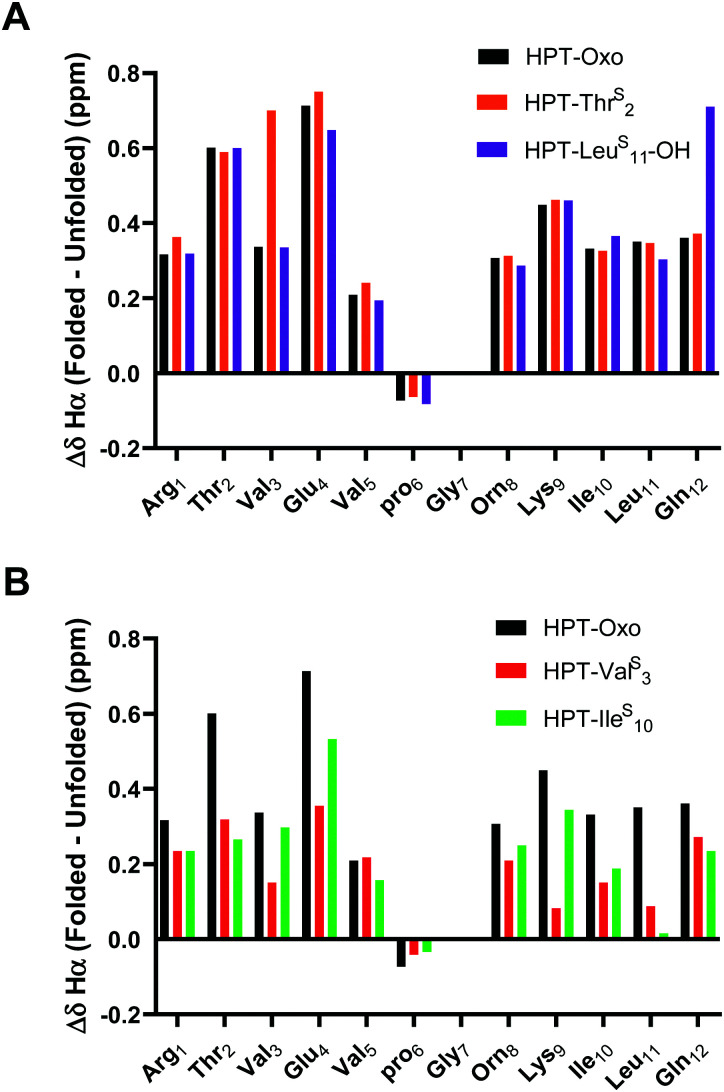
Δ*δ*_Hα_ (folded *δ*_Hα_ − unfolded control *δ*_Hα_) for external thioamides (A, HPT-Thr^S^_2_ and HPT-Leu^S^_11_-OH) and internal thioamides (B, HPT-Val^S^_3_ and HPT-Ile^S^_10_) in comparison to HPT. Folded controls with internal thioamides result in a decreased chemical shift dispersion. The dispersion can be quantified by comparing the Δ*δ*_Hα_ between the folded and unfolded controls. For the external thioamides (A), there is a similar dispersion observed for all the peptides. This is not observed for the internal thioamides (B), where the values greatly differ in comparison to HPT.

To quantitatively compare the effect of thioamide incorporation, folded controls were synthesized to allow for calculation of fraction folded (%) and ΔΔ*G*_Folding_. For the folded control, terminal cysteines were added and oxidized to produce a disulfide-linked cyclic peptide following a strategy previously employed by the Cheng laboratory (Fig. 2).^43,47–49^ However, this analysis is only valid if the folded controls display sufficient chemical shift dispersion. As the β-hairpin is more folded, both *δ*_Hα_ and *δ*_NH_ should be shifted downfield. This was observed when the thioamide is externally facing (*i.e.*, positioned as a hydrogen bond donor). However, the folded controls with an internal thioamide did not display enough dispersion to be a true folded control (Fig. 5). This could be due to puckering as the internal electron-rich thioamide is forced towards the opposing strand due to steric constraints. Since we observed abundant cross-strand NOEs for the HPT-Val^S^_3_ and HPT-Ile^S^_10_ folded controls (Fig. S14, ESI†), we performed fraction folded analysis despite the lack of chemical shift dispersion. We note that in Fig. 5 Δ*δ*_Hα_ is dramatically affected for the residue following the thioamide, consistent with previous studies of the steric and electronic impact of thioamide incorporation on neighboring residues.^51,52^ The Δ*δ*_Hα_ value is increased relative to the HPT value for more stable peptides with externally facing thioamides and decreased for less stable peptides with internally facing thioamides.

Fraction folded was calculated for each residue of a test β-hairpin using eqn (1). The final fraction folded value reported is an average of position 3 and 10. These positions were chosen because Val_3_ and Ile_10_ are in the middle of the β-strands and therefore not affected by the flexible termini or the constrained β-turn. Secondly, for Val_3_ and Ile_10_, Hα is externally facing and therefore *δ*_Hα_ is not affected by the internal micro-environment of the β-hairpin which may be perturbed by the thioamide. Thus, *δ*_Hα_ for these positions is a direct indicator of foldedness. Similar to Δ*δ*_Hα_ analysis, HPT-Thr^S^_2_ has the same fraction folded value as HPT (Table 1). HPT-Leu^S^_11_-OH has a higher fraction folded, HPT-Ile^S^_10_ is slightly less folded, and HPT-Val^S^_3_ is the least folded. ΔΔ*G*_Folding_ was calculated with eqn (2) and (3). The ΔΔ*G*_Folding_ provide a general metric that can be compared to other β-hairpin studies to place the magnitude of the observed effects in context.1
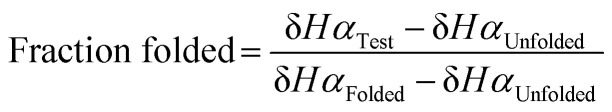
2
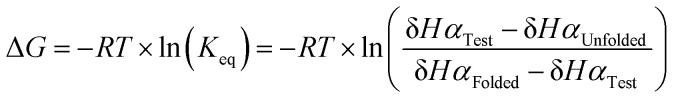

3 ΔΔ*G* = Δ*G*_Thio_ − Δ*G*_oxo_

**Table 1:** Fraction folded and ΔΔ*G*_Folding_ for HPT β-hairpins

Peptide	Fraction folded (%)	ΔΔ*G*_Folding_ (kcal mol^−1^)
HPT	38 ± 1	—
HPT-Thr^S^_2_	38 ± 3	0.01 ± 0.1
HPT-Val^S^_3_	25 ± 1	0.38 ± 0.01
HPT-Ile^S^_10_	37 ± 5	0.04 ± 0.1
HPT-Leu^S^_11_-OH	42 ± 3	−0.09 ± 0.1

These data clearly show that even in a short β-hairpin the context of the thioamide substitution is very important. While the thioamide as hydrogen bond acceptor can indeed be destabilizing (HPT-Val^S^_3_), it can also be a neutral modification (HPT-Ile^S^_10_). Likewise, while the thioamide as hydrogen bond donor can be stabilizing in the more sensitive HPT scaffold (HPT-Leu^S^_11_-OH), it too can be neutral (HPT-Thr^S^_2_), depending on local context.

### Structural modelling

To elucidate the mechanistic basis of the thioamide effects we observed with NMR, we utilized structural modelling with PyRosetta.^53^ Since the folded HPT peptides exhibited significantly stronger NOEs (Fig. S11 and S14, ESI†) due to their macrocyclic constraint, we took advantage of this stability to avoid difficulty in modelling and analysis because of the flexibility in the test peptides. As a starting point, we used the average NMR structure (PDB ID 1jy9) previously solved for a YKL derivative with four additional Thr residues at the termini (see Steric interactions sub-section below).^40^ The structure was modified to convert it to the HPT folded control by removing the two terminal Thr residues, converting the penultimate Thr residues to Cys, forming the disulfide bond, acetylating the N-terminus, converting the C-terminus to a carboxamide, and converting Tyr_2_ to Thr and Lys_8_ to Orn. Following generation in PyRosetta, the initial HPT structure is similar to the starting 1jy9 PDB structure (Fig. S16, ESI†). Next, we performed a constrained relax in Rosetta using the NOE-derived distances for the HPT folded control to generate our final HPT structure (Fig. S17 and S18, ESI†). Previously, the Petersson laboratory developed Rosetta patches for the thioamide based on *ab initio* calculations and experimental data for thioamides in protease substrates.^54–56^ With the thioamide patches and the corresponding distance constraints, the thioamide folded control peptides: HPT-Thr^S^_2_, HPT-Val^S^_3_, HPT-Ile^S^_10_, and HPT-Leu^S^_10_ were simulated in PyRosetta. On average, 20 constraints were used per structure and only three distance pairs per β-hairpin had a deviation of greater than 0.15 Å between the experimentally-derived and computed distances (Table S9, ESI†).

**Fig. 6:**
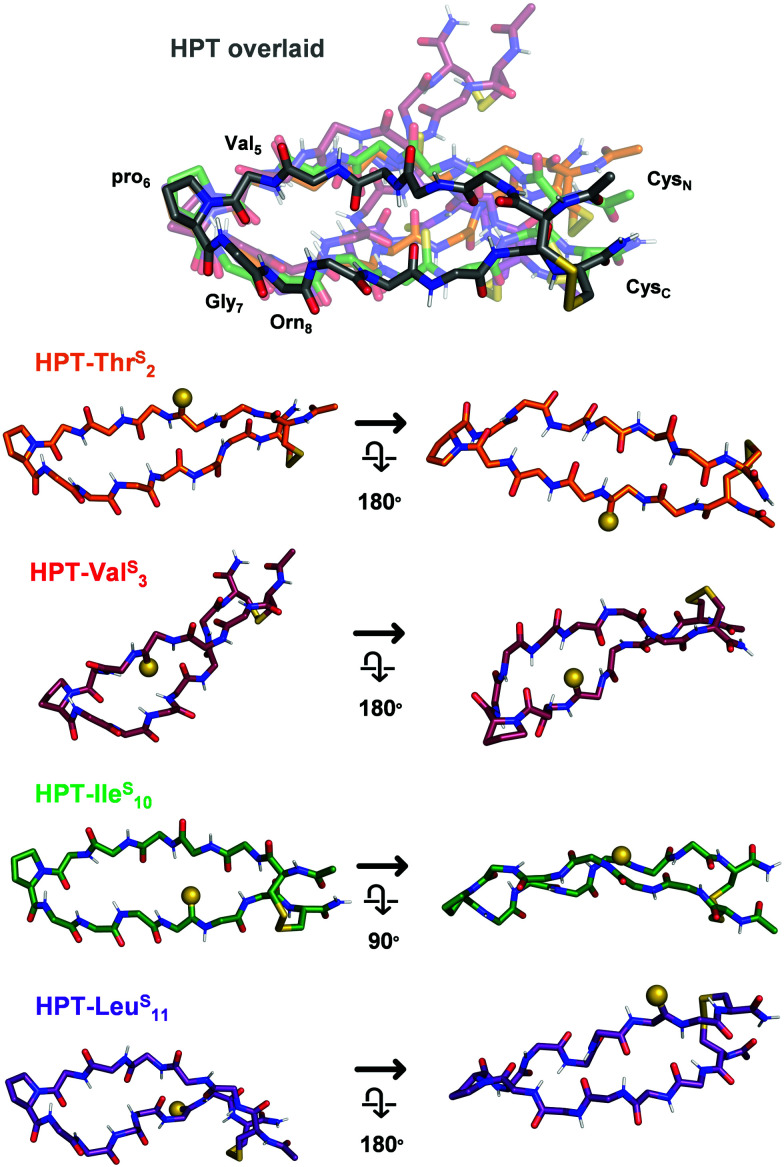
Structural models of the HPT folded control peptides. Only the backbone is displayed, except for pro_6_ and the terminal Cys residues. In HPT overlaid, all of the thioamide HPT peptides are aligned to HPT (grey, other peptides in indicated colors) based on the coordinates of Val_5_, pro_6_, Gly_7_, and Orn_8_ to enable comparison (RMSDs in Table S10, ESI†). Each thioamide peptide structure from the overlay is displayed individually from two angles, with the thioamide shown as a sphere. HPT-Thr^S^_2_ and HPT-Ile^S^_10_ are similar in structure to HPT. As a result of the right-hand twist of the β-hairpin, the thioamide for HPT-Ile^S^_10_ is more solvent-exposed. HPT-Val^S^_3_ has a dramatic twist, with differences in backbone arrangement around the internal thioamide. HPT-Leu^S^_11_ has a more pronounced twist at the terminus near the thioamide. Additional views and discussion of structures are in Fig. S18–S22 (ESI†).

The HPT-Val^S^_3_ folded control structure deviates greatly from the HPT folded control with a backbone root mean squared deviation (RMSD) of 2.15 Å (Fig. 6 and Fig. S20, Table S10, ESI†). Although the structure near the turn overlays well with the HPT folded control, accommodation of the internal thioamide at Val_3_ results in a dramatic twist in the hairpin at Thr_2_/Leu_11_. For the non-perturbing HPT-Thr^S^_2_ and HPT-Ile^S^_10_ folded macrocycles, the structures of both are more like the HPT folded peptides (Fig. 6 and Fig. S19, S21, Table S10, ESI†). The C-terminal strand for HPT-Thr^S^_2_ is closer to the N-terminal strand (hydrogen bonds among the four terminal residues are 0.5 Å shorter), potentially a result of the thioamide acting as a stronger hydrogen bond donor to the carbonyl of Ile_10_ as well as a flip of the Thr_2_ side-chain due to breaking of a hydrogen bond with the Thr_2_ carbonyl (Fig. S19, ESI†). In agreement with our proposed hypothesis, the internal thioamide at Ile_10_ is better accepted due to the right-handed twist of this scaffold, allowing for a longer hydrogen bond with the Val_3_ N–H without altering the hairpin shape (RMSD = 0.63 Å). For the slightly stabilized HPT-Leu^S^_11_, the overall structure is like the HPT folded control (RMSD = 1.23 Å), with a nearly identical structure near the hairpin turn, but with an additional twist at the terminus near the thioamide (Fig. 6 and Fig. S22, Table S10, ESI†). Although the Leu_11_–N–H/Arg_1_C=O hydrogen bond distance does not change, the angle does, leading to a rotation of the Leu_11_ side-chain and movement relative to the Cys_C_/Cys_N_ disulfide bond. Experimentally, this can be seen from an increase in the Leu_11_ and Cys_C_/Cys_N_ NOE distances (Table S10, ESI†).

To further analyze these macrocyclic peptide structures, we used a Backrub^57^ protocol to generate ensembles. With slightly higher average deviations from the experimentally-derived distances (Table S11, ESI†), these ensembles (except for HPT-Ile^S^_10_) demonstrate low backbone RMSDs (<1 Å) for the 10 lowest energy structures (Fig. S23–S26 (ESI†), links to coordinate files in pdb format are also provided), and align well with the constrained relax structures. For the HPT-Ile^S^_10_ folded macrocycle, there is increased rotation at the β-turn and the strand opposite the thioamide. This provides a potential mechanism for how the corresponding test peptide can accommodate the internal thioamide (Fig. S25, ESI†).

We note that these mechanistic explanations must be taken with some caution as a relatively small number of NOEs were available for modelling constraints. Additionally, the chemical shift dispersion is very small for HPT-Val^S^_3_, where the folded control structure deviates significantly from HPT (backbone RMSD of 2.15 Å), raising some concern over whether it is a true folded control. While these structures provide snapshots of the folded control structures, simulation of the unfolded and test peptides would be required for direct comparison of energetics to the experimental results. However, by simulating these structures we were able to provide plausible mechanistic explanations for how Ile^S^_10_ is well-accepted because of the right-hand twist, whereas Val^S^_3_ is destabilizing and results in a different configuration than HPT.

## Discussion

**Fig. 7:**
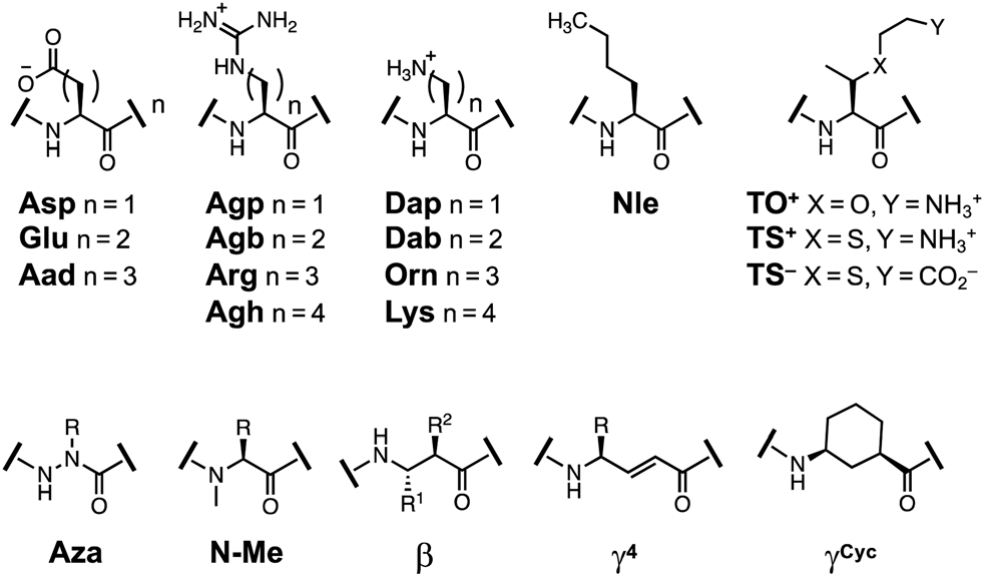
Structures of amino acids derivatives previously studied in β-hairpin scaffolds.

As noted, we chose the YKL/HPT scaffold because multiple studies have used it as a host system to investigate the β-sheet propensity of various amino acids and their derivatives. These studies have included a variety of non-covalent interactions such as ion-pairing, π-system interactions, and steric constraints. A review of these findings which utilize the amino acid derivatives shown in Fig. 7 can be found in the ESI.† To enable accurate comparisons, all stability measurements are reported as ΔΔ*G*_Folding_ with the parent peptide as a reference.

To place the thioamide modification in the context of the field, we found the following interactions important to mention. Strengthening cation–π interactions by methylation of Lys_9_ or Arg_9_ side-chains stabilized the hairpin by about −0.2 kcal mol^−1^ per methylation.^58–61^ Sulfur–arene interactions^62^ increased the stability by −0.3 to −0.5 kcal mol^−1^.^63^ Side-chain phosphorylation demonstrated that anion–π interactions were destabilizing by ∼1 kcal mol^−1^.^64,65^ Introduction of charged β-branched derivatives (TS_4_^−^ and TS_9_^+^) was highly stabilizing (−0.5 kcal mol^−1^ and −0.6 kcal mol^−1^), whereas TO^+^ was slightly destabilizing (+0.1 kcal mol^−1^).^41^ Addition of β-branched residues to the termini increased stability by −0.3 kcal mol^−1^.^40^ For backbone derivatives, Aza-Val_3_ incorporation was disruptive to foldedness (1.26 kcal mol^−1^), whereas aza-Gly_3_ was better accepted (0.75 kcal mol^−1^), but still less stable than YKL.^42^ β-Amino acid or linear (*E*)-vinylogous γ^4^-residues substitution was moderately destabilizing (0.5–0.6 kcal mol^−1^).^66,67^ Whereas a cyclically constrained γ-residue was stabilizing (−0.3 to −0.6 kcal mol^−1^).^67,68^ These perturbation studies are summarized in Table 2.

**Table 2:** Summary of YKL/HPT β-hairpin perturbation studies

Interaction	Perturbation^ref.^	Effect on ΔΔ*G*_Folding_
Cation–π	Methylation of Lys_9_ across from Trp_2_^58^	−0.2 kcal mol^−1^ per methylation
	Replacement of Lys_9_ with Arg across from Trp_2_^61^	−0.3 kcal mol^−1^
	Methylation of Arg_9_ across from Trp_2_^59^	−0.6 kcal mol^−1^ for first methylation
Sulfur–arene	Replacement of Lys_9_ with Met across from Trp_2_ or Phe_2_^63^	−0.3 to −0.5 kcal mol^−1^
Anion–π	Phosphorylated Ser_9_, Thr_9_, or Tyr_9_ across from Trp_2_^64,65^	+∼1 kcal mol^−1^
Steric	2 Thr added to each terminus^40^	−0.3 kcal mol^−1^
	TS_4_^−^ substitution^41^	−0.5 kcal mol^−1^
	TS_9_^+^ substitution^41^	−0.6 kcal mol^−1^
	TO_9_^+^ substitution^41^	+0.1 kcal mol^−1^
Backbone	γ^Cyc^ substitution^67,68^	−0.3 to −0.6 kcal mol^−1^
	γ^4^ substitution^67^	+0.5 kcal mol^−1^
	β-Amino acid replacement of two α-amino acids^66^	+0.5 to 0.6 kcal mol^−1^ per αα substitution
	Aza-Gly_3_ substitution^42^	+0.8 kcal mol^−1^
	Aza-Val_3_ substitution^42^	+1.3 kcal mol^−1^

Thioamide effects are comparable in scale to these previous modifications. HPT-Thr^S^_2_, HPT-Ile^S^_10_, and HPT-Leu^S^_11_-OH demonstrate a similar energy of folding to HPT (−0.09 to +0.04 kcal mol^−1^) where thioamide incorporation is less stabilizing than a cation–π or ion-pairing interaction. The internal thioamide at HPT-Val^S^_3_ is the most disruptive (0.38 kcal mol^−1^), but is still not as disruptive as β-amino acid incorporation (0.5–0.6 kcal mol^−1^) or phosphorylation (1 kcal mol^−1^).

There are differing opinions as to the relative importance of backbone hydrogen bonds, side-chain electrostatic and/or hydrophobic interactions on β-hairpin stability, and their importance can change depending on the β-hairpin construct.^69–71^ For the scaffolds we have discussed, it appears as though hydrophobic interactions such as aromatic stacking or addition of the β-branched derivatives are more stabilizing than electrostatic interactions (ion-pairing, cation–π). The favorable hydrophobic interaction suggests that desolvation could play a major role in stability for the β-hairpin. Since the thioamide is less polar than the canonical amide bond, an internal thioamide could further stabilize a hydrophobic interaction. Indeed, a recent study by Chatterjee indicates that the altered desolvation of the thioamide contributes to stability in the Pin1 WW β-sheet system.^30^ Stabilization by desolvation of the thioamide would be most prominent for internal thioamides. However, in the structural modelling of the HPT folded controls with internal thioamides, the thioamides are within hydrogen bonding distance of the opposing strand and do not appear to be engaging with a hydrophobic pocket (Fig. S20 and S21, ESI†). It is important to note that the previously discussed hydrophobic interactions are between side-chains (or thioamide and a side-chain), therefore the same might not be true for the backbone. Also, these β-hairpins, particularly the less stable HPT scaffold, are flexible substrates lacking tertiary structure so the ability to stabilize *via* backbone desolvation is very limited.

Conformational rigidity and hydrogen bonding are both backbone properties that can influence the stability of this β-hairpin scaffold. The combination of these properties, as well as differences in the micro-environment of each residue result in a complex system that does not behave as predicted based on small molecule studies. Our results show that an external thioamide can be slightly stabilizing (HPT-Leu^S^_11_-OH), whereas an internal thioamide can be destabilizing (HPT-Val^S^_3_), as predicted. However, they also show that an internal thioamide can be neutral (HPT-Ile^S^_10_) without significantly altering the peptide structure. The fact that these trends do not match simple interpretations of the hydrogen bonding properties of the thioamide demonstrates that the effect of incorporation is position specific.

The results also reflect the importance of certain interactions at a position in the β-hairpin. The increased stability of HPT-Leu^S^_11_-OH where the thioamide is positioned as a hydrogen bond donor suggests that backbone hydrogen bonding is important at the terminus. The lack of change in stability for HPT-Thr^S^_2_ and all thioamide-containing YKL β-hairpins suggests that backbone hydrogen bonds are less impactful at this position in HPT and in the YKL scaffold. However, interactions cannot always be neatly parsed into backbone and side-chain effects. For example, in our model of HPT-Thr^S^_2_ we observe that breaking of a backbone C=O side-chain OH hydrogen bond upon thionation leads to a twist that contributes to overall stability (Fig. S19, ESI†). Observations such as these highlight the importance of structural data attained using the macrocyclized folded peptides in understanding the impact of thioamide modification in model peptides and the growing number of thioamide-containing natural products.

Previous experimental work has suggested that introduction of a thioamide reduces conformational flexibility,^72,73^ and theoretical studies demonstrate increased steric constraints for the *n* + 1 residue.^51,52,74,75^ Thioamide incorporation at a residue closer to the β-turn (Glu^S^_4_) in the YKL scaffold, has β-sheet character based on Δ*δ*_Hα_ analysis, but it is less prevalent than the other positions tested. The decreased stability of YKL-Glu^S^_4_ could be due to an inability of Val_5_ to accommodate the additional conformational restraint since it is already constrained by pro_6_ and the β-turn. This would also explain the dramatic twist observed in our structural modelling work for the HPT-Val^S^_3_ folded control. Since this macrocyclic construct is also sterically constrained by the disulfide, the twist occurs to relieve the rigidity imposed by the thioamide.

Even in the macrocyclic folded peptides, the overall stability derives from an interplay of interactions that vary by position, making it difficult to define a single causative feature for thioamide stability effects. However, the NMR-derived models of these macrocyclic systems enable one to apply more sophisticated electronic structure calculations^51^ to help to explain stability effects as well as observations such as the effect on the Δ*δ*_Hα_ value for the *n* + 1 residue. We will pursue such computational analysis in conjunction with additional structure determination efforts for constrained systems.

## Conclusions

The collection and analysis of ^1^H–^1^H NMR data for thioamide incorporation into two β-hairpin scaffolds, as well as structural modelling of the macrocyclic folded controls, suggests structural trends which deviate from expectations based on previous thioamide small molecule studies. For a stable scaffold, the YKL β-hairpin, incorporation of thioamides as hydrogen bond donors did not increase foldedness. Instead, all positions of incorporation demonstrated a similar structure to that of the YKL parent peptide. In a less stable scaffold, the HPT β-hairpin, thioamide incorporation had different structural impacts depending on position. Incorporation of a thioamide as a hydrogen bond donor was either minimally stabilizing (HPT-Leu^S^_11_-OH) or neutral (HPT-Thr^S^_2_). Conversely, incorporation as a hydrogen bond acceptor was either destabilizing (HPT-Val^S^_3_) or neutral (HPT-Ile^S^_10_). To elucidate why these two positions were different we performed structural modelling of the folded controls. The conformation of HPT-Val^S^_3_ is highly unlike the others as a result of structural alterations to accommodate the destabilizing internal thioamide. Conversely, the folded control of HPT-Ile^S^_10_ is similar in structure to HPT. In this position, the internal thioamide is more solvent exposed due to the right-handed twist of the β-hairpin, and therefore the thioamide steric bulk is better accommodated. This deviation from expectation based on the environment of the thioamide residue follows our previous observations with protein secondary structures.^29^

Our results reinforce the idea that it is difficult to develop simple rules regarding how thioamide modifications will impact β-sheet structure since specific details such as twists, conformational rigidity or the relative importance of those hydrogen bonding interactions will play a major role. Currently, for utilization of the thioamide as a non-perturbing biophysical probe in fluorescence quenching or CD experiments, we recommend consulting the wild-type protein structure and incorporating the thioamide at β-sheet locations where the residue does not engage as a hydrogen bond acceptor. Although we have observed here that hydrogen bond acceptor positions can be tolerated, they are best avoided until criteria for identifying tolerated positions are determined. The increases in stability observed to date for incorporation as a hydrogen bond donor are minimal and should not significantly alter protein folding. To realize computational models that are predictive of the structural impact of thioamide incorporation at a position in a protein, we will use macrocyclic peptides like those shown to be useful in structure determination here, as well as host–guest studies of peptide/protein complexes, to gather sufficient data for machine learning models similar to those that have been successful in our protease studies.^54^ Ultimately, we hope to be able to rationally design peptides containing single or even multiple thioamide substitutions, as well as full-sized proteins synthesized through SPPS and/or native chemical ligation.^3,5,30,33,76–80^

## Author contributions

K. E. F. synthesized and purified the HPT-Thr^S^_2_ and HPT-Val^S^_3_ peptides, collected CD data for all HPT peptides, and acquired NMR data for the HPT-Ile^S^_10_ and HPT-Leu^S^_11_-OH peptides. M. P. synthesized and purified the HPT-Ile^S^_10_, HPT-Leu^S^_10_-OH, and HPT peptides. C.-H. H. acquired the NMR data for the HPT-Thr^S^_2_ and HPT-Val^s^_3_ peptide and initially made assignments. H. V. and B. K. synthesized all YKL peptides, performed CD analysis, collected NMR data (including variable temperature studies), and made initial assignments. K. E. F. processed, assigned, and analyzed all NMR data (using the initial assignments for assistance). S. G. G. performed the structural modelling. K. E. F. and E. J. P. wrote the manuscript with input from all authors.

## Conflicts of interest

There are no conflicts to declare.

## Supplementary Material

CB-003-D1CB00229E-s001
