# Supplementary material for: Structural impact of thioamide incorporation into a β-hairpin
Source: RSC Chem Biol. 2022 Apr 5;3(5):582–91. doi: 10.1039/d1cb00229e (PMC9092430; doi:10.1039/d1cb00229e)
Supplement: CB-003-D1CB00229E-s001 [file CB-003-D1CB00229E-s001.pdf]

Electronic Supplementary Material (ESI) for RSC Chemical Biology.
This journal is © The Royal Society of Chemistry 2022

Electronic Supporting Information for

Structural impact of thioamide incorporation into a β-hairpin

Kristen E. Fiore,[a] Martijn J. Patist,[a] Sam Giannakoulias,[a] Cheng-Hsin Huang,[b] Hitesh Verma,[c] Bhavesh Khatri,[c] Richard P. Cheng,[b] Jayanta Chatterjee,[c] and E. James Petersson[a,*]

[a]*Department of Chemistry, University of Pennsylvania, 231 S. 34th Street, Philadelphia, 19104, USA*

[b]*Department of Chemistry, National Taiwan University, No 1, Sec 4, Roosevelt Road, Taipei 10617, Taiwan*

[c]*Molecular Biophysics Unit, Indian Institute of Science, Bangalore 560012, India*

Table of Contents

General Information 3
Synthesis and Characterization of Thioamide Precursors 4
  Synthesis and Purification of ThioThreonine (Fmoc-Thr^S-NBt) Precursor 4
Peptide Synthesis, Purification, and Characterization 6
  HPT 6
  YKL 7
Circular Dichroism 12
  HPT 12
  YKL 12
Nuclear Magnetic Resonance Spectroscopy 13
  HPT 13
  YKL 13
HPT-Leu$^S_{11}$-OH Test and Unfolded Epimerization 26
Structural Models of the Thioamide-Containing Folded Control β-Hairpins 39
Structural Ensembles of the Thioamide-Containing Folded Control β-Hairpins 51
Previous β-Hairpin Modification Studies 56
  Steric Interactions 56
  π-Interactions 56
  Ion-pairing 57
  Backbone 57
References 58

Supplemental Figures

Figure S1. Analytical HPLC of HPT β-hairpins 9
Figure S2. Analytical HPLC of YKL β-hairpins 10
Figure S3. ^{1}H NMR overlay of thioamide-containing Test YKL β-hairpins acquired at different temperatures.... 15
Figure S4. Proposed mechanism of Edman-type degradation observed for the HPT-Leu$^{S}_{11}$-OH Unfolded and Test β-hairpins 26
Figure S5. CSD analysis ($\delta_{H\alpha}$) of the HPT-Leu$^{S}_{11}$-OH epimers........ 27
Figure S6. Far-UV circular dichroism (CD) of thioamide-containing Test YKL β-hairpins 29
Figure S7. NOEs for Test YKL β-hairpins 30
Figure S8. NOEs for Unfolded YKL β-hairpins 31
Figure S9. $\Delta\delta_{NH}$ (Test δ_{NH} – Unfolded control δ_{NH}) for YKL: Glu$^{S}_{4}$, Lys$^{S}_{9}$, and Leu$^{S}_{11}$-OH 32
Figure S10. Far-UV circular dichroism (CD) of thioamide-containing HPT β-hairpins........ 32
Figure S11. NOEs for Test HPT β-hairpins........ 33
Figure S12. NOEs for Unfolded HPT β-hairpins........ 34
Figure S13. $\Delta\delta_{NH}$ (Test δ_{NH} – Unfolded control δ_{NH}) for HPT: Thr$^{S}_{2}$, Val$^{S}_{3}$, Ile$^{S}_{10}$ and Leu$^{S}_{11}$-OH 35
Figure S14. NOEs for Folded HPT β-hairpins........ 36
Figure S15. ROESY mixing time comparison for the HPT scaffold........ 37
Figure S16. Generation of HPT Folded control from YKL derivative........ 39
Figure S17. Constrained relax with NOEs to generate HPT Folded control structure 39
Figure S18. Modelled structure of HPT Folded control 46
Figure S19. Modelled structure of HPT-Thr$^{S}_{2}$ Folded control peptide........ 47
Figure S20. Modelled structure of HPT-Val$^{S}_{3}$ Folded control peptide........ 48
Figure S21. Modelled structure of HPT-Ile$^{S}_{10}$ Folded control peptide 49
Figure S22. Modelled structure of HPT-Leu$^{S}_{11}$ Folded control peptide 50
Figure S23. Ensemble generation of the HPT-Thr$^{S}_{2}$ Folded control peptide with the backrub protocol 52
Figure S24. Ensemble generation of the HPT-Val$^{S}_{3}$ Folded control peptide with the backrub protocol 53
Figure S25. Ensemble generation of the HPT-Ile$^{S}_{10}$ Folded control peptide with the backrub protocol........ 54
Figure S26. Ensemble generation of the HPT-Leu$^{S}_{11}$ Folded control peptide with the backrub protocol........ 55
Figure S27. Structure of amino acid derivatives........ 56

Supplemental Tables

Table S1. MALDI, gradient of purification, and analytical retention time of the synthesized β-hairpins 8
Table S2. HPLC gradients for purification 11
Table S3. Concentration of NMR sample........ 14
Table S4. Amide temperature coefficient (in ppb/K) measurements for YKL and its thioamidated analogs........ 17
Table S5. ^{1}H Chemical shift assignments for HPT and YKL β-hairpins........ 18
Table S6. Comparison of fraction folded and $\Delta G_{Folding}$ analysis for HPT-Leu$^{S}_{11}$-OH Test and Unfolded epimers. 27
Table S7. ^{1}H Chemical shift assignments for HPT-Leu$^{S}_{11}$-OH Test and Unfolded epimers........ 28
Table S8. Fraction folded and $\Delta\Delta G_{Folding}$ analysis of HPT peptides........ 38
Table S9. NOE derived distance constraints for PyRosetta simulation of Folded control β-hairpins........ 41
Table S10. Backbone RMSD of the thioamide-containing Folded control structures in comparison to the HPT Folded control 45
Table S11. Deviation, energy score and RMSD values for the backrub generated Folded control ensembles....... 51

Materials and Methods

General Information.

HPT peptides were synthesized, purified, and characterized at the University of Pennsylvania (Penn). Nuclear magnetic resonance (NMR) data for HPT peptides was collected at the National Taiwan University (NTU) (HPT-Thr^{S}_{2} and HPT-Val^{S}_{3}) or Penn (HPT-Ile^{S}_{10} and HPT-Leu^{S}_{11}-OH). NMR data for the HPT peptide was previously collected at NTU[1]. All YKL peptides were synthesized, purified, and characterized at the Indian Institute of Science (IIS). NMR data for YKL peptides was collected at IIS.

Reagents. *N*α-Fmoc-*N*δ-Boc-L-ornithine, Fmoc-D-proline, *N*α-Fmoc-*N*ω-(2,2,4,6,7-pentamethyldihydro-benzofuran-5-sulfonyl)-L-arginine, 7-Azabenzotriazol-1-yloxy)tripyrrolidino-phosphonium hexafluorophosphate (PyAOP), triisopropylsilane (TIPS) were purchased from ChemImpex (Wood Dale, IL, USA). All other Fmoc-protected amino acids and rink amide resin were purchased from Novabiochem (currently Millipore Sigma; St. Louis, MO, USA). α-cyano-4-hydroxycinnamic acid (CHCA) was purchased from Santa Cruz Biotechnology, Inc (Dallas, TX, USA). *N*-methylmorpholine (NMM) and 1,8-Diazabicyclo[5.4.0]undec-7-ene (DBU) were purchased from Acros (currently Fisher Scientific; Waltham, MA, USA). All other reagents and solvents were purchased from Fisher Scientific or Millipore Sigma unless otherwise specified. Milli-Q filtered (18 MΩ) water was used for all solutions. All reagents and solvents were used without further purification.

Instrumentation. Penn: Matrix-assisted laser desorption/ionization time-of-flight (MALDI-TOF) mass spectra were collected with a Bruker Ultraflex III (Billerica, MA, USA). High-resolution electrospray ionization mass spectra (ESI-HRMS) were collected with a Waters LCT Premier XE liquid chromatograph/ mass spectrometer (Milford, MA, USA). Low-resolution electrospray ionization mass spectra (ESI-LRMS) were obtained with a Waters single quadrupole detector (SQD) mass spectrometer with an Acquity Ultra Performance LC. NMR data were acquired with a Bruker AVANCE NEO 600 MHz spectrometer. Ultraviolet-visible (UV-vis) absorption spectra were collected on a GENESYS 150 UV-vis spectrophotometer (Thermo Fisher Scientific; Waltham, MA, USA). Circular dichroism (CD) data were acquired with a Jasco J-1500 CD spectrometer. NTU: NMR data were acquired on a Bruker AV III 800 MHz spectrometer. IIS: NMR data were acquired on a Bruker Avance 700 MHz spectrometer. CD data were acquired with a Jasco-715 spectropolarimeter

Synthesis and Characterization of Thioamide Precursors.

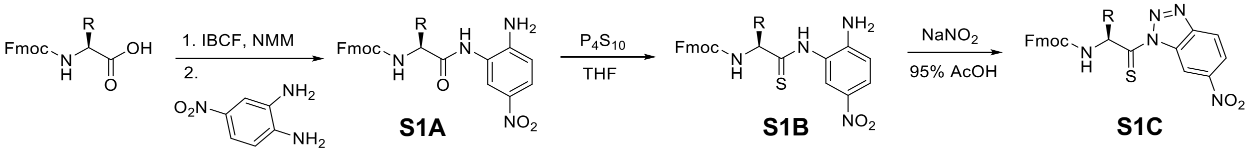

Scheme S1. General Fmoc-protected nitrobenzotriazolide thioamide precursor synthesis.

*N*α-Fmoc-L-valine-nitrobenzotriazolide was synthesized as previously reported.[2] *N*α-Fmoc-L-thioleucine-nitrobenzotriazolide[3] and *N*α-Fmoc-L-thioisoleucine-nitrobenzotriazolide[4] were synthesized as previously reported, with the following modifications.

Coupling of the Fmoc-L amino acids with 1,2-diamino-4-nitrobenzene (**S1A**). After addition of 4-nitro-*o*-phenylenediamine the reaction was stirred under argon for 2 hours at -10 °C and then at room temperature overnight. After removing the solvent *in vacuo*, the product was dissolved in DMF and crashed out with the addition of 1:1 saturated KCl/MilliQ H_2O. The precipitate was filtered and washed extensively with water. The residual water was removed overnight under high vacuum.

Preparation of the Fmoc L-amino thioacid nitroanilides (**S1B**). Phosphorous pentasulfide (P_4S_{10}) (0.75 eq) and anhydrous Na_2CO_3 (0.75 eq) were added to dry THF under argon and left stirring for 30 minutes (or until the phosphorous pentasulfide dissolved). After which, the compound **S1A** was added. The reaction was purged with argon and left to stir overnight. The next day, after removing the solvent *in vacuo*, the solid was resuspended in ethyl acetate and filtered over a pad of Celite©. The filtrate was washed twice with 5% $NaHCO_3$ and once with brine. The organic layers were combined and dried with $MgSO_4$. After filtration, the product was dry-loaded in silica or Celite© and purified over silica on a Biotage Isolera One system (Biotage, LLC, Charlotte, NC, USA) with ethyl acetate/*n*-hexanes (20-50% ethyl acetate in n-hexanes for thioisoleucine and 20-30% ethyl acetate in *n*-hexanes for thioleucine).

Preparation of Fmoc-L-amino thioacid nitrobenzotriazolides (**S1C**) After oxidation with $NaNO_2$ (1.5 eq) in 95% glacial acetic acid (v/v in Milli-Q water) the product was crashed out with cold MilliQ H_2O. The filtered precipitate was lyophilized overnight and used directly for SPPS without further purification.

Synthesis and Purification of ThioThreonine (Fmoc-Thr^S-NBt) Precursor.

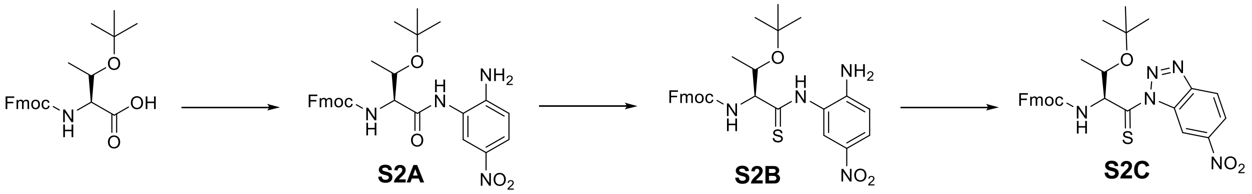

Scheme S2. Fmoc-Thr^S-Nbt (**S2C**) thioamide precursor synthesis.

(9*H*-fluoren-9-yl)methyl ((2S,3S)-1-((2-amino-5-nitrophenyl)amino)-3-(tert-butoxy)-1-oxobutan-2-yl) carbamate (S2A). Fmoc-Thr(*t*-Bu)-OH (1.987 g, 5.00 mmol) was dissolved in 40 mL of anhydrous tetrahydrofuran (THF) (dried over molecular sieves) under argon flow with stirring in an oven-dried round bottom flask. The solution was cooled to -10 °C in a 1:3 NaCl/ ice bath. *N*-methylmorpholine (NMM) (1.10 mL, 10.0 mmol, 2 eq) was added. While stirring, isobutyl chloroformate (IBCF) (0.648 mL, 5 mmol, 1 eq) was added dropwise. Residual IBCF in the syringe was deactivated by rinsing with saltwater from the ice bath. The reaction was stirred at 10 °C for 15 minutes. Afterwards, the 4-nitro-*o*-phenylenediamine (0.766 g, 5.00 mmol, 1 eq) was added. The reaction flask was purged with argon and left to stir for 2 hours at -10 °C and then at room temperature (RT) overnight under

argon. The next morning, the solution had turned orange. After removing the solvent *in vacuo*, the product was dissolved in 25 mL of DMF and precipitated upon addition to a saturated aqueous potassium chloride solution (250 mL). The precipitate was obtained by vacuum filtration with a Büchner funnel. After extensive washing with cold water, the solid was dried for 3-4 hours. Residual water was removed under high vacuum overnight. Compound **S2A** was obtained in 76.8% yield (2.045 g, 3.842 mmol). δH (600 MHz, DMSO) 9.20 (1 H, s), 8.26 (1 H, s), 7.88 (4 H, d, J 6.8), 7.77 (2 H, t, J 6.4), 7.41 (3 H, t, J 7.5), 7.33 (1 H, t, J 6.8), 7.25 (1 H, d, J 8.7), 6.80 (1 H, d, J 9.1), 6.54 (2 H, s), 4.39 – 4.30 (2 H, m), 4.26 (2 H, t, J 6.9), 4.07 – 4.01 (1 H, m), 3.89 (1 H, d, J 6.5), 1.17 (9 H, s), 1.13 – 1.09 (3H, m). A minor impurity due to removal of the t-butyl group resulted in the following additional peaks: 1.14 (3 H, s) and 0.93 (1 H, d, J 6.7). This impurity also resulted in the singlet at 1.17 and multiplet at 1.13-1.09 to have integrations higher than expected (10 and 6 respectively). δC (151 MHz, DMSO) 169.084, 156.075, 149.177, 143.891, 143.721, 140.742, 135.558, 128.911, 127.654, 127.274, 127.052, 125.371, 121.461, 121.369, 121.060, 120.106, 120.012, 113.609, 74.142, 67.394, 65.921, 59.946, 46.712, 28.319, 28.161, 27.876, 19.168, 18.904.

(9*H*-fluoren-9-yl)methyl ((2S,3S)-1-((2-amino-5-nitrophenyl)amino)-3-(tert-butoxy)-1-thioxobutan-2-yl) carbamate (S2B). Phosphorous pentasulfide (P_4S_{10}) (0.667 g, 1.50 mmol) and anhydrous sodium carbonate (0.159 g, 1.50 mmol) were added to an oven-dried round bottom flask. To the reaction flask, 20 mL of anhydrous THF dried over molecular sieves was added and allowed to stir under argon at room temperature until the phosphorous pentasulfide was dissolved (30-40 minutes). Compound **S2A** was added and stirred overnight at room temperature under argon. The next morning, the solvent was removed *in vacuo* to yield a yellow crystalline material. This product was resuspended in ethyl acetate and filtered over a pad of Celite© to remove insoluble phosphorous pentasulfide. The filtrate was washed twice with 5% $NaHCO_3$ (200 mL each) and once with brine (200 mL). The aqueous layer was back extracted with ethyl acetate. The organic layers were combined, dried with $MgSO_4$, and filtered. The product was dry-loaded in silica onto a 100 g column pre-equilibrated with 20% ethyl acetate and purified on a Biotage Isolera One system (20-50% ethyl acetate in *n*-hexanes). The solvent was removed under reduced pressure. The resulting yellow solid was dried under vacuum and stored at 4 °C. Compound **S2B** was obtained in 55.6% yield (0.610 g, 1.10 mol). δH (600 MHz, DMSO) 10.89 (1 H, s), 7.98 (2 H, d, J 7.0), 7.90 (2 H, d, J 7.6), 7.75 (2 H, dd, J 19.7, 7.5), 7.46 – 7.39 (2 H, m), 7.33 (2 H, td, J 7.4, 4.0), 7.05 (1 H, d, J 8.1), 6.82 (1 H, d, J 9.7), 6.44 (2 H, s), 4.55 (1 H, dd, J 8.3, 4.3), 4.36 (2 H, d, J 6.8), 4.27 (1 H, t, J 7.1), 4.10 (1 H, p, J 5.9), 1.21 (9 H, s), 1.17 (3 H, d, J 6.2). δC (151 MHz, DMSO) 202.663, 188.230, 155.741, 150.306, 143.595, 140.732, 135.280, 127.686, 127.075, 125.331, 125.279, 122.133, 120.140, 114.017, 74.301, 68.601, 66.042, 46.685, 44.641, 36.746, 28.011, 19.181.

(9*H*-fluoren-9-yl)methyl ((2S,3S)-3-(tert-butoxy)-1-(6-nitro-1H-benzo[d][1,2,3]triazol-1-yl)-1-thioxobutan-2-yl)carbamate (S2C, Fmoc-Thr^S-NBt). Compound **S2B** (0.378 g, 0.690 mmol) was dissolved in 10 mL of 95% glacial acetic acid (5% Milli-Q water) and cooled to 0 °C. $NaNO_2$ (71.4 mg, 1.03 mmol, 1.5 eq) was added slowly and the solution was stirred for 30 minutes at 0 °C (the $NaNO_2$ did not dissolve immediately). (The reaction was checked on to make sure it did not freeze, if frozen the solution was warmed by hand). After 30 minutes, a precipitant formed, and the solution was transferred to 100 mL of cold MilliQ water. The precipitated product was obtained by filtration with a Büchner funnel. After extensive washing with cold MilliQ water, the solid was dried for 3-4 hours and then residual water was removed via lyophilization. Compound **S2C** was obtained in 85.2% yield (0.329 g, 0.588 mmol) and used directly for SPPS without further purification. (Major rotamer. Overlay of peaks resulted in integration of singlet at 0.93 to be 10. The expected integration for the major rotamer is listed below) δH (600 MHz, $CDCl_3$) 9.66 (1 H, d, J 2.1), 8.44 (1 H, dd, J 8.9, 2.1), 8.31 (1 H, d, J 8.9), 7.78 (2 H, t, J 6.9), 7.68 (2 H, t, J 8.6), 7.42 (2 H, q, J 7.5), 7.38 – 7.32 (2 H, m), 7.11 (1 H, dd, J 5.6, 3.1), 4.55 (1 H, dd, J 10.8, 6.5), 4.44 – 4.34 (2 H, m), 4.28 (1 H, t, J 7.1), 0.93 (9 H, s), 0.81 (3 H, s). (Mixture of rotamers) δC (151 MHz, $CDCl_3$) 206.078, 156.532, 149.758, 148.832, 143.964, 143.824, 141.415, 131.925, 127.842, 127.212, 127.192, 125.294, 125.200, 122.290, 121.593, 120.116, 120.094, 112.705, 74.154, 69.994, 67.323, 67.152, 47.347, 28.236, 21.556.

Peptide Synthesis, Purification, and Characterization.

HPT

General. Peptides were manually synthesized via Fmoc solid phase peptide synthesis (SPPS) on rink amide resin (100-200 mesh, 0.54 mmol/g loading, NovaBioChem) in 12 mL fritted syringes. All peptides were synthesized on a 50 μmol scale, except for the HPT-Leu$^{S}_{11}$-OH peptides which were synthesized on a 100 μmol scale.

The resin was swelled by stirring in *N, N*-dimethylformamide (DMF) (6 mL, 30-45 minutes). After draining and rinsing with DMF (4 or 8 mL), the resin was deprotected with 20% v/v piperidine in DMF (2 or 4 mL) for 2 x 10 minutes. In-between all deprotections, the reaction vessel was washed with Wash 1 (4 or 8 mL each: DMF x2, methylene chloride (DCM), DMF). After the last deprotection, the vessel the washed with Wash 2 (4 or 8 mL each: (DMF, DCM) x 3, DMF). The first amino acid (Fmoc-Gln(Trt)-OH) (5 eq) and PyAOP (5 eq) were dissolved in DMF (2 mL or 4 mL) and DIPEA (10 eq) was added. After vortexing, the mixture was added to the vessel. After stirring for 30 minutes at room temperature and washing with DMF (4 or 8 mL), the coupling was repeated. The Fmoc group was removed with treatment of 20% v/v piperidine in DMF (2 or 4 mL) for 2 x 10 minutes. Wash 1 and Wash 2 were performed as described. This series of couplings and deprotections continued until the thioamide was to be coupled.

Thioamide Coupling and Deprotection. The nitrobenzotriazolide thioamide precursor (2.5 – 3 eq) was dissolved in anhydrous DCM over molecular sieves (purchased from Fisher) (2 mL). DIPEA (3 – 3.5 eq) was added, vortexed, and the mixture was added to the reaction vessel to stir at room temperature for 1 hour. After Wash 1, the coupling was repeated. The remaining unreacted termini were acetyl capped by treatment with 5 mL (8.4 mL DMF, 1.0 mL acetic anhydride, 0.6 mL NMM) for 2 x 10 minutes, with a DMF wash in-between. Following Wash 2, the Fmoc-group was removed with 2% DBU v/v in DMF (2 or 4 mL) for 3 x 2 minutes. After the first two DBU deprotections, Wash 1 was performed, and after the last deprotection, Wash 2 was performed.

The remaining amino acids were coupled as previously described; however, deprotections were performed with 2% DBU v/v in DMF (2 or 4 mL, 3x 5 minutes) to avoid epimerization and previously published side-reactions with piperidine.[5] Following removal of the Fmoc-group from Arg_1 (Test and Unfolded peptides) or Cys_1 (Folded peptides), the N-terminus was acetylated (treatment with 5 mL (8.4 mL DMF, 1.0 mL acetic anhydride, 0.6 mL NMM) for 2 x 10 minutes, with a DMF wash in-between). Wash 2 was performed to clean the final product, followed by three washes with DCM (4 or 8 mL). The vessel was washed with DCM and left under vacuum to dry the resulting resin.

Cleavage. For the Unfolded and Test peptides: a cleavage cocktail of 95% TFA, 2.5% TIPS, 2.5% MilliQ H_2O (v/v) (5 mL) was added to the vessel. For the Folded peptides: a cleavage cocktail of 90% TFA, 5% EDT, 2.5 % TIPS, 2.5% MilliQ H_2O (v/v) (5 mL) was added to the vessel. The vessel rotated at room temperature for 45-60 minutes. The cleavage solution was drained from the syringe and rinsed with DCM. After removal of the cleavage solution *in vacuo*, the peptide was washed with DCM and subsequently dried *in vacuo*. Cold diethyl ether (20 mL) was added slowly to the product and the suspended precipitate was transferred to a 50 mL Falcon tube. A second wash with diethyl ether (10 mL) was added to remove residual precipitated peptide. A third wash was performed (10 mL) if needed. The falcon tube was cooled in dry ice for 15 minutes, and then the precipitate was collected by centrifugation at 4,000 RPM (3,313 x *g*) for 5 minutes. The diethyl ether was carefully poured off, and the pellet was left to dry in the hood overnight. Afterwards the pellet was stored at 4 °C.

Purification. The crude peptide was dissolved in a minimal volume mixture of MilliQ H_2O + 0.1% TFA, methanol, and ≤ 20% acetonitrile (ACN) + 0.1% TFA and purified by RP-HPLC on either a Biotage Isolera one (Biotage, LLC, Charlotte, NC, USA) or on an Agilent 1260 Infinity II Preparative HPLC (Santa Clara, CA, USA) with a Biotage SNAP Ultra C18 column or a Luna Omega PS C18 preparative column (5 μm particle size, 250 mm length, 21.2 mm diameter) using the following gradients (**Table S2**). The desired peptide was identified with MALDI using a CHCA matrix, and was dried on a lyophilizer (Labconco; Kansas City, MO, USA). The dried peptide was

dissolved in MilliQ H_2O + 0.1% TFA and subject to second-pass RP-HPLC purification on the Agilent Preparative HPLC using one of two columns: Luna Omega PS C18 preparative column (5 µm particle size, 250 mm length, 21.2 mm diameter) or a Phenomenex Luna Omega PS C18 semi-preparative column (5 µm particle size, 250 mm length, 10 mm diameter) using the following gradients (**Table S2**). Purity of the peptides was assessed by analytical RP-HPLC on an Agilent 1260 Infinity II series Analytical HPLC system with a Phenomenex Luna Omega PS C18 column (5 µm particle size, 150 mm length, 4.6 mm diameter) with gradient **K**. Peptides for NMR studies were ≥ 95% pure (based on analytical 215 nm AUC integration).

Cyclization. Following second-pass purification, the dried folded peptides were dissolved in 0.1 M ammonium bicarbonate pH 8.0 until ≤ 100 µM concentration (as low as 50 µM was fine). The concentration was roughly checked by the UV-Vis absorbance measurement at 274 nm, and concentration was calculated based on the thioamide extinction coefficient ($\varepsilon_{274nm} = 10{,}169\ M^{-1}cm^{-1}$). The product was split into multiple 50 mL Falcon tubes so that the volume per tube was ≤ 20 mL. The solution was stirred vigorously under atmosphere and the cyclization was monitored with MALDI. The cyclization was complete within 6-9 hours and the product was dried via lyophilization. The product was subject to a final RP-HPLC purification on the Agilent Preparative HPLC with a Phenomenex Luna Omega PS C18 column using gradient **D**, **I** or **B** (**Table S2**).

YKL

General. Peptides were manually synthesized via Fmoc solid phase peptide synthesis (SPPS) on Rink Amide AM resin (0.8 $mmolg^{-1}$) on 200 mg scale (0.16 mmol) in 12 mL fritted syringes, except for the YKL-Leu^{S}_{11}-OH peptides which were synthesized on 2Cl-TCP resin (1.3 $mmolg^{-1}$).

The Rink Amide AM resin was swollen in DMF (20 min) and deprotected with 20% v/v piperidine in DMF (5 min x 1, 15 min x 1) followed by thorough washing with DMF (3 times). The C-terminal amino acid, Fmoc-Gln(Trt)-OH (2.5 eq) was loaded onto the resin by using standard coupling reagents (2.5 eq HOBt, 2.5 eq DIC) in DMF for 2 hours at room temperature. For the YKL-Leu^{S}_{11}-OH peptides, the C-terminal amino acid residue was loaded onto the resin with 2.5 eq DIPEA in anhydrous DCM at room temperature. After loading the first amino acid, the remaining unreacted trityl chloride groups bound to the solid-support were capped using methanol (200 µl/100 mg resin) for 15 min. Next, the resin was thoroughly washed with DCM (3 times), 1:1 DCM-methanol (3 times) and methanol (3 times) and finally dried under vacuum. The loading capacity was estimated from the dry weight of the resin, which ranged from 0.6-0.8 $mmolg^{-1}$. This series of couplings and deprotections continued until the thioamide was to be coupled.

Deprotections. Fmoc-deprotections for oxo-peptides were carried out with 20% v/v piperidine in DMF (5 min + 15 min) and 10% v/v piperidine in DMF (30 sec x 2) was used for thio-peptides after the incorporation of thionated amino acid to avoid epimerization.[6] The resin was washed two times with 3 ml of DMF for 60 sec between each cycle.

Cleavage and Purification. The global deprotection of protected peptide was carried out with 62% TFA, 31% DCM, 3.5% TIPS, 3.5% H_2O (v/v) for 30 min. After the deprotection, the deprotected peptide was precipitated in chilled ether yielding the crude product and further, it was purified by RP-HPLC with gradient **M** using a semi-preparative column (Phenomenex C18, 250 mm x 10 mm I.D., 5 µm) at a flow rate of 4 mL min^{-1} on a Shimadzu UFLC system (Kyoto, Japan). Purity of peptides was assessed on a C18 (Phenomenex, 100 mm x 4.6 mm) column at 1.0 ml/min flow rate using RP-HPLC with gradient **M**.

The detailed experimental procedure for the synthesis of nitrobenzotriazolide thioamide precursors and incorporation on solid support have been reported earlier.[7]

Table S1. MALDI characterization, gradient of purification, and analytical retention time of the synthesized β-hairpins

Peptide	[M+H]$^+$ (m/z)		[M+Na]$^+$ (m/z)		[M+K]$^+$ (m/z)		Gradient	Analytical Retention Time*
	Exp	Obs	Exp	Obs	Exp	Obs		
**HPT**								
Oxo Test	1394.85	1394.76	1416.83	1416.77	1432.80	1432.76	C	22.2
Oxo Unfolded	1394.85	1395.65	1416.83	1417.64	1432.80	1433.62	H G	20.4
Oxo Folded	1598.80	1598.83	1620.83	1620.93	1636.81	1636.89	J B	25.6
Thr$^{S}_{2}$ Test	1410.82	1410.73	1432.81	1432.71	1448.78		A B	23.5
Thr$^{S}_{2}$ Unfolded	1410.82	1410.86	1432.81	1432.84	1448.78	1448.81	A B	21.7
Thr$^{S}_{2}$ Folded	1614.83	1615.06	1636.81	1637.04	1652.78		A C D	22.9
Val$^{S}_{3}$ Test	1410.82	1410.94	1432.81	1432.92	1448.78		A D	25.0
Val$^{S}_{3}$ Unfolded	1410.82	1411.00	1432.81	1432.99	1448.78		A B	22.9
Val$^{S}_{3}$ Folded	1614.83	1615.10	1636.81	1637.08	1652.78		A C D	25.8
Ile$^{S}_{10}$ Test	1410.82	1410.71	1432.81	1432.78	1448.78	1448.77	H I	24.6
Ile$^{S}_{10}$ Unfolded	1410.82	1411.65	1432.81	1432.65	1448.78	1448.64	H G	22.2
Ile$^{S}_{10}$ Folded	1614.83	1614.66	1636.81	1636.69	1652.78		I	26.0
Leu$^{S}_{11}$-OH Test	1411.81	1411.78	1433.79	1433.76	1449.77	1449.74	E I B	25.4
Leu$^{S}_{11}$-OH Unfolded	1411.81	1411.78	1433.79	1433.77	1449.77		F B L	22.4
Leu$^{S}_{11}$ Folded	1614.83	1614.84	1636.81	1636.81	1652.78	1652.80	E B	25.9
**YKL**								
Oxo Test	1470.88	1470.92	1492.86	1492.96	1508.84	1508.91	M	12.7
Oxo Unfolded	1470.88	1470.95	1492.86	1492.94	1508.84	1508.90	M	11.5
Glu$^{S}_{4}$ Test	1486.86	1486.89	1508.84	1508.86	1524.81	1524.98	M	16.8
Glu$^{S}_{4}$ Unfolded	1486.86	1486.92	1508.84	1508.90	1524.81		M	13.6
Lys$^{S}_{9}$ Test	1486.86	1486.90	1508.84	1509.02	1524.81		M	22.2
Lys$^{S}_{9}$ Unfolded	1486.86	1486.89	1508.84	1508.97	1524.81	1524.95	M	21.1
Leu$^{S}_{11}$-OH Test	1487.84	1487.95	1509.82	1509.91	1525.80	1525.99	M	16.9
Leu$^{S}_{11}$-OH Unfolded	1487.84	1487.93	1509.82	1509.89	1525.80		M	15.6

*Gradient K used for all analytical HPLC injections of HPT library and Gradient M used for YKL library.

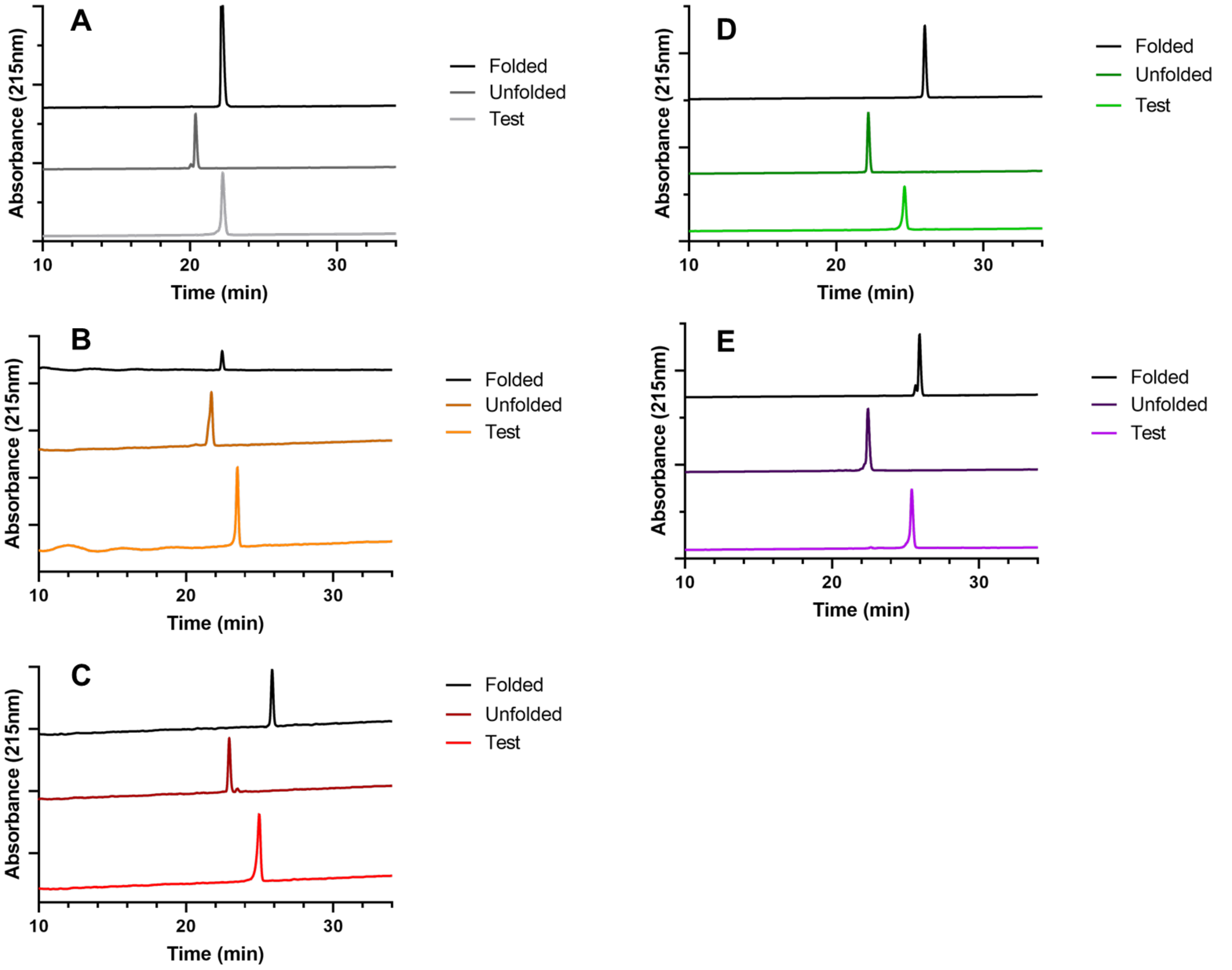

Figure S1. Analytical HPLC (gradient **K**) of synthesized HPT β-hairpins: HPT (**A**), HPT-Thr$^{S}_{2}$ (**B**), HPT-Val$^{S}_{3}$ (**C**), HPT-Ile$^{S}_{10}$ (**D**), and HPT-Leu$^{S}_{11}$-OH.

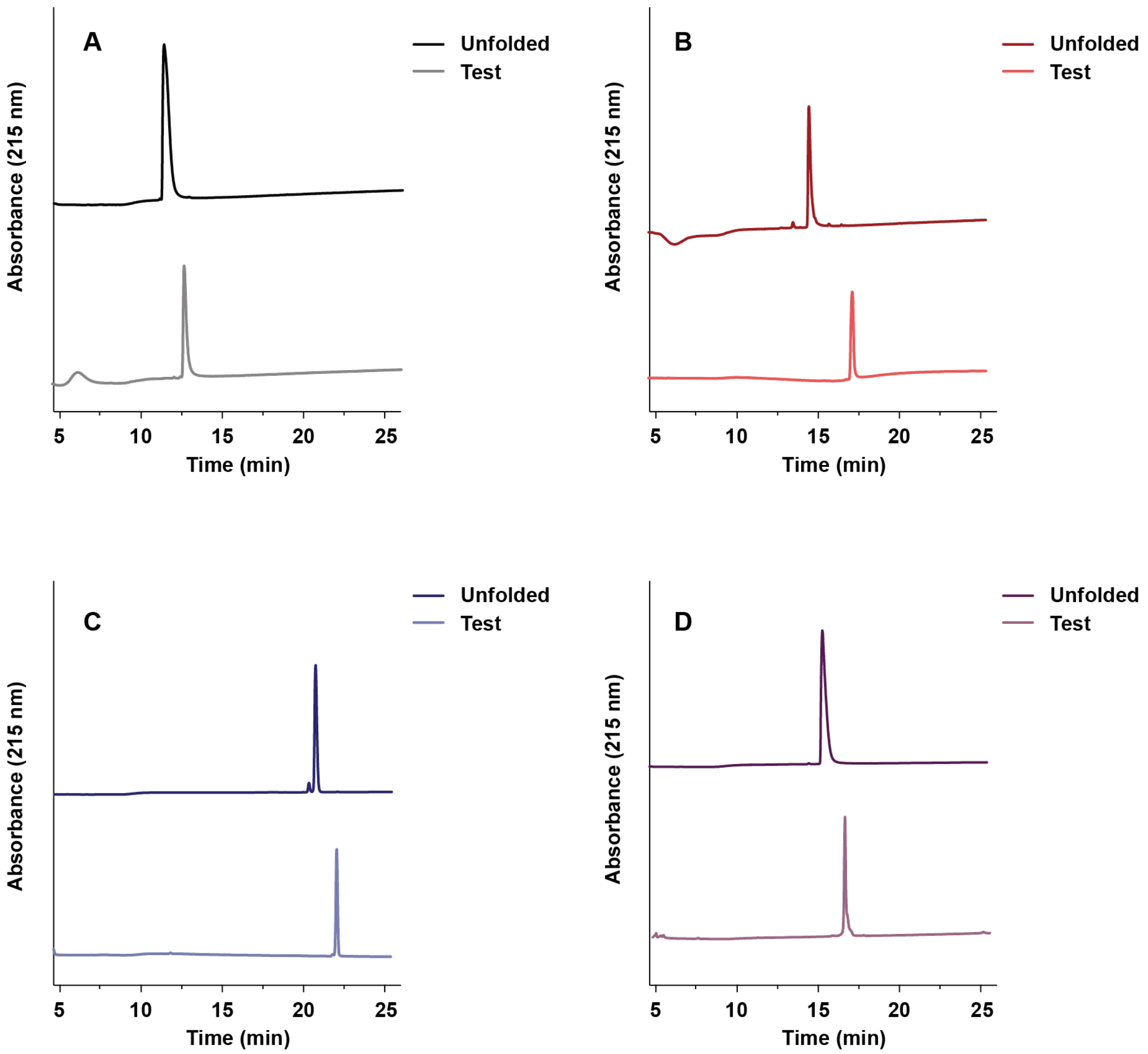

Figure S2. Analytical HPLC (gradient **M**) of synthesized YKL β-hairpins: YKL (**A**), YKL-Glu$^{S}{}_{4}$ (**B**), YKL-Lys$^{S}{}_{9}$ (**C**), and YKL-Leu$^{S}{}_{11}$-OH (**D**).

Table S2. HPLC gradients for purification (solvent A = MilliQ water with 0.1% TFA, solvent B = acetonitrile with 0.1% TFA)

No.	Time/CV	% B	No.	Time	%B
**A**	2 CV	5	**B**	0.00	2
	30.9 CV	5-50		3.00	5
	3 CV	50		8.00	20
	5 CV	100		10.00	20
	1 CV	50		30.00	25
				31.00	100
				36.00	100
				38.00	5
**C**	0.00	5	**D**	0.00	5
	3.00	5		3.00	5
	8.00	20		8.00	22
	10.00	20		10.00	22
	30.00	30		30.00	27
	31.00	100		31.00	100
	41.00	100		36.00	100
	43.00	5		38.00	5
**E**	0.00	5	**F**	0.00	5
	3.00	5		3.00	5
	10.00	20		8.00	15
	15.00	20		11.00	15
	35.00	40		41.00	30
	40.00	40		43.00	100
	45.00	100		53.00	100
	55.00	100		55.00	5
	58.00	5			
**G**	0.00	5	**H**	0.00	5
	3.00	5		3.00	5
	10.00	20		8.00	10
	15.00	20		11.00	10
	35.00	25		51.00	50
	40.00	30		55.00	100
	45.00	100		60.00	100
	55.00	100		62.00	5
	58.00	5			
**I**	0.00	5	**J**	0.00	5
	3.00	5		3.00	5
	10.00	25		10.00	20
	15.00	25		15.00	20
	35.00	30		35.00	30
	40.00	30		40.00	40
	45.00	100		45.00	100
	55.00	100		55.00	100
	58.00	5		58.00	5
**K**	0.00	5	**L**	0.00	5
	5.00	5		3.00	5
	50.00	50		10.00	15
	53.00	100		15.00	15
	58.00	100		35.00	20
	60.00	5		40.00	100
				45.00	100
				50.00	5
**M**	0.00	10			
	3.00	10			
	23.00	50			
	28.00	100			
	33.00	100			
	38.00	10			

Circular Dichroism

HPT

Sample Preparation. Dried peptide was dissolved in 50:50 v/v MilliQ water/ acetonitrile and quantified by UV based on the thioamide extinction coefficient (ε_{274nm} = 10,169 $M^{-1}cm^{-1}$). Aliquots of Test, Folded, and Unfolded versions of all HPT peptides were prepared and lyophilized. The aliquot was dissolved in 50 mM sodium acetate pH 5.5 (HPT-Thr^S_2 and HPT-Val^S_3) and 50 mM NaH_2PO_4 pH 5.5 (HPT-Ile^S_{10} and HPT-Leu^S_{11}-OH) to 130-150 µM. The final concentration was measured based on UV. The HPT peptide was aliquoted based on mass and was dissolved in 50 mM sodium acetate pH 5.5 to 1 mM. This stock was diluted to 150 µM.

Data Measurement. The wavelength absorbance scans were collected on a Jasco J-1500 CD spectrometer with a 1 mm path length Helma 110-QS CD cuvette. Measurements were completed at 25 ˚C, collecting between 350-190 nm with a continuous scanning rate of 50 nm/min (450 FL λ, 1 nm bandwidth and 1.0 nm data pitch) and a digital integration time (DIT) of 1 second. The instrument was blanked with the corresponding buffer prior to sample collection. The raw signal (θ, mDeg) was converted to molar ellipticity (θ_{Molar}) (**Eq. S1**), where *l* is the pathlength in cm and c is the concentration (M).

$$\theta_{Molar} = \frac{\theta}{10 * l * c} \qquad \textbf{(Eq. S1)}$$

The scans were smoothed with GraphPad Prism 7.01 software (San Diego, Ca) by averaging 10 neighbors on each side, and using a fourth order smoothing polynomial.

YKL

The Far-UV CD spectra of YKL and its thioamidated analogs were collected on a JASCO-715 spectropolarimeter with 1 mm path-length Starna 21-Q-1 CD cuvette. The data were recorded at 100 µM concentration in 100 mM sodium acetate buffer (pH 3.8). Scans were carried out at 20 °C over the range of 190-260 nm with 0.5 nm increments and a 2 nm bandwidth. Thermal unfolding experiments were monitored at 215 nm over a temperature range of 10-90 °C. The samples were equilibrated for 5 minutes at each temperature.

Nuclear Magnetic Resonance Spectroscopy

HPT

Sample Preparation. Dried peptide was dissolved in 50:50 v/v MilliQ water/ acetonitrile and quantified by UV based on the thioamide extinction coefficient (ε_{274nm} = 10,169 $M^{-1}cm^{-1}$). The material was aliquoted and lyophilized. The UV measurement was compared against the mass of the dried peptide. NMR samples were prepared by dissolving the lyophilized, dried peptide (≥ 95% purity) in 0.5-0.6 mL of 50 mM sodium deuterioacetate buffer pH 5.5 (9:1 v/v MilliQ H_2O/ D_2O) (HPT-Thr$^S{}_2$ and HPT-Val$^S{}_3$) or 50 mM NaH_2PO_4 pH 5.5 (9:1 v/v MilliQ H_2O/ D_2O) (HPT-Ile$^s{}_{10}$ and HPT-Leu$^S{}_{11}$-OH) to 1-10 mM concentration (see exact concentration in **Table S3**). 2-Dimethyl-2-silapentane-5-sulfonate (DSS) was added to each NMR sample as the internal reference. (DSS was added from a 1 mg/mL stock in sterile Milli-Q water to ~30 μM final concentration.)

Data Collection. The HPT data was previously collected and published on by the Cheng lab,[1] however the raw data was processed and analyzed again by KEF to ensure that the analysis was consistent. NTU: NMR data were collected on a Bruker AVIII 800 MHz spectrometer. Total correlation spectroscopy (TOCSY)[8] and rotating-frame nuclear Overhauser effect spectroscopy (ROESY)[9] experiments were performed by collecting 2048 points in $f\,2$ with 4-8 scans and 256-512 points in $f\,1$ at 298 K. Water suppression was achieved with the WATERGATE solvent suppression sequence.[10] TOCSY and ROESY had a spin locking field of 10 kHz. Mixing times of 60 and 200 ms were used for the TOCSY and ROESY respectively. Penn: NMR data was collected on a Bruker AVANCE NEO 600 MHz spectrometer. TOCSY and ROESY were performed by collecting 4096 points in $f\,2$ with 16-32 scans and 512 points in $f\,1$ at 298 K. Water suppression was achieved with the WATERGATE solvent suppression sequence.[10] Mixing times of 80 and 250 ms were used for the TOCSY and ROESY respectively.

YKL

Sample Preparation. NMR samples of YKL and its thioamidated alalogs were prepared by dissolving the lyophilized, dried peptide (≥ 95% purity) in 0.5-0.6 mL of 100 mM sodium deuteroacetate buffer pH 3.8 (9:1 v/v MilliQ H_2O/ D_2O) to obtain 1-3 mM concentration (see exact concentration in **Table S3**). Trimethylsilylpropanoic acid (TMSP) was added to each NMR sample as the internal reference.

Data Collection. IIS: NMR data for YKL and its thioamidated analogs were collected on a Bruker Avance 700 MHz spectrometer. Standard Bruker pulse sequences zgesgp for 1H, mlevesgpph/dipsi2rcesgpph (60 ms mixing time) for TOCSY, roesyesgpph (100 ms mixing time) for ROESY were used to acquire the NMR data. Two-dimensional data were obtained using 2048 data points in the direct dimension ($f\,2$) and 512 data points in the indirect dimension ($f\,1$).

Table S3. Concentration of NMR sample

Peptide	Collection	Concentration (mM)
**HPT**		
Thr^{S}_{2} Test	NTU	7.9
Thr^{S}_{2} Unfolded	NTU	5.9
Thr^{S}_{2} Folded	NTU	1.1
Val^{S}_{3} Test	NTU	6.8
Val^{S}_{3} Unfolded	NTU	3.7
Val^{S}_{3} Folded	NTU	2.7
Ile^{S}_{10} Test	Penn	3.8
Ile^{S}_{10} Unfolded	Penn	4.2
Ile^{S}_{10} Folded	Penn	7.7
Leu^{S}_{11}-OH Test	Penn	0.8
Leu^{S}_{11}-OH Unfolded	Penn	3.0
Leu^{s}_{11}-OH Folded	Penn	1.8
**YKL**		
Oxo Test	IIS	2.5
Oxo Unfolded	IIS	2.8
Glu^{S}_{4} Test	IIS	1.9
Glu^{S}_{4} Unfolded	IIS	2.4
Lys^{S}_{9} Test	IIS	3.1
Lys^{S}_{9} Unfolded	IIS	2.4
Leu^{S}_{11}-OH Test	IIS	2.7
Leu^{S}_{11}-OH Unfolded	IIS	3.0

Data Processing. All 2D ^{1}H-^{1}H NMR were processed and assigned with MestReNova 14.1.0 (Santiago de Compostela, Spain). Apodization of Sine Square 90˚ was used for both $f\,2$ and $f\,1$, and zero-fill was 2x the size of the FID. A baseline correction of a Berstein polynomial fit of order 3 was used. The spectra were referenced based on the DSS or TMSP signal. The spectra were phased manually or automatically. Peaks were manually picked with standard MestReNova settings. The TOCSY was assigned with the ROESY. NOE connectivities were determined from the ROESY with an intensity cutoff of 0.3 for the NTU and IIS collected data and 300 for the Penn collected data (**Fig. S15**). Chemical shift assignments for all HPT and YKL peptides analyzed in the main text are given in **Table S5**.

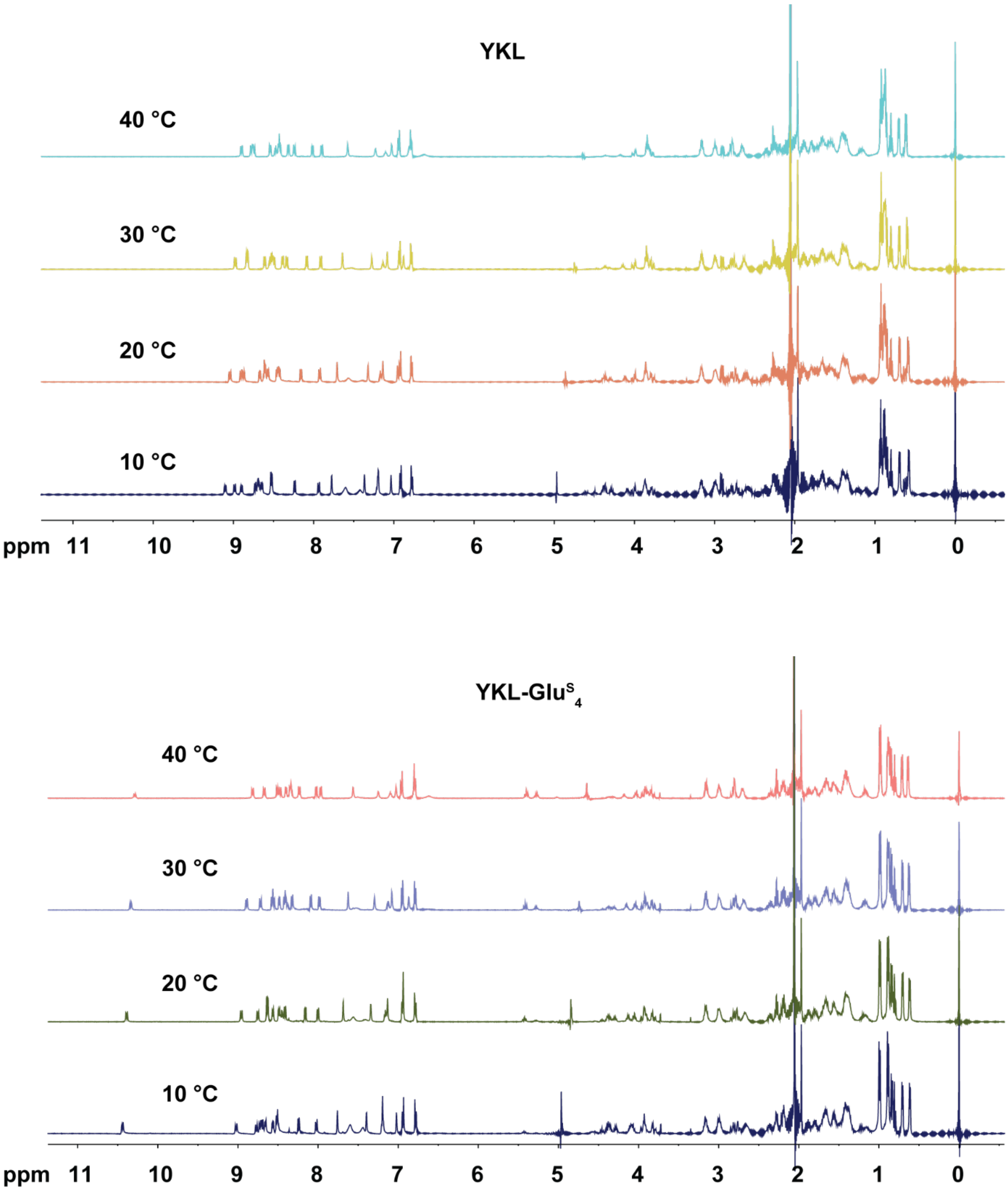

Figure S3a. ^{1}H NMR overlay of thioamide-containing Test YKL β-hairpins acquired at different temperatures.

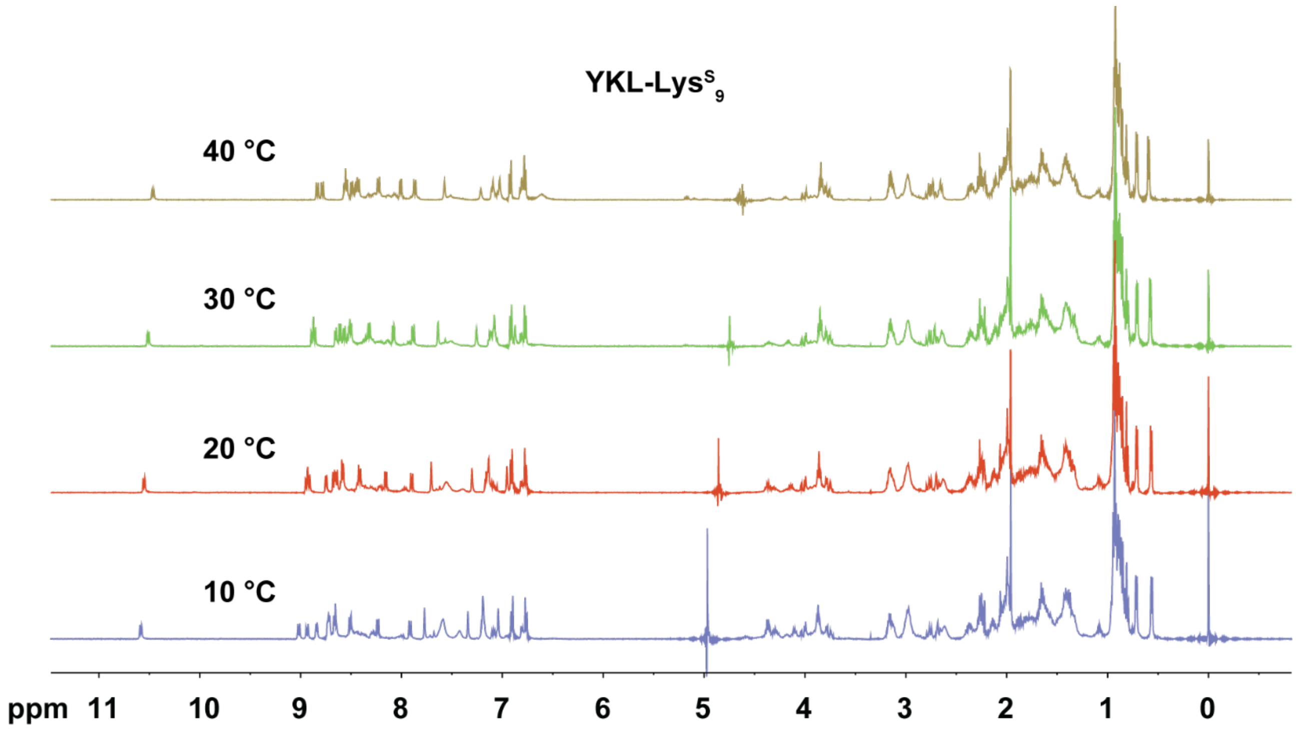

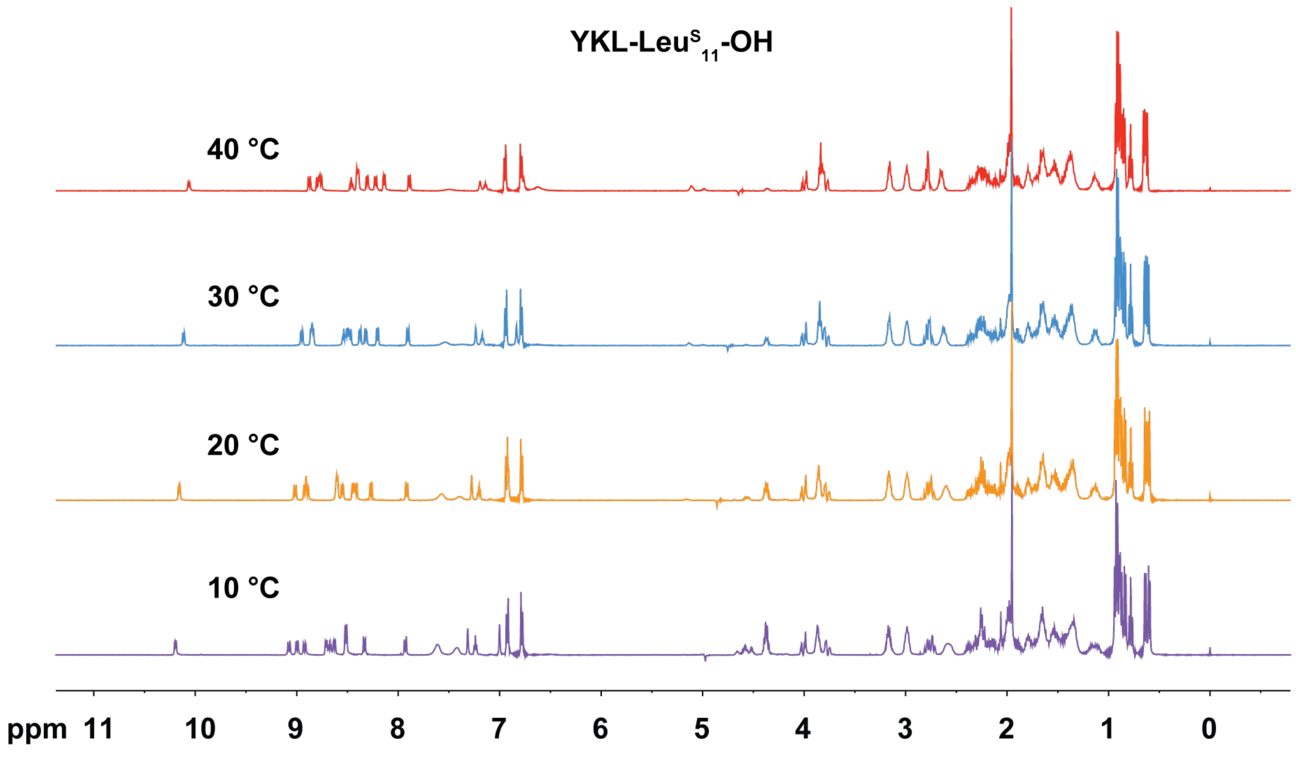

Figure S3b. ^{1}H NMR overlay of thioamide-containing Test YKL β-hairpins acquired at different temperatures.

Table S4. Amide temperature coefficient (in ppb/K) measurements for YKL and its thioamidated analogs

Residue	YKL	YKL-Glu^{S}$_{4}$	YKL-Lys^{S}$_{9}$	YKL-Leu^{S}$_{11}$-OH
*Arg$_1$	-7.3	-7.3	-7.3	-6.5
Tyr$_2$	-7	-9.3	-9.3	-7
*Val$_3$	-5	-4	-4	-5
Glu$_4$	-7.5	-7.3	-8	-8
*Val$_5$	-8	-5.3	-7.8	-7.8
pro$_6$				
Gly$_7$	-7.3	-6.3	-7.3	-7
*Lys$_8$	-1.3	-2	-1.5	-1.3
Lys$_9$	-9.5	-6.3	-7.3	-9.8
*Ile$_{10}$	-7.3	-7.5	-4.3	-7
Leu$_{11}$	-7.3	-8.5	-9.3	-9.8
*Gln$_{12}$	-6.5	-7.5	-8.8	-4.8

*Hydrogen bonded amide protons $\Delta\delta/\Delta T$.

Table S5. ^{1}H Chemical shift assignments for HPT and YKL β-hairpins

YKL Test

Residue	NH	Hα	Hβ	Others
Ac		1.955		
Arg_1	8.250	4.369	1.653	Hγ: 1.496, Hδ: 3.157, HNt: 7.209
Tyr_2	8.548	5.121	2.727	2,6H: 6.912, 3,5H: 6.772
Val_3	8.902	4.379	2.004	Hγ: 0.861
Glu_4	8.660	4.994	1.975, 1.877	Hγ: 2.229
Val_5	9.000	4.593	1.985	Hγ: 0.930
pro_6		4.359	2.376, 2.024	Hγ: 2.112, Hδ: 3.870
Gly_7	8.693	4.007, 3.763		
Lys_8	7.937	4.574	1.789	Hγ: 1.389, Hδ: 1.672, Hε: 2.991, HNt: 7.612
Lys_9	8.542	4.613	1.643, 1.526	Hγ: 1.105, Hδ: 1.330, Hε: 2.581, HNt: 7.427
Ile_{10}	9.114	4.486	1.907	Hγ: 1.399, 1.223, Hδ or Hγ (Me): 0.881
Leu_{11}	8.712	4.076	1.584	Hγ: 1.399, Hδ: 0.685, 0.568
Gln_{12}	8.740	4.281	2.034, 1.848	Hγ: 2.268, HNt: 7.375, 7.051
NH_2	7.794, 7.199			

YKL Unfolded

Residue	NH	Hα	Hβ	Others
Ac		1.998		
Arg_1	8.292	4.181	1.637	Hγ: 1.457, Hδ: 3.124, HNt: 7.179
Tyr_2	8.382	4.619	2.968	2,6H: 7.092, 3,5H: 6.803
Val_3	8.020	4.001	1.927	Hγ: 0.870
Glu_4	8.481	4.290	1.981	Hγ: 2.373
Val_5	8.446	4.396	2.068	Hγ: 0.952
Pro_6		4.392	2.318	Hγ: 2.005, 1.989, Hδ: 3.876, 3.699
Gly_7	8.598	3.938		
Lys_8	8.209	4.306	1.754	Hγ: 1.402, Hε: 2.974, HNt: 7.603
Lys_9	8.481	4.306	1.731	Hγ: 1.410, Hε: 2.974, HNt: 7.603
Ile_{10}	8.404	4.126	1.833	Hγ: 1.481, 1.191, Hδ or Hγ (Me): 0.878
Leu_{11}	8.506	4.384	1.614	Hδ: 0.859
Gln_{12}	8.481	4.282	1.981	Hγ: 2.373, HNt: 7.630, 6.960
NH_2	7.700, 7.204			

YKL-Glu$^S_4$ Test

Residue	NH	Hα	Hβ	Others
Ac		1.953		
Arg_1	8.261	4.337	1.650	Hγ: 1.484, Hδ: 3.145, HNt: 7.204
Tyr_2	8.536	5.050	2.815, 2.757	2,6H: 6.940, 3,5H: 6.783
Val_3	8.774	4.357	2.022	Hγ: 0.849
Glu^S_4	8.746	5.256	2.061, 1.992	Hγ: 2.178
Val_5	10.460	5.436	2.178	Hγ: 0.976
pro_6		4.398	2.354, 1.992	Hγ: 2.080, 2.061, Hδ: 4.072, 3.925
Gly_7	8.521	3.937, 3.790		
Lys_8	8.026	4.533	1.777	Hγ: 1.377, Hδ: 1.660, Hε: 2.979, HNt: 7.611
Lys_9	8.584	4.562	1.641, 1.533	Hγ: 1.123, Hδ: 1.357, Hε: 2.637, HNt: 7.452
Ile_{10}	9.042	4.445	1.875	Hγ: 1.396, 1.201, Hδ or Hγ (Me): 0.869
Leu_{11}	8.680	4.083	1.562	Hγ: 1.406, Hδ: 0.693, 0.605
Gln_{12}	8.711	4.259	2.031, 1.846	Hγ: 2.266, HNt: 7.410, 7.040
NH_2				

YKL-Glu$^S{}_4$ Unfolded

Residue	NH	Hα	Hβ	Others
Ac		1.982		
Arg_1	8.306	4.181	1.630	Hγ: 1.464, Hδ: 3.125, HNt: 7.190
Tyr_2	8.402	4.640	3.037, 2.930	2,6H: 7.102, 3,5H: 6.810
Val_3	8.030	4.034	1.992	Hγ: 0.868
$Glu^S{}_4$	8.568	4.659	2.021	Hγ: 2.363, 2.305
Val_5	10.207	4.757	2.217	Hγ: 1.054
Pro_6		4.239	2.314, 1.943	Hγ: 2.070, Hδ: 3.678
Gly_7	8.619	3.946		
Lys_8	8.209	4.298	1.777	Hγ: 1.396, Hε: 2.979, HNt: 7.626
Lys_9	8.457	4.288	1.757	Hγ: 1.406, Hε: 2.979, HNt: 7.626
Ile_{10}	8.427	4.341	1.611	Hδ or Hγ (Me): 0.926
Leu_{11}	8.404	4.356	1.650	Hδ: 0.868
Gln_{12}	8.428	4.288	2.109, 1.982	Hγ: 2.353, HNt: 7.653, 6.968
NH_2	7.688, 7.219			

YKL-Lys$^S{}_9$ Test

Residue	NH	Hα	Hβ	Others
Ac		1.958		
Arg_1	8.251	4.361	1.655	Hγ: 1.489, Hδ: 3.150, HNt: 7.201
Tyr_2	8.532	5.143	2.759, 2.671	2,6H: 6.902, 3,5H: 6.763
Val_3	8.929	4.371	1.987	Hγ: 0.873
Glu_4	8.736	4.996	2.007, 1.860	Hγ: 2.241
Val_5	9.032	4.596	1.987	Hγ: 0.932
pro_6		4.373	2.378, 1.985	Hγ: 2.125, 2.058, Hδ: 3.866
Gly_7	8.675	4.019, 3.756		
Lys_8	7.917	4.576	1.762	Hγ: 1.391, Hε: 2.974, HNt: 7.611
$Lys^S{}_9$	8.684	4.879	1.792, 1.557	Hγ: 1.079, Hδ: 1.333, Hε: 2.622, HNt: 7.447
Ile_{10}	10.595	5.192	2.143	Hγ: 1.432, 1.378, Hδ or Hγ (Me): 0.912
Leu_{11}	8.864	4.098	1.606	Hγ: 1.411, Hδ: 0.717, 0.561
Gln_{12}	8.739	4.293	2.036, 1.850	Hγ: 2.271, HNt: 7.344, 7.060
NH_2	7.787, 7.197			

YKL-Lys$^S{}_9$ Unfolded

Residue	NH	Hα	Hβ	Others
Ac		1.992		
Arg_1	8.294	4.182	1.639	Hγ: 1.467, Hδ: 3.132, HNt: 7.191
Tyr_2	8.390	4.613	2.994, 2.945	2,6H: 7.092, 3,5H: 6.805
Val_3	8.010	3.995	1.928	Hγ: 0.864
Glu_4	8.480	4.274	1.966	Hγ: 2.351
Val_5	8.452	4.396	2.054	Hγ: 0.958
Pro_6		4.396	2.312	Hγ: 2.007, 1.975, Hδ: 3.882, 3.692
Gly_7	8.605	3.940		
Lys_8	8.178	4.300	1.764	Hγ: 1.412, Hε: 2.968, HNt: 7.601
$Lys^S{}_9$	8.599	4.660	1.787	Hγ: 1.373, Hε: 2.968, HNt: 7.601
Ile_{10}	10.125	4.660	2.014	Hγ: 1.537, 1.271, Hδ or Hγ (Me): 0.934
Leu_{11}	8.649	4.378	1.678	Hγ: 1.553
Gln_{12}	8.452	4.284	1.975	Hγ: 2.363, HNt: 7.634, 6.963
NH_2	7.712, 7.205			

YKL-Leu^S$_{11}$-OH Test

Residue	NH	Hα	Hβ	Others
Ac		1.952		
Arg_1	8.34	4.385	1.727, 1.620	Hγ: 1.483, Hδ: 3.163, HNt: 7.243
Tyr_2	8.532	5.176	2.778, 2.730	2,6H: 6.918, 3,5H: 6.777
Val_3	8.921	4.375	1.981	Hγ: 0.857
Glu_4	8.643	5.020	1.981, 1.883	Hγ: 2.255
Val_5	9.008	4.600	1.961	Hγ: 0.926
pro_6		4.360	2.382, 2.001	Hγ: 2.108, Hδ: 3.867
Gly_7	8.683	4.004, 3.759		
Lys_8	7.927	4.580	1.786	Hγ: 1.385, Hδ: 1.668, Hε: 2.987, HNt: 7.619
Lys_9	8.526	4.648	1.629, 1.541	Hγ: 1.102, Hδ: 1.326, Hε: 2.577, HNt: 7.426
Ile_{10}	9.083	4.570	2.001	Hγ: 1.375, 1.170, Hδ or Hγ (Me): 0.897
Leu^S_{11}	8.730	4.502	1.659	Hγ: 1.512, Hδ: 0.613
Gln_{12}	10.199	4.648	1.942	Hγ: 2.225, HNt: 7.318, 7.016
OH				

YKL-Leu^S$_{11}$-OH Unfolded

Residue	NH	Hα	Hβ	Others
Ac		1.991		
Arg_1	8.294	4.173	1.630	Hγ: 1.458, Hδ: 3.125, HNt:
Tyr_2	8.369	4.627	3.006, 2.926	Hγ:
Val_3	8.026	4.001	1.931	Hγ: 0.871
Glu_4	8.481	4.292	1.982	Hγ: 2.408
Val_5	8.434	4.400	2.068	Hγ: 0.949
Pro_6		4.387	2.311, 1.982	Hγ: 2.013, Hδ: 3.875, 3.692
Gly_7	8.604	3.939		
Lys_8	8.192	4.322	1.755	Hγ: 1.395, Hε: 2.975, HNt: 7.605
Lys_9	8.502	4.330	1.739	Hγ: 1.426, Hε: 2.975, HNt: 7.605
Ile_{10}	8.381	4.173	1.888	Hγ: 1.172, Hδ or Hγ (Me): 0.886
Leu^S_{11}	8.472	4.760	1.737, 1.638	Hδ: 0.922, 0.874
Gln_{12}	9.989	4.713	2.092	Hγ: 2.350, HNt: 7.610, 6.913
OH				

HPT Test

Residue	NH	Hα	Hβ	Others
Ac		2.019		
Arg_1	8.296	4.388	1.827, 1.749	Hγ: 1.630, Hδ: 3.202, HNt: 7.205
Thr_2	8.307	4.603	4.084	Hγ: 1.138
Val_3	8.478	4.260	2.048	Hγ: 0.893
Glu_4	8.437	4.711	1.910, 1.832	Hγ: 2.160, 2.062
Val_5	8.604	4.544	1.999	Hγ: 0.927
pro_6		4.388	2.342, 1.988	Hγ: 2.087, 2.048, Hδ: 3.876, 3.819
Gly_7	8.511	3.986, 3.800		
Orn_8	7.993	4.505	1.851, 1.798	Hγ: 1.692, Hδ: 3.016, HNt: 7.614
Lys_9	8.469	4.485	1.725	Hγ: 1.314, Hδ: 1.451, Hε: 2.967
Ile_{10}	8.589	4.260	1.847	Hγ: 1.406, 1.171, Hδ or Hγ (Me): 0.868, 0.805
Leu_{11}	8.400	4.456	1.597	Hδ: 0.902, 0.849
Gln_{12}	8.487	4.319	2.078, 1.941	Hγ: 2.322, HNt: 7.448, 6.878
NH_2	7.648, 7.126			

HPT Unfolded

Residue	NH	Hα	Hβ	Others
Ac		2.030		
Arg_1	8.304	4.355	1.831, 1.748	Hγ: 1.636, Hδ: 3.206, HNt: 7.215
Thr_2	8.297	4.375	4.174	Hγ: 1.186
Val_3	8.202	4.130	2.056	Hγ: 0.921
Glu_4	8.467	4.341	1.973, 1.900	Hγ: 2.272, 2.205
Val_5	8.369	4.414	2.071	Hγ: 0.958
Pro_6		4.410	2.316, 1.934	Hγ: 2.065, 1.999 Hδ: 3.882, 3.706
Gly_7	8.492	3.949		
Orn_8	8.188	4.346	1.870, 1.773	Hγ: 1.702, Hδ: 3.010, HNt: 7.623
Lys_9	8.371	4.311	1.782, 1.729	Hγ: 1.371, Hδ: 1.440, Hε: 2.991, HNt: 7.552
Ile_{10}	8.276	4.135	1.836	Hγ: 1.484, 1.186, Hδ or Hγ (Me): 0.883
Leu_{11}	8.381	4.390	1.655, 1.582	Hδ: 0.865
Gln_{12}	8.340	4.302	2.105, 1.978	Hγ: 2.364, HNt: 7.534, 6.870
NH_2	7.599, 7.112			

HPT Folded

Residue	NH	Hα	Hβ	Others
Ac		2.080		
Cys_N	8.448	5.211	3.168, 2.655	
Arg_1	8.778	4.672	1.833	Hγ: 1.686, 1.544, Hδ: 3.183, HNt: 7.130
Thr_2	8.615	4.976	3.928	Hγ: 1.079
Val_3	9.093	4.467	1.999	Hγ: 0.854, 0.803
Glu_4	8.481	5.054	1.943, 1.803	Hγ: 2.072
Val_5	8.847	4.623	1.945	Hγ: 0.903
pro_6		4.337	2.390, 1.979	Hγ: 2.141, 2.058, Hδ: 3.867
Gly_7	8.721	4.065, 3.723		
Orn_8	7.890	4.653	1.818	Hγ: 1.681, Hδ: 3.007, HNt: 7.609
Lys_9	8.519	4.760	1.740	Hγ: 1.520, 1.219, Hδ: 1.451, Hε: 2.928
Ile_{10}	9.143	4.467	1.852	Hγ: 1.336, 1.123, Hδ or Hγ (Me): 0.847, 0.781
Leu_{11}	8.403	4.741	1.652	Hγ: 1.490, Hδ: 0.796
Gln_{12}	9.231	4.663	2.072, 1.882	Hγ: 2.268, 2.209, HNt: 7.328, 6.833
Cys_C	8.991	5.074	3.134, 3.007	
NH_2	7.614, 7.247			

HPT-Thr$^S_2$ Test

Residue	NH	Hα	Hβ	Others
Ac		2.032		
Arg_1	8.284	4.392	1.844, 1.744	Hγ: 1.633, Hδ: 3.194, HNt: 7.186
Thr^S_2	8.463	4.832	4.081	Hγ: 1.116
Val_3	10.206	4.820	2.196	Hγ: 0.959
Glu_4	8.527,	4.756	1.841	Hγ: 2.214, 2.067
Val_5	8.592	4.556	1.992	Hγ: 0.934
pro_6		4.386	2.355, 1.991	Hγ: 2.055, 2.096, Hδ: 3.875, 3.828,
Gly_7	8.533	3.998, 3.799		
Orn_8	7.980	4.521	1.809	Hγ: 1.697, Hδ: 3.018, HNt: 7.618
Lys_9	8.484	4.497,	1.727	Hγ: 1.304, Hδ: 1.457, Hε: 2.965
Ile_{10}	8.596	4.268	1.851	Hγ: 1.404, 1.175, Hδ or Hγ (Me): 0.869
Leu_{11}	8.396	4.468	1.593	Hδ: 0.899, 0.852
Gln_{12}	8.512	4.315	2.073, 1.938	Hγ: 2.325, HNt: 7.456, 6.873
NH_2	7.660, 7.133			

HPT-Thr$^S_2$ Unfolded

Residue	NH	Hα	Hβ	Others
Ac		2.036		
Arg_1	8.287	4.345	1.843, 1.743	Hγ: 1.632, Hδ: 3.192, HNt: 7.198
Thr^S_2	8.471	4.620	4.145	Hγ: 1.163
Val_3	9.997	4.579	2.200	Hγ: 0.987
Glu_4	8.600	4.345	1.972, 1.901	Hγ: 2.314, 2.230
Val_5	8.335	4.409	2.071	Hγ: 0.958
Pro_6		4.408	2.317, 1.937	Hγ: 2.065, 2.001, Hδ: 3.880, 3.705
Gly_7	8.501	3.946		
Orn_8	8.187	4.350	1.872, 1.773	Hγ: 1.702, Hδ: 3.010, HNt: 7.630
Lys_9	8.376	4.309	1.732	Hγ: 1.374, Hδ: 1.439, Hε: 2.987, HNt: 7.555
Ile_{10}	8.288	4.134	1.843	Hγ: 1.48, 1.193, Hδ or Hγ (Me): 0.888
Leu_{11}	8.389	4.386	1.655, 1.585	Hδ: 0.935, 0.864
Gln_{12}	8.346	4.298	2.101, 1.978	Hγ: 2.364, HNt: 7.538, 6.874
NH_2	7.603, 7.115			

HPT-Thr$^S_2$ Folded

Residue	NH	Hα	Hβ	Others
Ac		2.036		
Cys_N	8.455	5.260	3.172, 2.651	
Arg_1	8.783	4.708	1.852	Hγ: 1.693, 1.509, Hδ: 3.172, HNt: 7.113
Thr^S_2	8.731	5.209	4.016	Hγ: 1.033
Val_3	10.630	5.279		Hγ: 0.906
Glu_4	8.651	5.095	1.934, 1.814	Hγ: 2.080
Val_5	8.828	4.650	1.960	Hγ: 0.913
pro_6		4.345	2.398, 1.985	Hγ: 2.150, 2.068, Hδ: 3.874
Gly_7	8.744	4.079, 3.737		
Orn_8	7.914	4.663	1.833	Hγ: 1.693, Hδ: 3.013
Lys_9	8.535	4.771	1.744	Hγ: 1.578, 1.217, Hδ: 1.490, Hε: 2.924
Ile_{10}	9.176	4.460	1.846	Hγ: 1.354, 1.128, Hδ or Hγ (Me): 0.856
Leu_{11}	8.472	4.733	1.674	Hγ: 1.481, Hδ: 0.798
Gln_{12}	9.264	4.670	2.080, 1.871	Hγ: 2.280, 2.220, HNt: 7.353, 6.852
Cys_C	9.015	5.101	3.127, 3.013	
NH_2	7.595, 7.247			

HPT-Val$^S_3$ Test

Residue	NH	Hα	Hβ	Others
Ac		2.035		
Arg_1	8.313	4.384	1.859, 1.754	Hγ: 1.648, Hδ: 3.207, HNt: 8.216
Thr_2	8.315	4.395	4.208	Hγ: 1.185
Val^S_3	8.283	4.414	2.105	Hγ: 0.951, 0.887
Glu_4	10.301	5.035	2.158, 2.023	Hγ: 2.252
Val_5	8.571	4.519	2.035	Hγ: 0.957
pro_6		4.410	2.328, 1.994	Hγ: 2.076, 2.052, Hδ: 3.892, 3.796
Gly_7	8.413	3.962, 3.845		
Orn_8	8.033	4.449	1.812	Hγ: 1.710, Hδ: 3.019, HNt: 7.616
Lys_9	8.461	4.390	1.719	Hγ: 1.355, Hδ: 1.449, Hε: 2.978
Ile_{10}	7.998	4.173	1.847	Hγ: 1.449, 1.185, Hδ or Hγ (Me): 0.887
Leu_{11}	8.367	4.390	1.648	Hγ: 1.584, Hδ: 0.928, 0.863
Gln_{12}	8.349	4.302	2.105, 1.976	Hγ: 2.357, HNt: 7.528, 6.876
NH_2	7.605, 7.112			

HPT-Val $^{S}{}_{3}$ Unfolded

Residue	NH	Hα	Hβ	Others
Ac		2.032		
Arg_1	8.308	4.370	1.857, 1.750	Hγ: 1.648, Hδ: 3.209, HNt: 7.220
Thr_2	8.308	4.362	4.212	Hγ: 1.185
Val^{S}_{3}	8.282	4.377	2.086	Hγ: 0.950, 0.887
Glu_4	10.299	4.859	2.308	Hγ: 2.232, 2.080
Val_5	8.480	4.421	2.073	Hγ: 0.969
Pro_6		4.404	2.314, 1.934	Hγ: 2.067, 1.991, Hδ: 3.888, 3.701
Gly_7	8.504	3.952		
Orn_8	8.187	4.351	1.870	Hγ: 1.775, 1.670, Hδ: 3.012,
Lys_9	8.371	4.313	1.737, 1.675	Hγ: 1.375, Hδ: 1.439, Hε: 2.987, HNt: 7.558
Ile_{10}	8.282	4.136	1.838	Hγ: 1.483, 1.185, Hδ or Hγ (Me): 0.880
Leu_{11}	8.385	4.383	1.654	Hγ: 1.585, Hδ: 0.931, 0.861
Gln_{12}	8.340	4.301	2.105, 1.978	Hγ: 2.359, HNt: 7.534, 6.870
NH_2	7.600, 7.112			

HPT-Val $^{S}{}_{3}$ Folded

Residue	NH	Hα	Hβ	Others
Ac		2.053		
Cys_N	8.431	4.953	3.132, 2.875	
Arg_1	8.687	4.604	1.895, 1.806	Hγ: 1.634, Hδ: 3.196, HNt: 7.166
Thr_2	8.522	4.680	4.084	Hγ: 1.089
Val^{S}_{3}	8.279	4.528	2.104	Hγ: 0.905, 0.873
Glu_4	10.580	5.214	2.079	Hγ: 2.161
Val_5	8.515	4.639	2.015	Hγ: 0.955
pro_6		4.363	2.371, 1.990	Hγ: 2.117, 2.047, Hδ: 3.844
Gly_7	8.675	4.014, 3.811		
Orn_8	7.972	4.560	1.869, 1.736	Hγ: 1.685, Hδ: 3.005
Lys_9	8.563	4.395	1.717, 1.634	Hγ: 1.235, Hδ: 1.412, Hε: 2.945
Ile_{10}	7.607	4.287	1.945	Hγ: 1.431, 1.133, Hδ or Hγ (Me): 0.905
Leu_{11}	8.367	4.471	1.622	Hγ: 1.577, Hδ: 0.835
Gln_{12}	8.730	4.573	2.117, 1.977	Hγ: 2.345, HNt: 7.339, 6.869
Cys_C	8.748	4.877	3.189, 3.005	
NH_2	7.669, 7.252			

HPT-Ile $^{S}{}_{10}$ Test

Residue	NH	Hα	Hβ	Others
Ac		2.046		
Arg_1	8.310	4.384	1.772	Hγ: 1.655, Hδ: 3.219, HNt: 7.215
Thr_2	8.295	4.439	4.173	Hγ: 1.209
Val_3	8.156	4.251	2.124	Hγ: 0.935
Glu_4	8.386	4.721	1.905	Hγ: 2.327, 2.234
Val_5	8.536	4.549	2.030	Hγ: 0.951
pro_6		4.423	2.359	Hγ: 2.079, Hδ: 3.889, 3.818
Gly_7	8.437	3.985, 3.845		
Orn_8	7.993	4.517	1.890, 1.827	Hγ: 1.733, Hδ: 3.047, HNt: 7.619
Lys_9	8.44	4.439	1.733	Hγ: 1.373, Hδ: 1.420, Hε: 2.969, HNt: 7.554
Ile^{S}_{10}	8.497	4.486	1.983	Hγ: 1.514, 1.264, Hδ or Hγ (Me): 0.842
Leu_{11}	10.253	5.002	1.874	Hγ: 1.702, Hδ: 0.967, 0.888
Gln_{12}	8.514	4.345	2.124, 1.991	Hγ: 2.406, HNt: 7.406, 6.884
NH_2	7.605, 7.124			

HPT-Ile^S$_{10}$ Unfolded

Residue	NH	Hα	Hβ	Others
Ac		2.041		
Arg_1	8.313	4.348	1.830, 1.752	Hγ: 1.650, Hδ: 3.206, HNt: 7.207
Thr_2	8.282	4.380	4.176	Hγ: 1.189
Val_3	8.211	4.129	2.049	Hγ: 0.923
Glu_4	8.453	4.395	1.939	Hγ: 2.377
Val_5	8.327	4.426	2.080	Hγ: 0.954
Pro_6		4.414	2.324, 1.964	Hγ: 2.063, Hδ: 3.872, 3.708
Gly_7	8.474	3.957		
Orn_8	8.196	4.380	1.893	Hγ: 1.744, Hδ: 3.019, HNt: 7.618
Lys_9	8.356	4.301	1.783, 1.713	Hγ: 1.408, Hε: 2.972, HNt: 7.539
Ile^S_{10}	8.412	4.426	1.924	Hγ: 1.564, 1.220, Hδ or Hγ (Me): 0.845
Leu_{11}	10.19	4.958	1.861,	Hγ: 1.689, Hδ: 0.954, 0.876
Gln_{12}	8.484	4.333	2.112, 1.986	Hγ: 2.409, HNt: 7.434, 6.871
NH_2	7.597, 7.120			

HPT-Ile^S$_{10}$ Folded

Residue	NH	Hα	Hβ	Others
Ac		2.049		
Cys_N	8.429	4.880	3.113, 2.987	
Arg_1	8.672	4.583	1.830	Hγ: 1.642, Hδ: 3.206, HNt: 7.171
Thr_2	8.341	4.645	4.035	Hγ: 1.142
Val_3	7.851	4.426	2.143	Hγ: 0.892
Glu_4	8.355	4.927	1.877	Hγ: 2.237, 2.205
Val_5	8.852	4.583	2.002	Hγ: 0.938
pro_6		4.380	2.377, 1.997	Hγ: 2.143, 2.065, Hδ: 3.867
Gly_7	8.544	4.035, 3.785		
Orn_8	7.892	4.630	1.846	Hγ: 1.705, Hδ: 3.019, HNt: 7.619
Lys_9	8.529	4.645	1.720, 1.611	Hγ: 1.236, Hδ: 1.423, Hε: 2.941, HNt: 7.541
Ile^S_{10}	8.563	4.614	2.018	Hγ: 1.470, 1.236, Hδ or Hγ (Me): 0.845
Leu_{11}	10.393	4.974	1.830, 1.752	Hγ: 1.642, Hδ: 0.892
Gln_{12}	8.598	4.567	2.018	Hγ: 2.424, HNt: 7.334, 6.848
Cys_C	8.583	4.864	3.222, 3.019	
NH_2	7.649, 7.247			

HPT-Leu^S$_{11}$-OH Test

Residue	NH	Hα	Hβ	Others
Ac		2.036		
Arg_1	8.395	4.401	1.817, 1.738	Hγ: 1.629, Hδ: 3.195, HNt: 7.232
Thr_2	8.308	4.636	4.072	Hγ: 1.143
Val_3	8.516	4.260		Hγ: 0.877
Glu_4	8.421	4.746	1.832	Hγ: 2.130, 2.036
Val_5	8.636	4.558	1.973	Hγ: 0.916
pro_6		4.385	2.349, 2.005	Hγ: 2.083, Hδ: 3.879, 3.824
Gly_7	8.540	3.994, 3.790		
Orn_8	7.969	4.542	1.832	Hγ: 1.691, Hδ: 3.007
Lys_9	8.495	4.542	1.723	Hγ: 1.316, Hδ: 1.464, Hε: 2.96
Ile_{10}	8.620	4.338	1.926	Hγ: 1.363, 1.128, Hδ or Hγ (Me): 0.877
Leu^S_{11}	8.344	4.871	1.707	Hγ: 1.629, Hδ: 0.877
Gln_{12}	9.980	4.793	2.020	Hγ: 2.31, 2.208, HNt: 7.414, 6.824
OH				

HPT-Leu$^{S}_{11}$-OH Unfolded

Residue	NH	Hα	Hβ	Others
Ac		2.049		
Arg_1	8.312	4.364	1.767	Hγ: 1.658, Hδ: 3.222, HNt: 7.203
Thr_2	8.276	4.380		Hγ: 1.189
Val_3	8.203	4.129	2.065	Hγ: 0.923
Glu_4	8.447	4.411	2.033, 1.955	Hγ: 2.409
Val_5	8.318	4.442	2.080	Hγ: 0.954
Pro_6		4.42	2.331, 1.971	Hγ: 2.065, Hδ: 3.865, 3.706
Gly_7	8.469	3.957		
Orn_8	8.178	4.380	1.857	Hγ: 1.743, Hδ: 3.019, HNt: 7.606
Lys_9	8.400	4.333	1.814	Hγ: 1.423, Hδ: 1.720, Hε: 3.003, HNt: 7.544
Ile_{10}	8.270	4.176	1.893	Hγ: 1.470, 1.189, Hδ or Hγ (Me): 0.907
Leu^{S}_{11}	8.351	4.771	1.744	Hγ: 1.658, Hδ: 0.923
Gln_{12}	9.882	4.771	2.112	Hγ: 2.362, 2.276, HNt: 7.518, 6.827
OH				

HPT-Leu$^{S}_{11}$ Folded

Residue	NH	Hα	Hβ	Others
Ac		2.067		
Cys_N	8.440	5.184	3.164, 2.647	
Arg_1	8.828	4.683	1.848, 1.691	Hγ: 1.551, Hδ: 3.195, HNt: 7.136
Thr_2	8.638	4.980	3.947	Hγ: 1.096
Val_3	9.043	4.464	1.989	Hγ: 0.830
Glu_4	8.476	5.059	1.801	Hγ: 2.067, 1.942
Val_5	8.849	4.636	1.942	Hγ: 0.908
pro_6		4.338	2.396, 1.989	Hγ: 2.134, 2.067, Hδ: 3.864
Gly_7	8.721	4.072, 3.728		
Orn_8	7.882	4.667	1.817	Hγ: 1.691, Hδ: 3.007
Lys_9	8.529	4.793	1.738, 1.574	Hγ: 1.222, Hδ: 1.472, Hε: 2.944
Ile_{10}	9.082	4.542	1.942	Hγ: 1.284, 1.081, Hδ or Hγ (Me): 0.846
Leu^{S}_{11}	8.449	5.074	1.738, 1.613	Hγ: 1.457, Hδ: 0.799
Gln_{12}	10.595	5.482	2.005	Hγ: 2.263, HNt: 7.338, 6.842
Cys_C	8.993	5.106	3.132, 3.007	
NH_2	7.622, 7.253			

HPT-Leu$^{S}_{11}$-OH Test and Unfolded Epimerization. As noted in the main text, HPT-Leu$^{S}_{11}$-OH was synthesized as a C-terminal amide on Rink amide resin, but underwent thioamide catalyzed hydrolysis during TFA cleavage, accompanied by partial epimerization (see mechanism in **Fig. S4**). Two HPLC peaks were collected for each of the HPT-Leu$^{S}_{11}$-OH Test and HPT-Leu$^{S}_{11}$-OH Unfolded syntheses. While it could be assumed that epimerization was partial and that the larger peak corresponded to the desired epimer (with L-chirality at Gln$_{12}$), both peptides from each synthesis were characterized by NMR. CSD analysis of the HPT-Leu$^{S}_{11}$-OH epimers demonstrated that there is a significant difference between the β-hairpin character of the Test epimers, but not the Unfolded epimers (**Fig. S5**). The more abundant Test epimer (Test A) is as folded as HPT regardless of which Unfolded peptide is used as the reference, whereas the less abundant epimer (Test B) is the least folded of any of our HPT thioamide peptides. The results of the more abundant Test epimer are consistent with the results obtained for YKL-Leu$^{S}_{11}$-OH, wherein thioamide substitution was found to be non-perturbing. Based on this comparison, we assumed the most abundant Test peptide (Test A) was the L-Gln$_{12}$ epimer.

The more abundant Unfolded peptide (Unfolded A) was assumed to be the L-Gln$_{12}$ epimer and used as the reference since the ROESY data is similar for the two epimers, and the choice of Unfolded control has no effect on the CSD or fraction folded analysis. The fact that the stereochemistry of Gln$_{12}$ has a major effect on the spectra of the Test peptide, but a minor effect on the Unfolded control, further supports that the proGly β-turn is an effective Unfolded control. Fraction folded analysis for all combinations of Test and Unfolded peptides are shown in **Table S6**. For clarity, data reported elsewhere for HPT-Leu$^{S}_{11}$-OH Test correspond to Test A and data for HPT-Leu$^{S}_{11}$-OH Unfolded correspond to Unfolded A. Chemical shift assignments for the undesired epimers are given in **Table S7**.

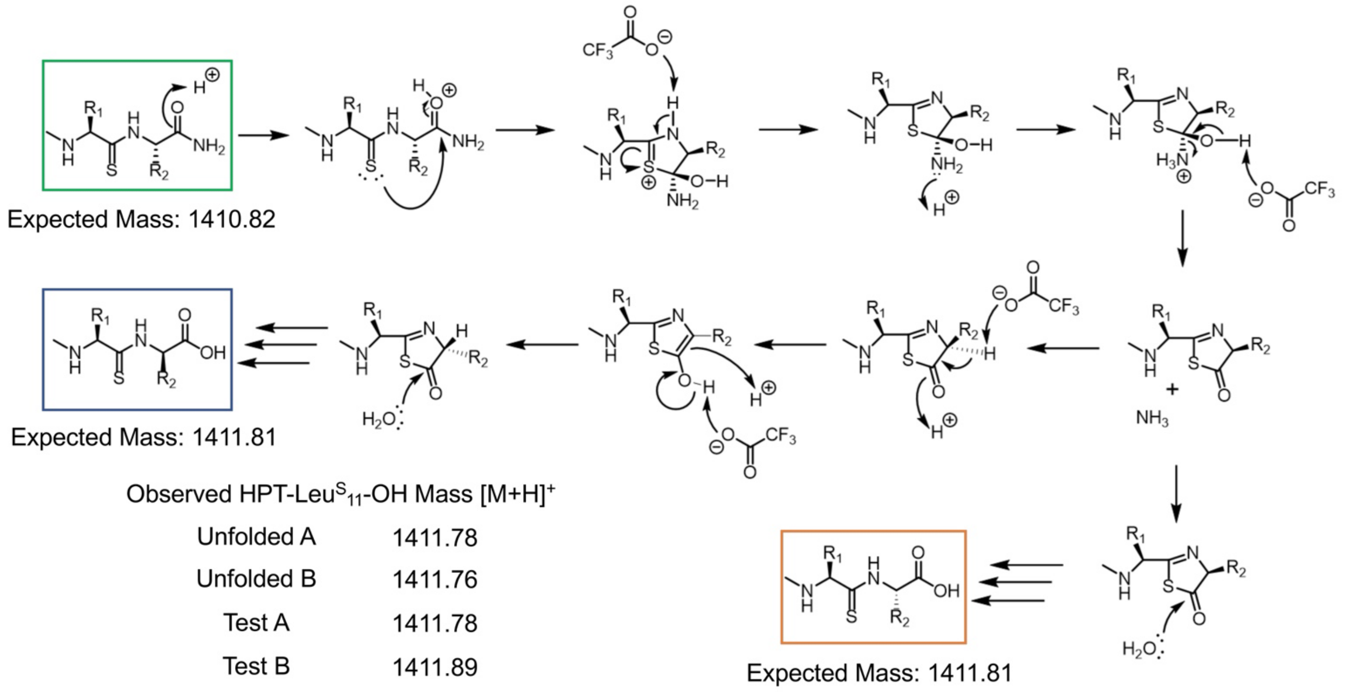

Figure S4. Proposed mechanism of Edman-type degradation observed for the HPT-Leu$^{S}_{11}$-OH Unfolded and Test β-hairpins. The observed masses correspond with epimerization of the C-terminal residue and a carboxylate C-terminus. As described above, the L-epimers are Unfolded A and Test A.

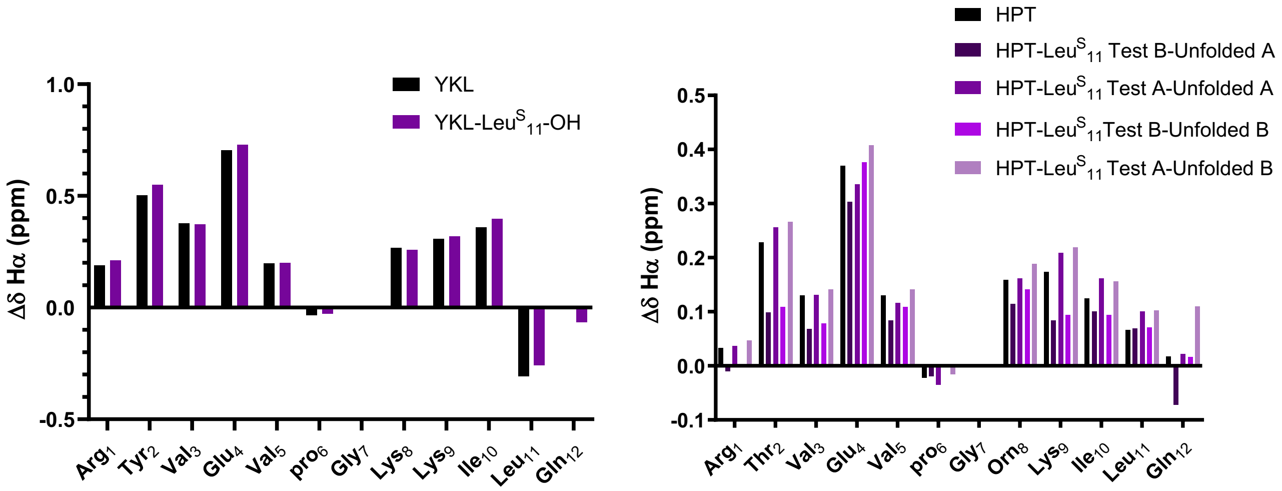

Figure S5. CSD analysis ($\delta_{H\alpha}$) of the HPT-Leu$^S_{11}$-OH epimers in comparison to YKL-Leu$^S_{11}$-OH demonstrates that HPT-Leu$^S_{11}$-OH Test epimer A is folded similarly to YKL-Leu$^S_{11}$-OH Test and that HPT-Leu$^S_{11}$-OH Test epimer B is poorly folded.

Table S6. Comparison of fraction folded and $\Delta G_{Folding}$ analysis for HPT-Leu$^S_{11}$-OH Test and Unfolded epimers

	**Fraction Folded (%)**			**$\Delta G_{Folding}$ (kcal/mol)**		
**HPT-Leu$^S_{11}$-OH**	**Val$_3$**	**Ile$_{10}$**	**Average**	**Val$_3$**	**Ile$_{10}$**	**Average**
Oxo	38.6%	37.7%	38.1 ± 1%	0.28	0.30	0.29 ± 0.0
Test B - Unfolded A	20.3%	27.3%	23.8 ± 4%	0.81	0.58	0.69 ± 0.1
Test B - Unfolded B	22.6%	26.1%	24.4 ± 2%	0.73	0.62	0.67 ± 0.1
Test A - Unfolded A	39.1%	44.3%	41.7 ± 3%	0.26	0.14	0.20 ± 0.1
Test A - Unfolded B	40.9%	43.3%	42.1 ± 1%	0.22	0.16	0.19 ± 0.0

Table S7. ^{1}H Chemical shift assignments for HPT-Leu$^S_{11}$-OH Test and Unfolded epimers

HPT-Leu$^S_{11}$-OH Test D-Gln$_{12}$ Epimer (Test B)

Residue	NH	Hα	Hβ	Others
Ac		2.036		
Arg$_1$	8.293	4.354	1.832, 1.754	Hγ: 1.644, Hδ: 3.195, HNt: 7.232
Thr$_2$	8.235	4.479	4.135	Hγ: 1.190
Val$_3$	8.259	4.197	2.052	Hγ: 0.908
Glu$_4$	8.407	4.714	1.879	Hγ: 2.271, 2.177
Val$_5$	8.527	4.526	2.005	Hγ: 0.940
pro$_6$		4.401	2.349, 2.005	Hγ: 2.083, Hδ: 3.884, 3.808
Gly$_7$	8.441	3.978, 3.821		
Orn$_8$	7.976	4.495	1.801	Hγ: 1.707, Hδ: 3.023
Lys$_9$	8.502	4.417	1.754	Hγ: 1.347, Hδ: 1.472, Hε: 2.976
Ile$_{10}$	8.447	4.276	1.926	Hγ: 1.41, 1.175, Hδ or Hγ (Me): 0.908, 0.814
Leu$^S_{11}$	8.286	4.840	1.738	Hγ: 1.644, Hδ : 0.908
Gln$_{12}$	9.781	4.699	2.099	Hγ: 2.349, 2.255, HNt: 7.530, 6.817
OH				

HPT-Leu$^S_{11}$-OH Unfolded D-Gln$_{12}$ Epimer (Unfolded B)

Residue	NH	Hα	Hβ	Others
Ac		2.036		
Arg$_1$	8.308	4.354	1.825, 1.738	Hγ: 1.644, Hδ: 3.208, HNt: 7.219
Thr$_2$	8.291	4.370	4.182	Hγ: 1.19
Val$_3$	8.204	4.119	2.052	Hγ: 0.908
Glu$_4$	8.469	4.338	2.255	Hγ: 1.911
Val$_5$	8.357	4.417	2.067	Hγ: 0.955
Pro$_6$		4.401	2.318, 1.95	Hγ: 2.052, Hδ: 3.874, 3.704
Gly$_7$	8.490	3.947		
Orn$_8$	8.181	4.354	1.864	Hγ: 1.707, Hδ: 3.007, HNt:
Lys$_9$	8.418	4.323		Hγ: 1.410, Hδ: 1.731, Hε: 2.991, HNt:
Ile$_{10}$	8.297	4.182	1.895	Hγ: 1.457, 1.190, Hδ or Hγ (Me): 0.908
Leu$^S_{11}$	8.311	4.769	1.754	Hγ: 1.629, Hδ: 0.908
Gln$_{12}$	9.689	4.683	2.083	Hγ: 2.334, 2.240, HNt: 7.523, 6.820
OH				

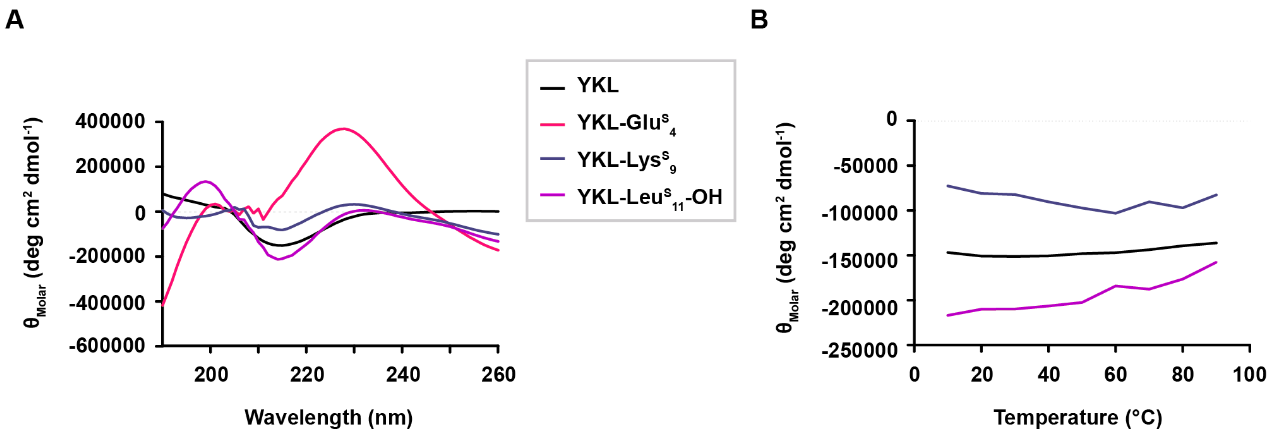

Figure S6. (**A**) Far-UV circular dichroism (CD) of thioamide-containing Test YKL β-hairpins. All data were collected at ~100 μM concentration in 100 mM sodium acetate buffer (pH = 3.8). (**B**) Temperature-dependent denaturation of thioamide-containing Test YKL β-hairpins. The thermal melts measured at this signature were linear and therefore T_m could not be calculated. The YKL-Glu$^S{}_4$ has not been considered for the analysis due to lack of β-hairpin signature.

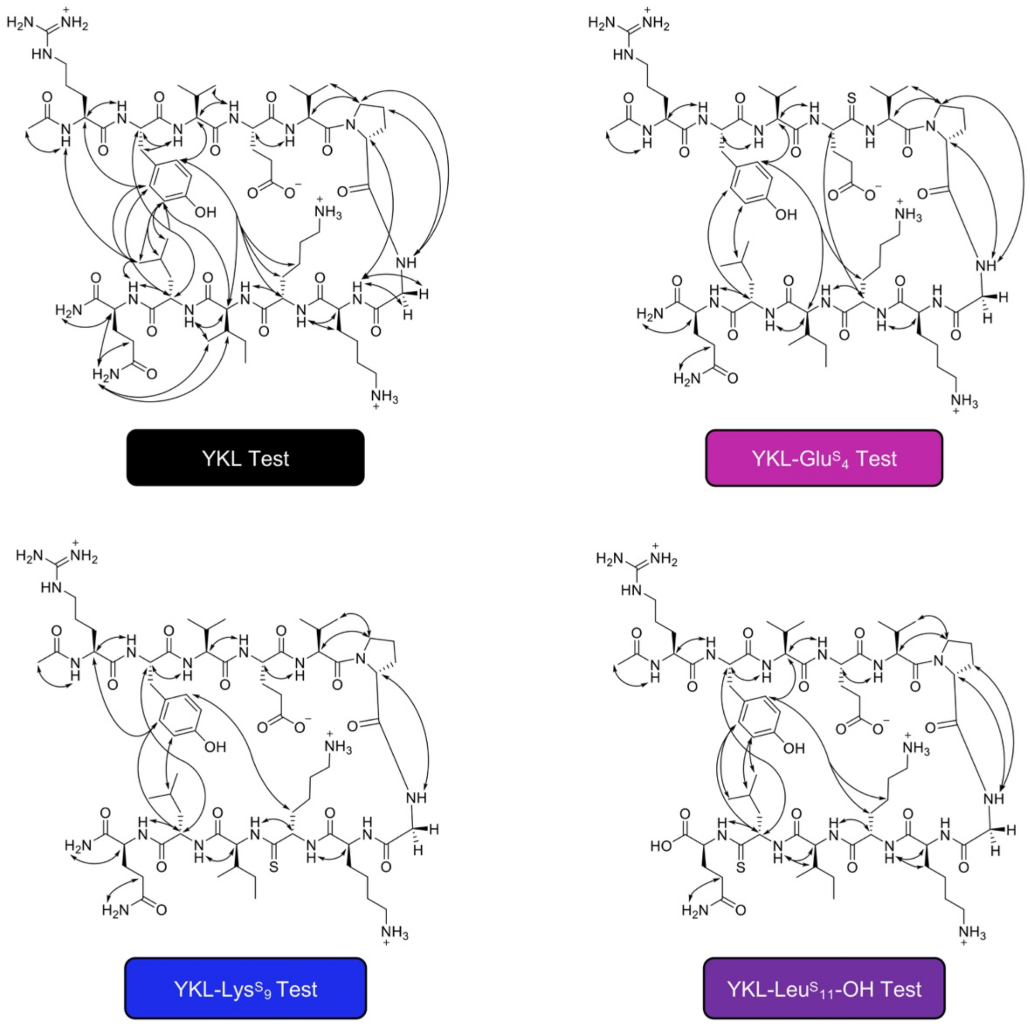

Figure S7. NOEs for Test YKL β-hairpin variants.

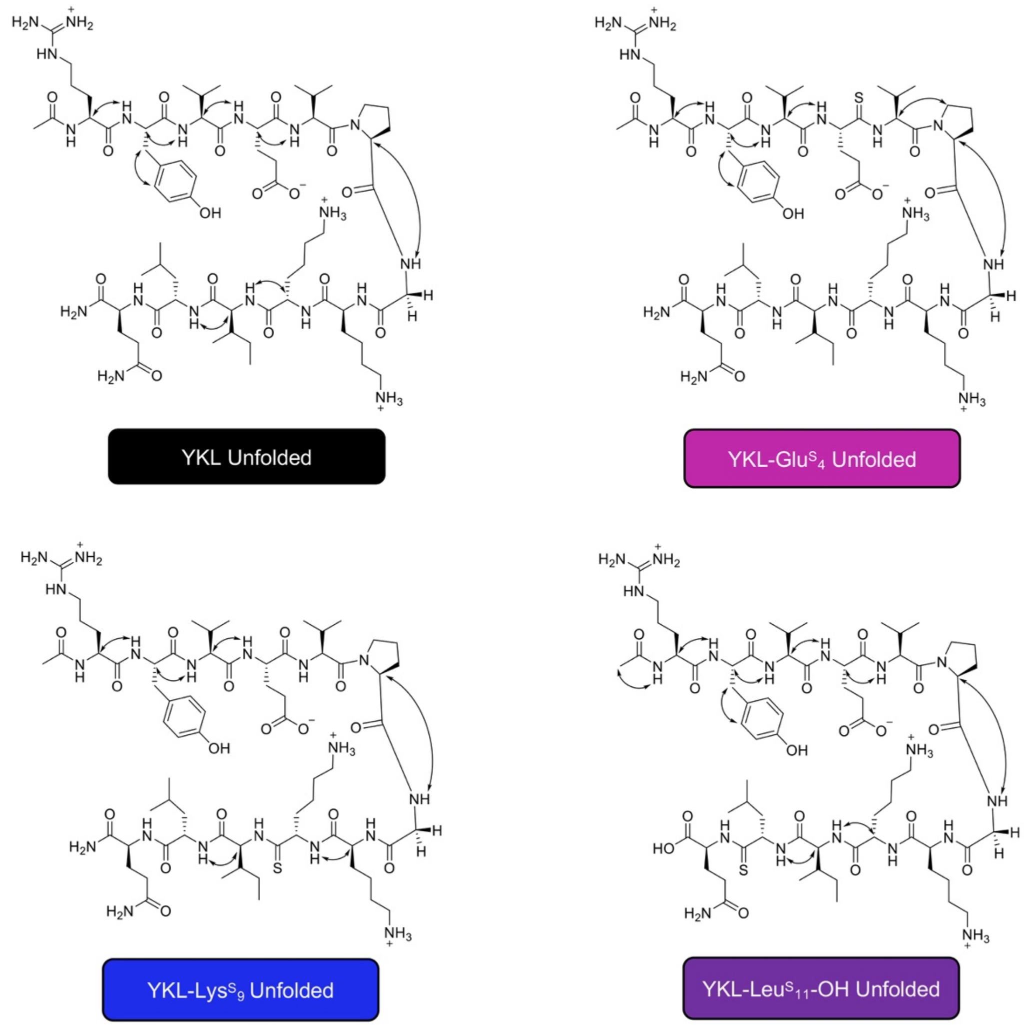

Figure S8. NOEs for Unfolded YKL β-hairpin variants.

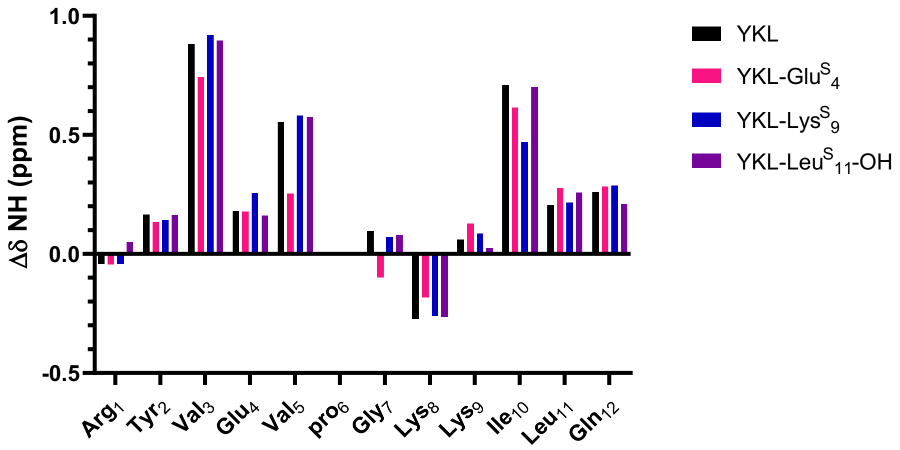

Figure S9. $\Delta\delta_{NH}$ (Test δ_{NH} – Unfolded control δ_{NH}) for YKL-Glu$^{S}{}_{4}$, YKL-Lys$^{S}{}_{9}$, and YKL-Leu$^{S}{}_{11}$-OH in comparison to YKL. The thioamide variants have similar values to YKL suggestive of a similar structure.

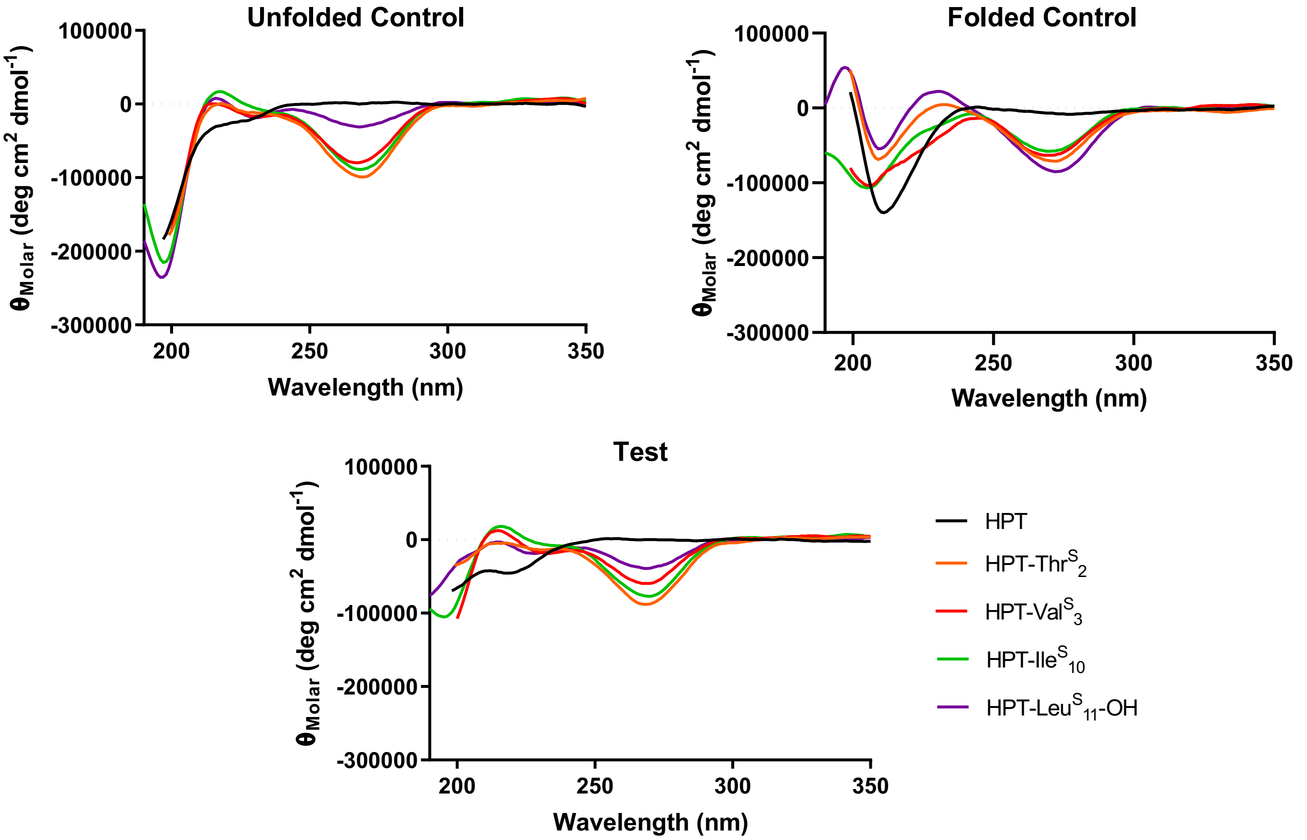

Figure S10. CD scans of thioamide variants of the HPT β-hairpin scaffold. All data were collected at ~130-150 μM in the buffer corresponding to the NMR buffer (HPT, HPT-Thr$^{S}{}_{2}$, and HPT-Val$^{S}{}_{3}$ in 50 mM sodium deuterioacetate pH 5.5 and HPT-Ile$^{S}{}_{10}$ and HPT-Leu$^{S}{}_{11}$-OH in 50 mM NaH_2PO_4 pH 5.5). The Folded controls exhibit the most significant anti-parallel β-sheet character (slight differences can be observed for the internal thioamides: HPT-Val$^{S}{}_{3}$ and HPT-Ile$^{S}{}_{10}$). The Test β-hairpins have a stronger thioamide π-to-π* absorbance than any secondary structure characteristic.

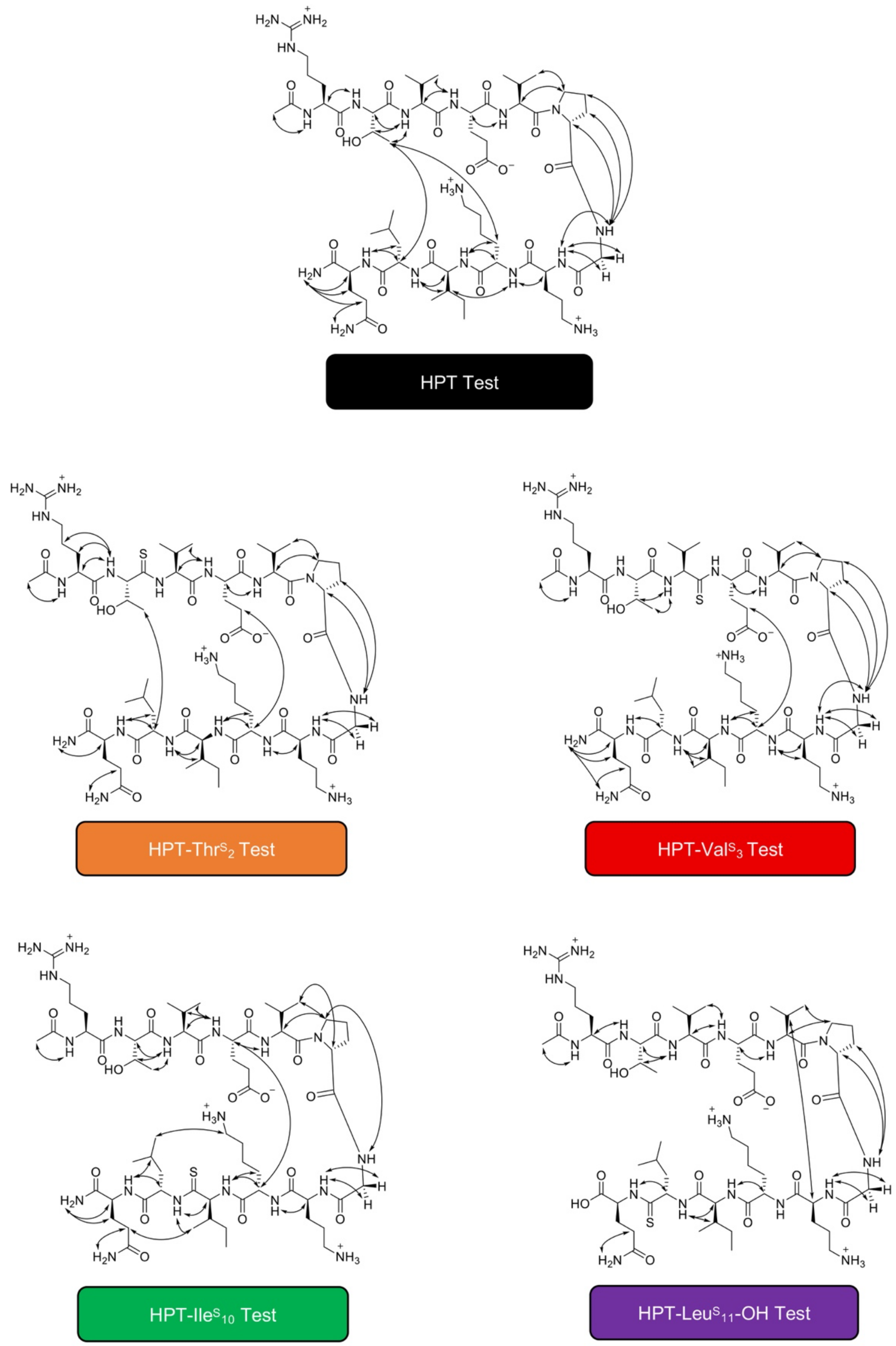

Figure S11. NOEs for Test HPT β-hairpin variants.

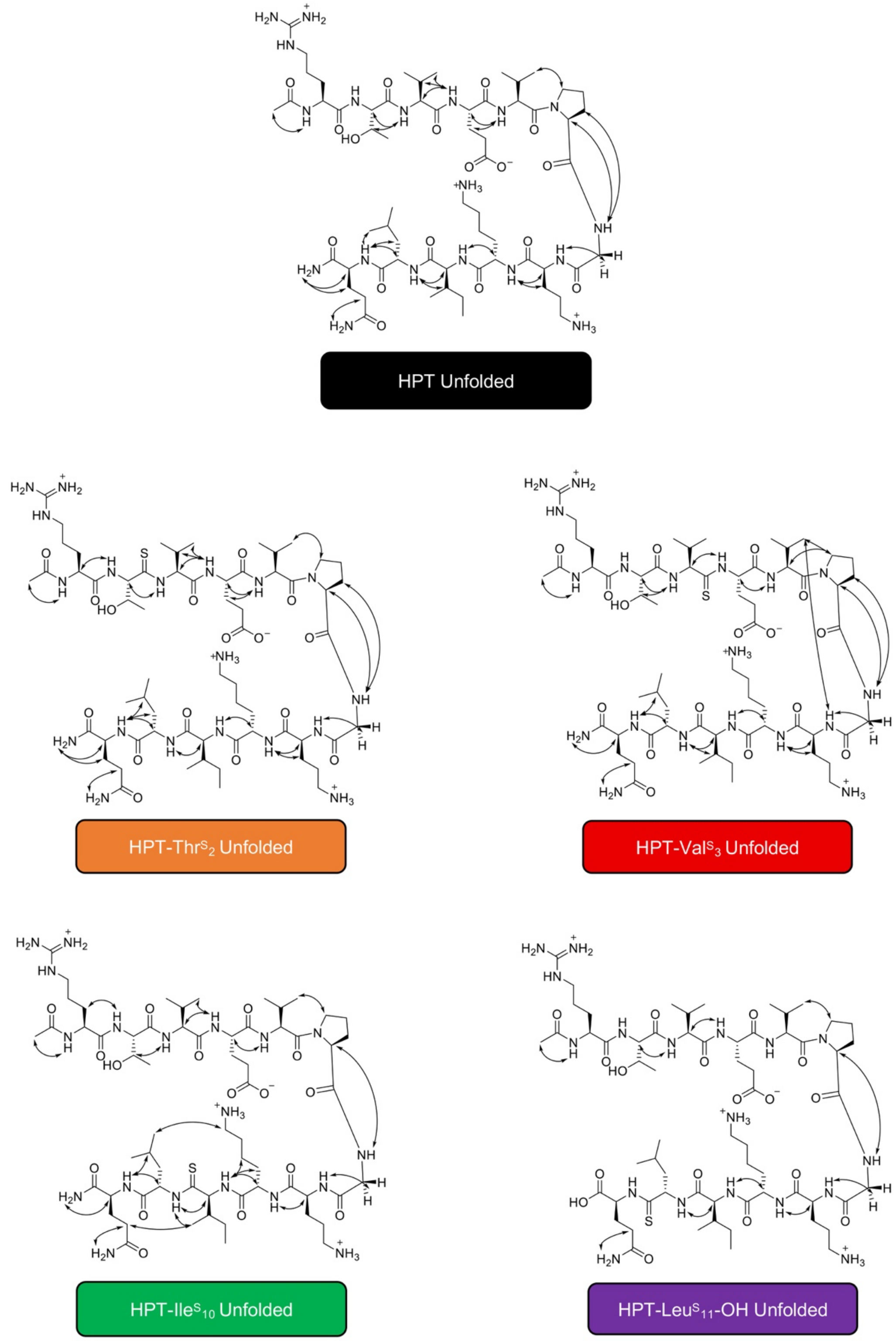

Figure S12. NOEs for Unfolded HPT β-hairpin variants.

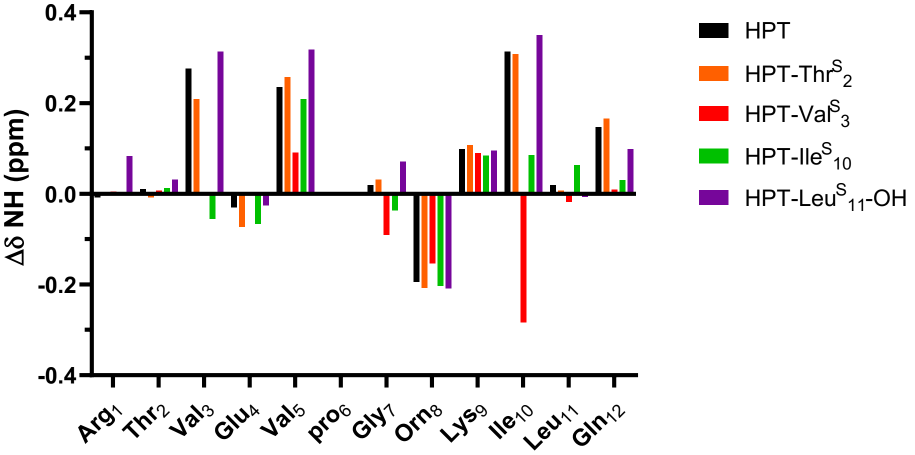

Figure S13. $\Delta\delta_{NH}$ (Test δ_{NH} – Unfolded control δ_{NH}) for HPT-Thr$^S{}_2$, HPT-Val$^S{}_3$, HPT-Ile$^S{}_{10}$ and HPT-Leu$^S{}_{11}$-OH in comparison to HPT. Overall, no major trends are observed. Of interest is the negative $\Delta\delta_{NH}$ for the amide across from the HPT-Val$^S{}_3$ thioamide. This is probably due to the increased electron density of the thioamide. This negative $\Delta\delta_{NH}$ is weakly observed for the other hydrogen bond acceptor position (HPT-Ile$^S{}_{10}$).

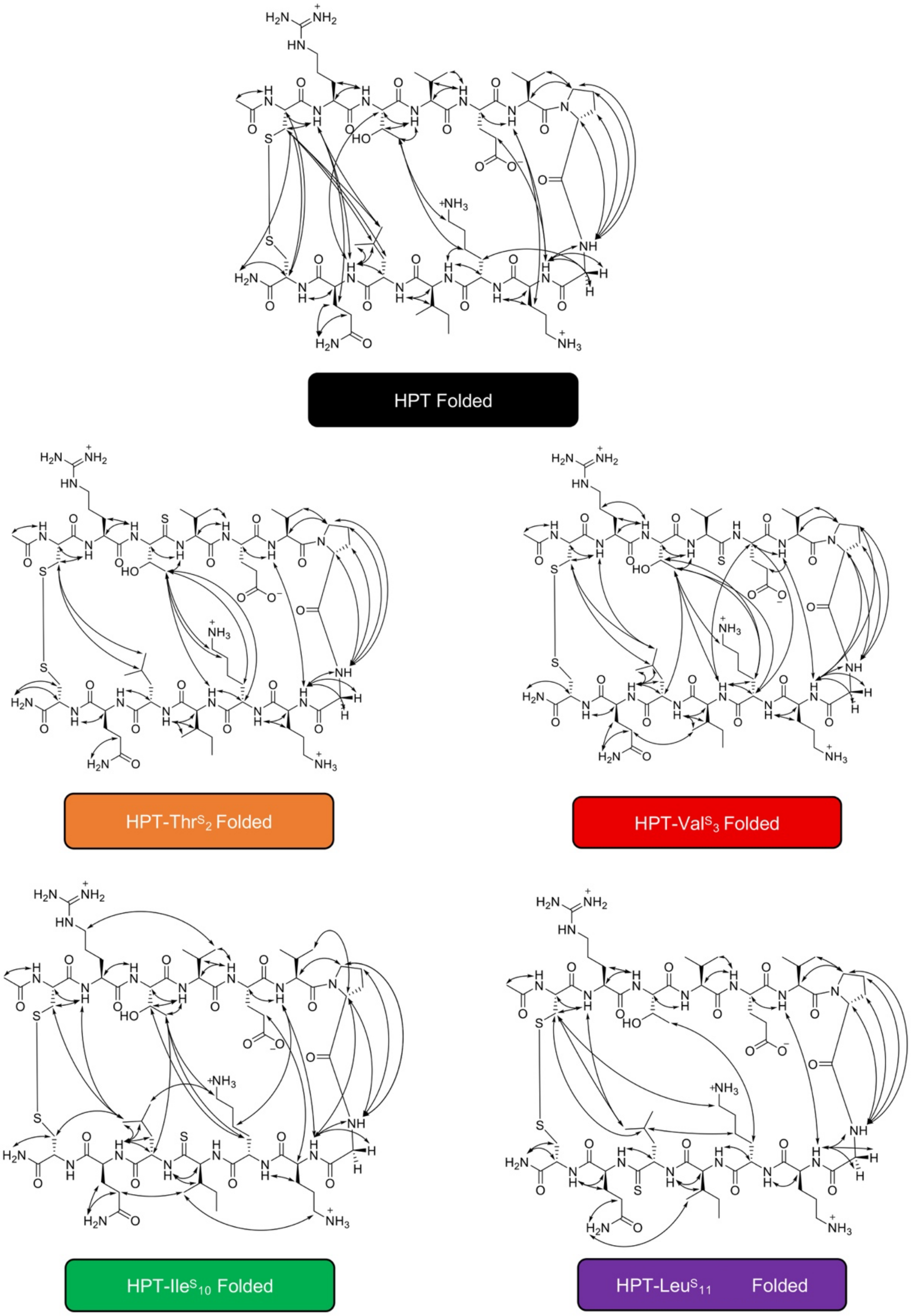

Figure S14. NOEs for Folded HPT β-hairpin variants.

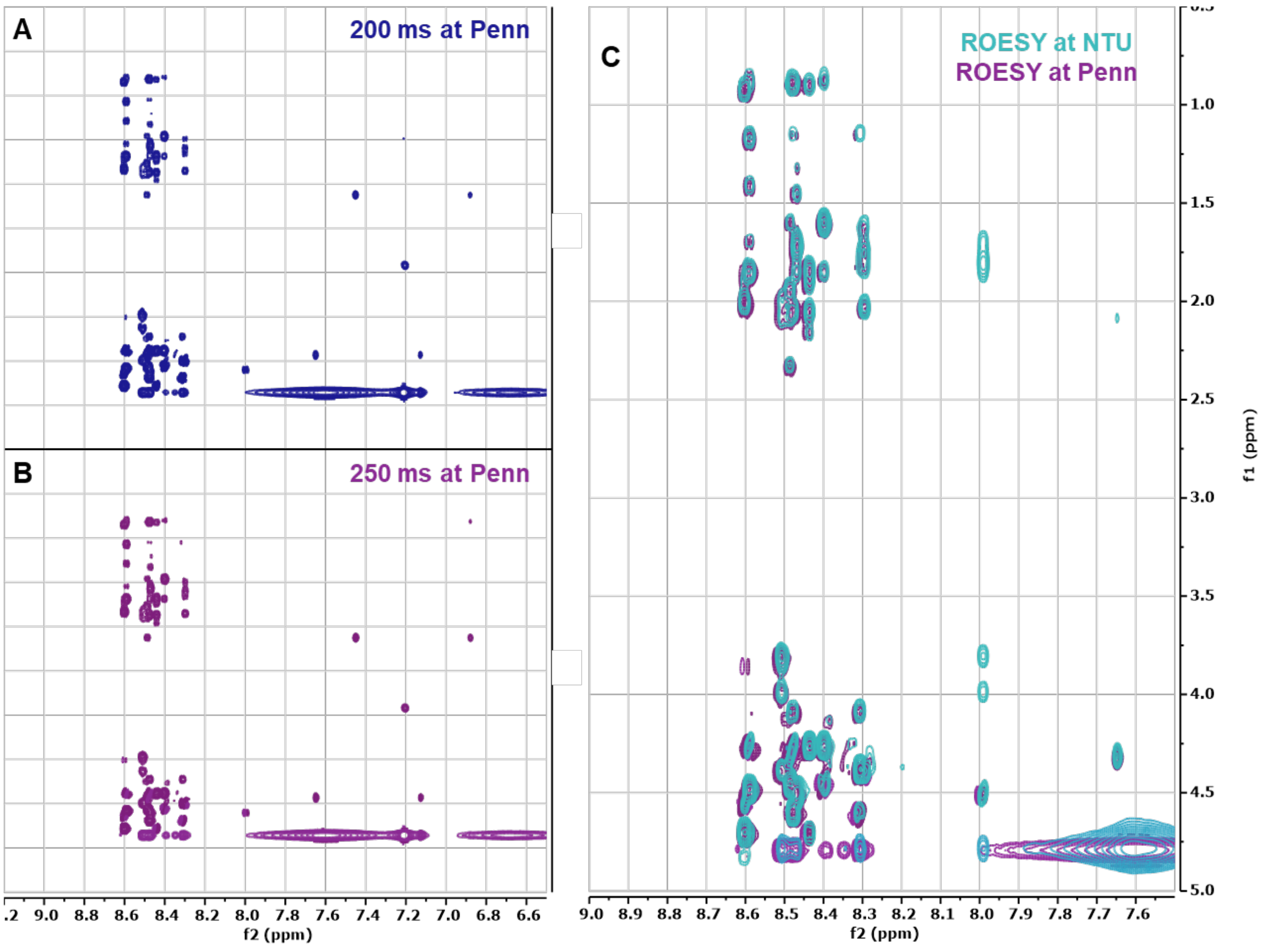

Figure S15. ROESY mixing time comparison for the HPT scaffold. HPT HPT-Thr$^S{}_2$, and HPT-Val$^S{}_3$ were collected at NTU (200 ms mixing time). Whereas HPT-Ile$^S{}_{10}$ and HPT-Leu$^S{}_{11}$-OH were collected at Penn (250 ms mixing time). Since the NOE connectivities are directly observed from the spectra, a comparison was needed between the two collection methods. This was done by collecting ROESY data of the HPT Test peptide with the two mixing times (**A** and **B**). No additional cross-peaks are observed with the longer mixing time (250 ms). (**C**) is the overlay of a ROESY collected at NTU versus collected at Penn. Besides a lack of Orn$_8$ signal in the Penn spectra, the cross-peaks are similar between the two collection methods. One difference between the spectra is that the intensities are on different scales. For example, the NOE between Val$_5$ Hα and pro$_6$ Hδ is ~4.5 for the NTU collection and ~5400 for the Penn collection. This 1000x fold difference is reflected in the internal intensity cut-off for the NOEs displayed in **Fig. S11-12**, **S14** (0.3 for NTU and 300 for Penn).

Table S8. Fraction folded and $\Delta\Delta G_{Folding}$ analysis of HPT peptides

	Experimental					
	**Fraction Folded (%)**			**$\Delta\Delta G_{Folding}$ (kcal/mol)**		
	**Val_3**	**Ile_{10}**	**Average**	**Val_3**	**Ile_{10}**	**Average**
HPT	38.6%	37.7%	38.1 ± 0.5%	---	----	----
HPT-Thr^{S}_{2}	34.4%	41.1%	37.8 ± 3%	0.11	-0.09	0.01 ± 0.1
HPT-Val^{S}_{3}	24.5%	24.5%	24.5 ± 0%	0.39	0.37	0.38 ± 0.01
HPT-Ile^{S}_{10}	41.1%	31.9%	36.5 ± 5%	-0.06	0.15	0.04 ± 0.1
HPT-Leu^{S}_{11}-OH	39.1%	44.3%	41.7 ± 3%	-0.01	-0.16	-0.09 ± 0.1

Structural Models of the Thioamide-Containing Folded Control β-Hairpins

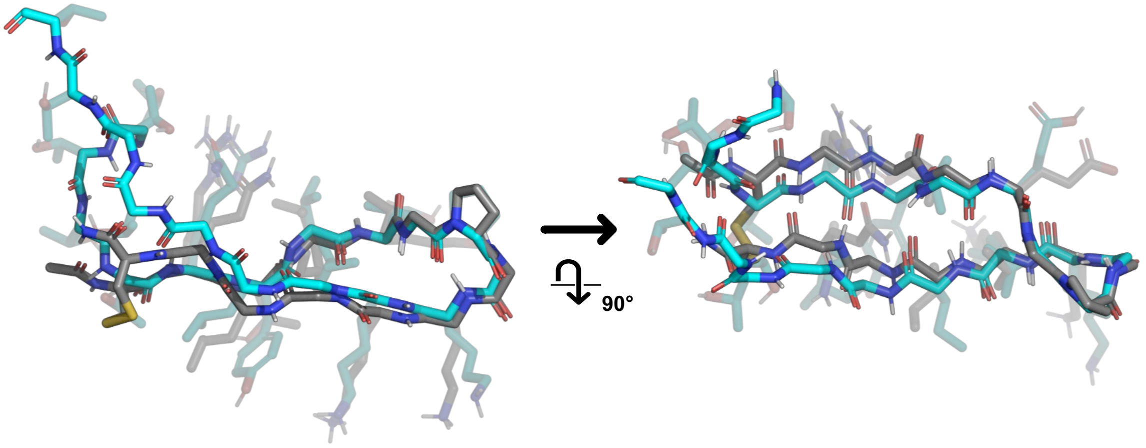

Figure S16. The structure of YKL with a Thr extension (PDB 1jy9) was used as a starting point to generate a structure for HPT. The following modifications were made using PyRosetta[11]: deletion of the terminal Thr residues, conversion of the penultimate Thr residues to Cys, formation of the disulfide bond, acetylation of the N-terminus, conversion of the C-terminus to a carboxamide, conversion of Tyr_2 to Thr, and conversion of Lys_8 to Orn. Upon completion of these modifications, it can be seen that the starting model of HPT (gray) exhibits similar backbone and side-chain orientations to the starting 1jy9 structure (cyan).

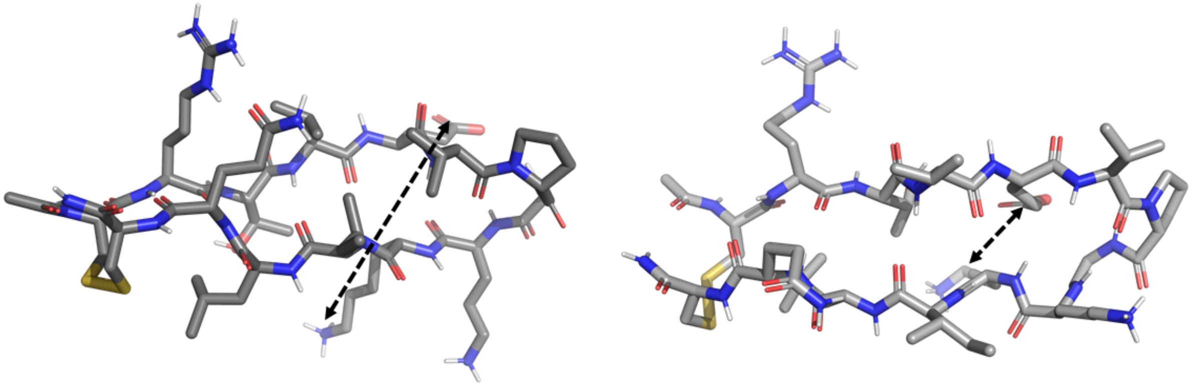

Before Distance Constraints **After Distance Constraints**

Figure S17. The HPT starting structure was subjected to ten Cartesian FastRelax cycles employing the beta_nov16Cart score function in PyRosetta. Additionally, constrained relaxes were performed using the same conditions and the distances derived from the HPT Folded control NOEs (**Table S9**). The resulting lowest energy structure from the constrained relax differed significantly from the initial model (backbone RMSD = 2.231 Å) but was consistent with the NOEs displaying only three instances of deviation greater than 0.2 Å between the experimental and computationally derived distances (one in the β-turn and two between Cys_N and Leu_{12}). Of particular interest is that removal of the cross-strand cation-π interaction of the YKL scaffold resulted in Glu_4 and Lys_9 forming a salt bridge (indicated by arrows) which was previously not observed in the HPT structure prior to the constrained relax.

Generation of Distance Constraints from NOEs. The *trans*-Val-pro bond has a fixed interproton distance of ~2.1 Å, which was previously used as an internal standard for converting NOE intensities to distances in a pro-Gly β-hairpin.[12] Therefore, **Eq. S2** was used to convert NOE intensities to experimental distances, where r_{ref} is 2.1 Å, a_{ref} is the NOE intensity corresponding to pro_7 Hδ- Val_6 Hα cross-peak, a_{ij} and r_{ij} correspond respectively to the NOE intensity and distance of the atom pair.[13]

$$r_{ij} = r_{ref}\left(\frac{a_{ref}}{a_{ij}}\right)^{1/6} \quad \textbf{(Eq. S2)}$$

PyRosetta Modelling of Folded Controls. The experimental distances listed in **Table S9** were entered into PyRosetta and used for constrained relax in the following way. A flat harmonic potential was used with a standard deviation and tolerance of 0.1. The weight of the constraint term passed to the score function was 1.0. The thioamide modification was incorporated through our previously generated patches[14] during these simulations, which produced models of the HPT, HPT-Thr^S_2, HPT-Val^S_3, HPT-Ile^S_{10} and HPT-Leu^S_{11}-OH Folded controls. The computed interatom distances are given in **Table S9** for comparison to the NOE values. The backbone RMSD of the thioamide-containing Folded controls in comparison to the HPT Folded control are displayed in **Table S10**.

Table S9. NOE derived distance constraints for PyRosetta simulation of Folded control β-hairpins

The Folded control peptide numbering starts with Cys_1 (Cys_N), Arg_2, Thr_3, etc. The atom names are represented in the PDB format. Atom names in red correspond to instances where the specific atom number could not be determined based on the chemical shifts observed, and therefore an arbitrary number was chosen. Any deviation of greater than 0.15 Å between the experimental and computational distances is highlighted in red. Note: Experimental distances were only determined to 0.1 Å precision.

HPT Folded Control

Constraint number	First Res Atom Name	First Res number	Second Res Atom Name	Second Res number	Intensity	Experimental Distance	Computational Distance	Deviation
**0**	H	8	1HD	7	1	3.1	3.24	0.14
**1**	H	9	2HA	8	0.4	3.6	3.72	0.12
**2**	H	9	1HA	8	0.5	3.5	3.35	0.15
**3**	H	6	H	9	0.4	3.6	3.48	0.12
**4**	H	8	H	9	0.8	3.2	3.25	0.05
**5**	H	13	H	2	0.3	3.8	3.70	0.10
**6**	H	6	1HB	9	0.4	3.6	3.59	0.01
**7**	H	2	1HD1	12	0.3	3.8	3.69	0.11
**8**	H	2	1HB	13	0.3	3.8	3.91	0.11
**9**	H	8	2HB	7	1.6	2.9	3.03	0.13
**10**	H	8	1HG	7	0.6	3.4	3.51	0.11
**11**	H	9	1HG	5	0.4	3.6	3.71	0.11
**12**	HA	1	HA	14	2.6	2.6	2.47	0.13
**13**	1HD	7	HA	6	10	2.1	2.00	0.10
**14**	1HD	7	1HG1	6	1.4	2.9	3.07	0.17
**15**	1HE	10	1HG2	3	0.4	3.6	3.70	0.10
**16**	2HB	1	1HD1	12	1	3.1	2.97	0.13
**17**	2HB	1	HG	12	1	3.1	2.94	0.16
**18**	2HB	1	1HB	12	0.5	3.5	3.72	0.22
**19**	2HB	1	HA	14	0.6	3.4	3.52	0.12
**20**	1HB	10	2HA	8	0.8	3.2	3.33	0.13
**21**	1HG2	3	1HG	10	1.8	2.8	2.90	0.10
**22**	H	13	HA	3	0.3	3.8	3.68	0.12
**23**	H	8	HA	7	1.4	2.9	3.02	0.12

HPT-Thr^{S}$_2$ Folded Control

Constraint number	First Res Atom Name	First Res number	Second Res Atom Name	Second Res number	Intensity	Experimental Distance	Computational Distance	Deviation
**0**	H	9	1HA	8	0.3	3.2	3.09	0.11
**1**	H	9	2HB	8	0.3	3.2	3.33	0.13
**2**	H	8	2HB	7	0.5	3	3.11	0.11
**3**	H	8	1HG	7	0.3	3.2	3.33	0.13
**4**	H	6	H	9	0.3	3.2	3.09	0.11
**5**	H	8	H	9	0.4	3.1	2.99	0.11
**6**	1HD	7	1HG1	6	0.6	2.9	3.07	0.17
**7**	1HD	7	HA	6	4	2.1	2.01	0.09
**8**	1HG2	3	HA	10	1	2.6	2.50	0.10
**9**	1HG2	3	1HD	10	1.1	2.6	2.70	0.10
**10**	1HG2	3	2HB	10	0.5	3	2.92	0.08
**11**	1HG2	3	H	11	0.3	3.2	3.32	0.12
**12**	1HG2	3	1HE	10	0.3	3.2	3.10	0.10
**13**	H	8	1HD	7	0.4	3.1	3.22	0.12
**14**	1HB	1	1HD1	12	0.45	3	2.90	0.10
**15**	1HB	1	HG	12	0.4	3.1	3.21	0.11
**16**	H	8	HA	7	0.6	2.9	3.03	0.13

HPT-Val $^{S}{}_{3}$ Folded Control

Constraint number	First Res Atom Name	First Res number	Second Res Atom Name	Second Res number	Intensity	Experimental Distance	Computational Distance	Deviation
**0**	H	9	HA	7	0.6	3.8	3.61	0.19
**1**	H	11	HA	5	0.4	4	4.1	0.10
**2**	H	2	1HD1	12	0.4	4	4.1	0.10
**3**	H	8	1HG	7	1	3.5	3.66	0.16
**4**	H	8	2HB	7	1.3	3.3	3.16	0.14
**5**	H	9	2HG	7	0.6	3.8	4.04	0.24
**6**	H	6	H	9	0.6	3.8	3.71	0.09
**7**	HA	10	HA	5	1.2	3.3	3.4	0.10
**8**	HB	3	2HB	10	0.4	4	3.9	0.10
**9**	1HD	7	HA	6	19.7	2.1	2.04	0.06
**10**	1HD	7	1HG1	6	2.4	3	3.17	0.17
**11**	2HB	1	1HD1	12	0.7	3.7	3.7	0.00
**12**	1HE	10	1HG2	3	0.5	3.9	4	0.10
**13**	2HB	1	HG	12	0.4	4	4.11	0.11
**14**	2HB	1	1HD1	12	0.6	3.8	3.7	0.10
**15**	1HG	13	1HG2	11	0.8	3.6	3.7	0.10
**16**	1HG2	3	HA	12	0.8	3.6	3.72	0.12
**17**	1HG2	3	HA	10	0.9	3.5	3.61	0.11
**18**	2HB	10	1HG2	3	1.3	3.3	3.19	0.11
**19**	H	9	2HA	8	1.2	3.3	3.44	0.14
**20**	H	9	1HA	8	1.3	3.3	3.4	0.10
**21**	H	8	H	9	0.9	3.5	3.33	0.17
**22**	H	8	HA	7	2.3	3	3.15	0.15

HPT-Ile^{S}$_{10}$ Folded Control

Constraint number	First Res Atom Name	First Res number	Second Res Atom Name	Second Res number	Intensity	Experimental Distance	Computational Distance	Deviation
**0**	H	6	H	9	506.4	4.2	4.07	0.13
**1**	H	6	2HG	10	126.3	5.3	5.42	0.12
**2**	H	2	1HD1	12	138	5.2	5.31	0.11
**3**	H	8	1HG	7	975.2	3.8	3.92	0.12
**4**	H	8	H	9	1451.6	3.5	3.37	0.13
**5**	H	8	HA	7	3459.4	3.1	3.24	0.14
**6**	H	8	1HD	7	1180.6	3.7	3.56	0.14
**7**	1HE2	13	1HG2	11	597.2	4.1	4.2	0.10
**8**	HA	7	H	9	565.6	4.1	4.21	0.11
**9**	HA	7	1HG1	6	1114.7	3.7	3.83	0.13
**10**	HB	3	2HB	10	772.7	3.9	4	0.10
**11**	2HA	8	H	9	669	4	3.84	0.16
**12**	1HD	2	1HG1	4	478.5	4.3	4.32	0.02
**13**	1HB	1	1HD1	12	517.9	4.2	4.1	0.10
**14**	1HD	9	1HG2	11	502.3	4.2	4.32	0.12
**15**	1HE	10	1HD1	12	400.3	4.4	4.51	0.11
**16**	1HE	10	1HG2	3	702.5	4	3.89	0.11
**17**	1HG	5	HA	9	469.8	4.3	4.43	0.13
**18**	2HG	5	HA	9	414.1	4.4	4.51	0.11
**19**	1HG2	3	2HB	10	1443.7	3.5	3.6	0.10
**20**	1HG2	3	HA	12	518.8	4.2	4.22	0.02
**21**	1HD1	12	2HB	1	923.6	3.8	3.7	0.10
**22**	1HD	7	HA	6	33171.2	2.1	2.2	0.10

HPT-Leu$^{S}_{11}$ Folded Control

Constraint number	First Res Atom Name	First Res number	Second Res Atom Name	Second Res number	Intensity	Experimental Distance	Computational Distance	Deviation
**0**	H	6	H	9	649	3.8	3.69	0.11
**1**	H	2	1HD1	12	428	4	4.13	0.13
**2**	H	8	H	9	930.4	3.5	3.61	0.11
**3**	H	8	1HG	7	549.5	3.9	4.02	0.12
**4**	H	8	2HB	7	1198.5	3.4	3.52	0.12
**5**	H	8	HA	7	1533.3	3.3	3.28	0.02
**6**	H	8	1HD	7	794.5	3.6	3.71	0.11
**7**	2HE2	13	1HG2	11	306.9	4.3	4.40	0.10
**8**	1HD	7	HA	6	21267.6	2.1	2.00	0.10
**9**	1HA	8	H	9	297	4.3	4.08	0.22
**10**	1HD	7	1HG1	6	2402.9	3	3.17	0.17
**11**	2HA	8	H	9	282	4.3	4.14	0.16
**12**	1HB	1	1HE	10	2297.6	3	3.12	0.12
**13**	1HB	1	1HD1	12	830.3	3.6	3.49	0.11
**14**	HG	12	1HG	10	158.1	4.8	4.69	0.11
**15**	1HG2	3	2HB	10	809.6	3.6	3.74	0.14
**16**	2HB	1	HG	12	850	3.6	3.48	0.12
**17**	2HB	1	1HB	12	509	3.9	4.02	0.12

Table S10. Backbone RMSD of the thioamide-containing Folded control structures in comparison to the HPT Folded control

	**Backbone RMSD (Å)**
HPT-Thr$^{S}_{2}$	1.14
HPT-Val$^{S}_{3}$	2.15
HPT-Ile$^{S}_{10}$	0.63
HPT-Leu$^{S}_{11}$	1.23

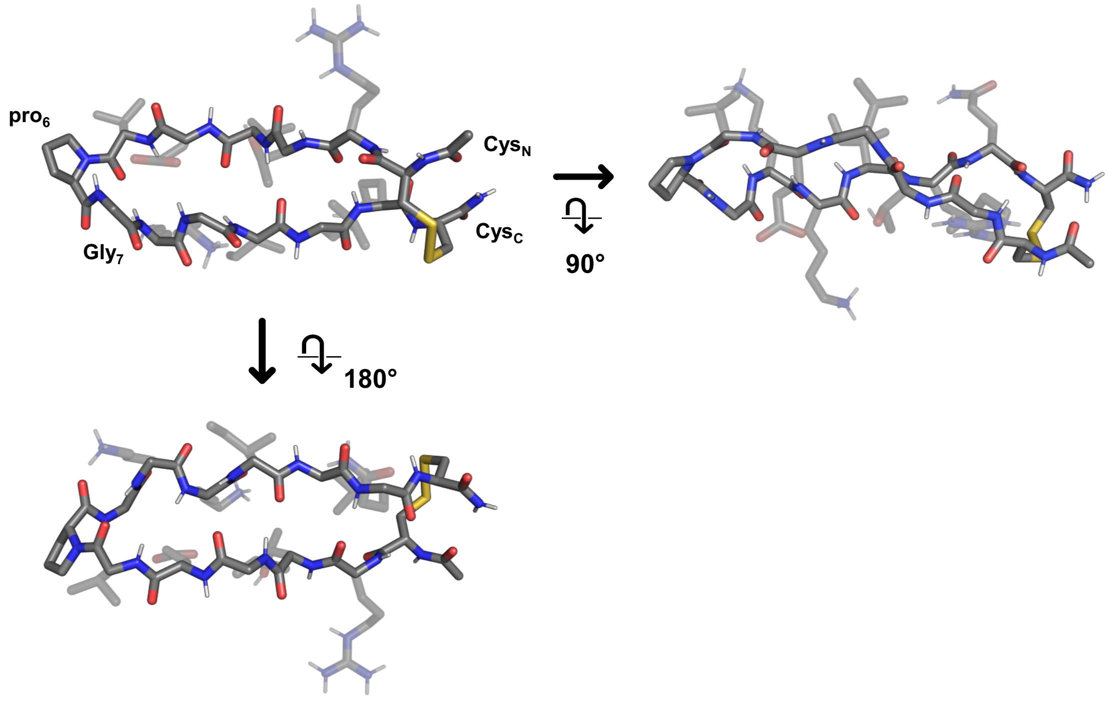

Figure S18. Modelled structure of HPT Folded control peptide based on NOE derived distance constraints

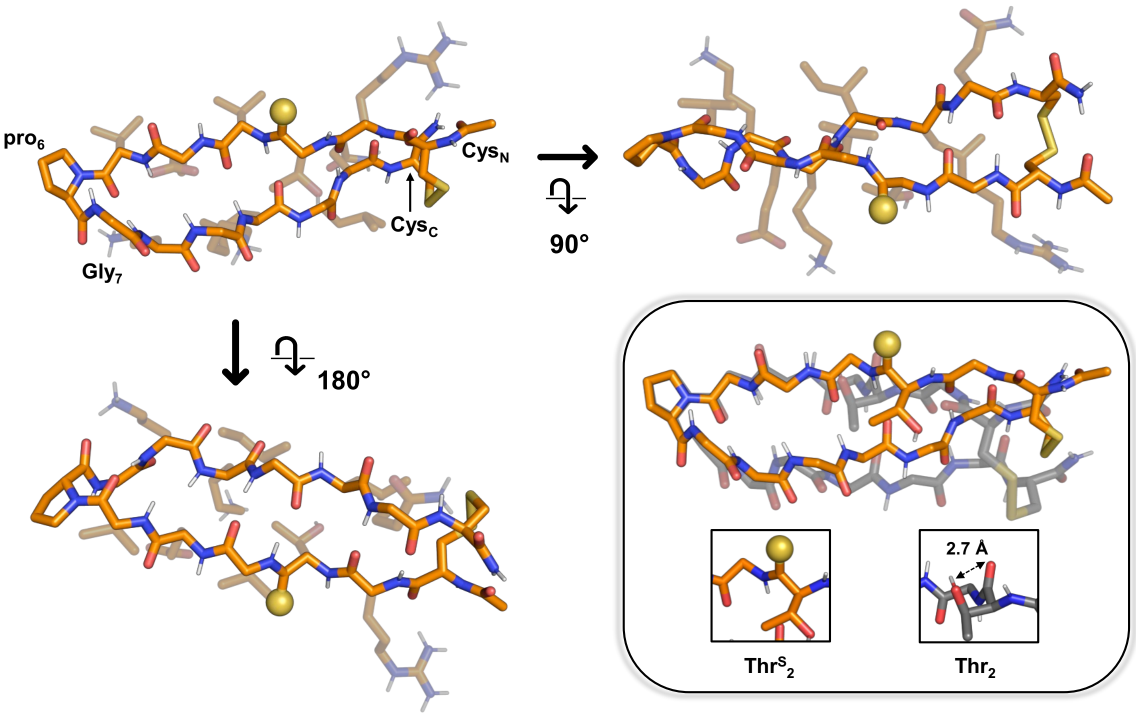

Figure S19. Modelled structure of HPT-Thr$^S{}_2$ Folded control peptide based on NOE derived distance constraints. In the box, the HPT-Thr$^S{}_2$ Folded control is overlaid with the HPT Folded control. An atomic detail that is different with thioamide incorporation includes flipping of the Thr side-chain. In HPT, the Thr carbonyl is within hydrogen bonding distance of the side-chain hydroxyl group. But in HPT-Thr$^S{}_2$, the side-chain is flipped away either because of the thioamide sterics or because it is a weaker hydrogen bond acceptor. The thioamide of HPT-Thr$^S{}_2$ engages as a hydrogen bond donor with the same strength as the oxoamide in HPT (distance between the Thr_2 NH and the Ile_{10} carbonyl: 2.2 Å for the thioamide and 2.3 Å for the oxoamide).

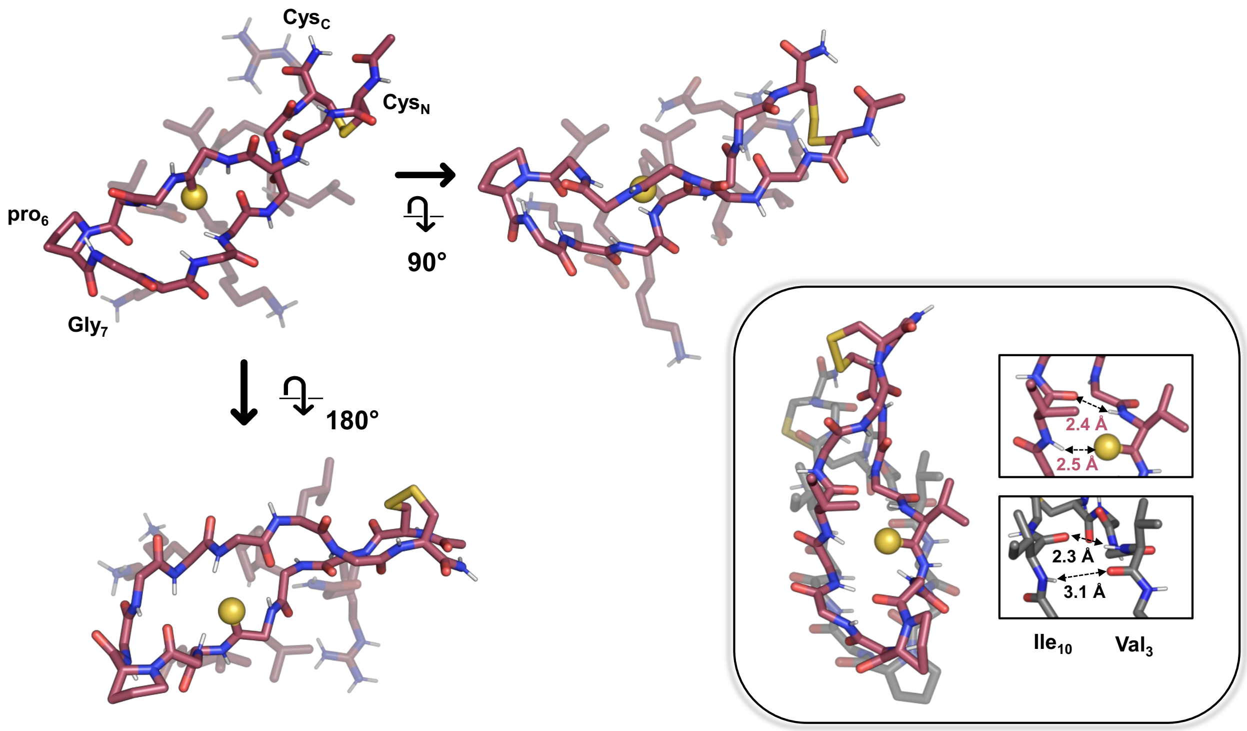

Figure S20. Modelled structure of HPT-Val$^S_3$ Folded control peptide based on NOE derived distance constraints. In the box, the HPT-Val$^S_3$ Folded control is aligned with the HPT Folded control around the thioamide. Despite the dramatic twist of this β-hairpin that presumably allows for accommodation of the internal thioamide, the thioamide has similar hydrogen bonding distances to HPT. This is unexpected but might result because Val$_3$ is conformationally restricted being closer to pro$_6$. This agrees with theoretical studies that suggest reduced conformational flexibility of the residue proceeding the thioamide.[15, 16]

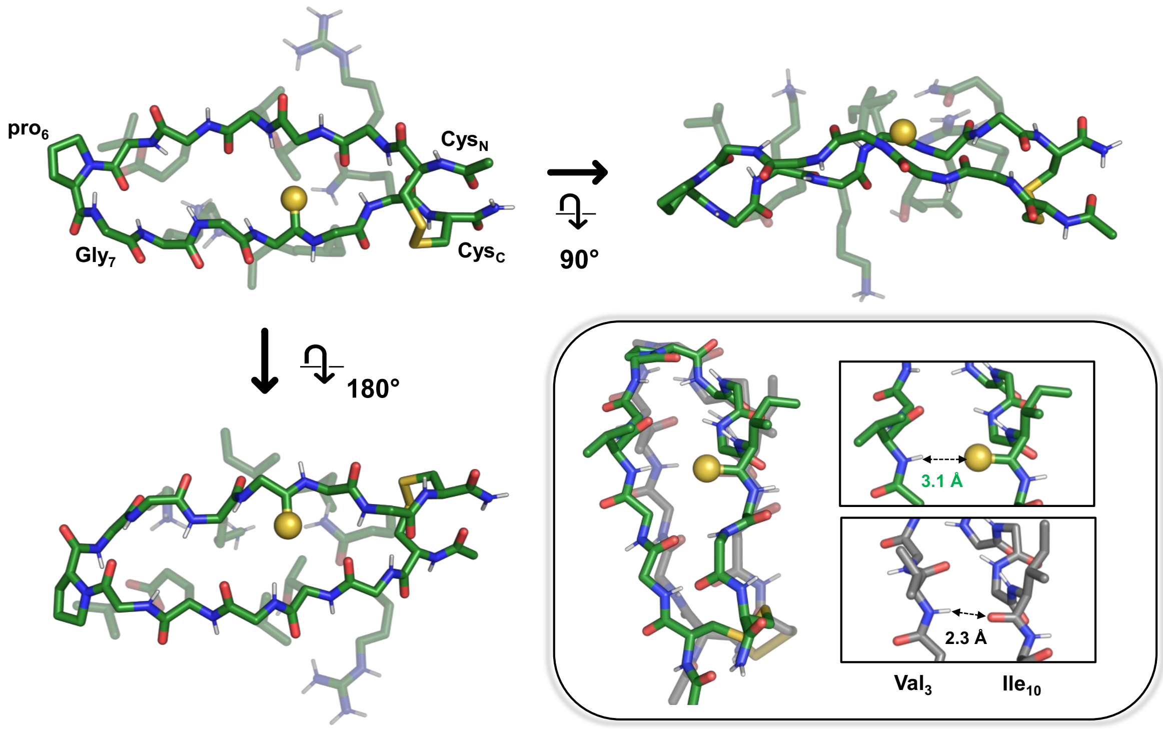

Figure S21. Modelled structure of HPT-Ile$^{S}{}_{10}$ Folded control peptide based on NOE derived distance constraints. In the box, the HPT-Ile$^{S}{}_{10}$ Folded control is overlaid with the HPT Folded control. Although the structures align well (backbone RMSD of 0.63 Å), there is an increase in the distance between the thiocarbonyl of Ile$^{S}{}_{10}$ and the NH of Val$_3$. This corresponds with the thioamide being a weaker hydrogen bond acceptor.

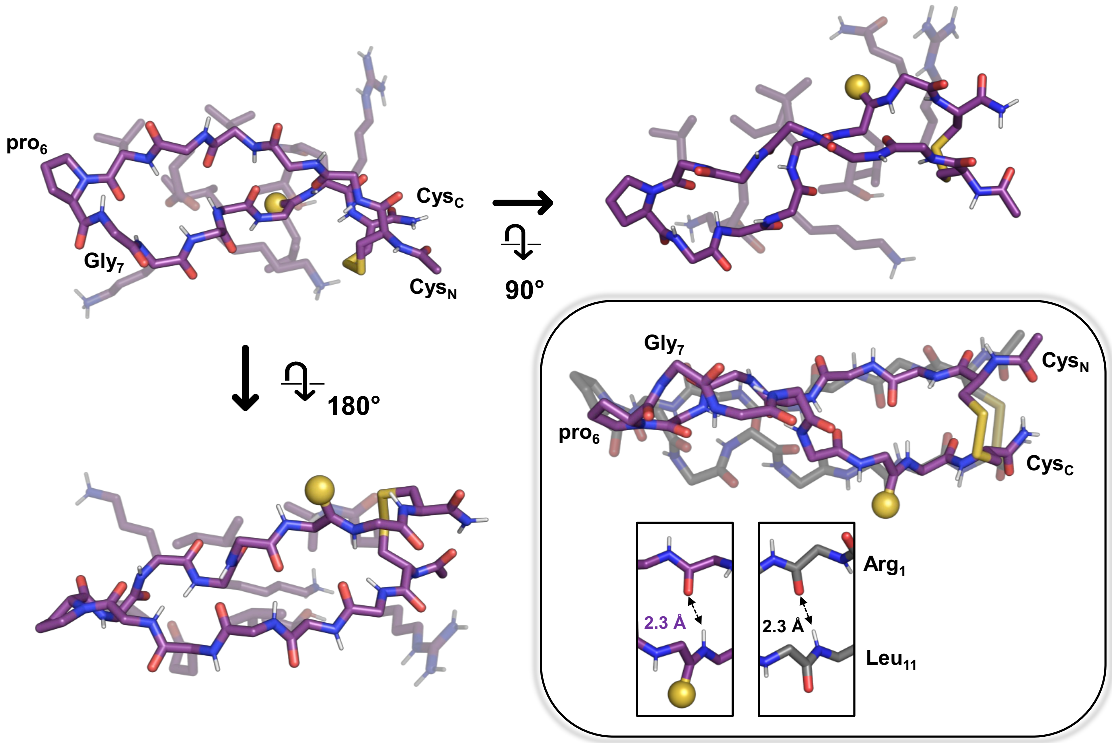

Figure S22. Modelled structure of HPT-Leu$^{S}_{11}$ Folded control peptide based on NOE derived distance constraints. In the box, the HPT-Leu$^{S}_{11}$ is aligned with the Folded control around the thioamide. The thioamide engages as a hydrogen bond donor with apparently the same strength as the oxoamide in HPT.

Structural Ensembles of the Thioamide-Containing Folded Control β-Hairpins

Ensemble Generation with Backrub Protocol. Ensembles of the β-hairpin structures were simulated using a backrub protocol starting with the constrained relax structures. Backrub is an application in Rosetta which makes local rotations about an axis dictated by chosen backbone atoms.[17] Additionally, side-chain optimization is performed using the side chain mover according to the Dunbrack distributions. The entire β-hairpin structures were subjected to backrub 100 independent times using the beta_nov16_cart score function with constraints set to a weight of 1. A constraint weight of 1 was found to optimally model conformational flexibility without great deviation from the experimental data (**Table S11).** The ten lowest energy structures from backrub were retained and analyzed by full atom and Cα RMSD (**Table S11).** PyMOL session files are available on our GitHub at https://github.com/ejp-lab/EJPLab_Computational_Projects/tree/master/BetaHairpin/BackrubStructures.

Table S11. Deviation, energy score and RMSD values for the backrub generated Folded control ensembles

	**Average Deviation from NOE Derived Distances for All Structures**	**Average Score of All Structures (REU)**	**Average Cα RMSD for 10 Lowest Energy Structures (Å)**	**Average Full Atom RMSD for 10 Lowest Energy Structures (Å)**
HPT-Thr$^{S}_{2}$	0.17 ± 0.13	498.6 ± 96.9	0.61 ± 0.12	1.77 ± 0.14
HPT-Val$^{S}_{3}$	0.22 ± 0.18	1241.3 ± 106.5	0.54 ± 0.16	1.77 ± 0.32
HPT-Ile$^{S}_{10}$	0.28 ± 0.21	1191.0 ± 311.1	1.33 ± 0.47	2.37 ± 0.54
HPT-Leu$^{S}_{11}$	0.28 ± 0.22	917.6 ± 252.1	0.84 ± 0.13	1.91 ± 0.22

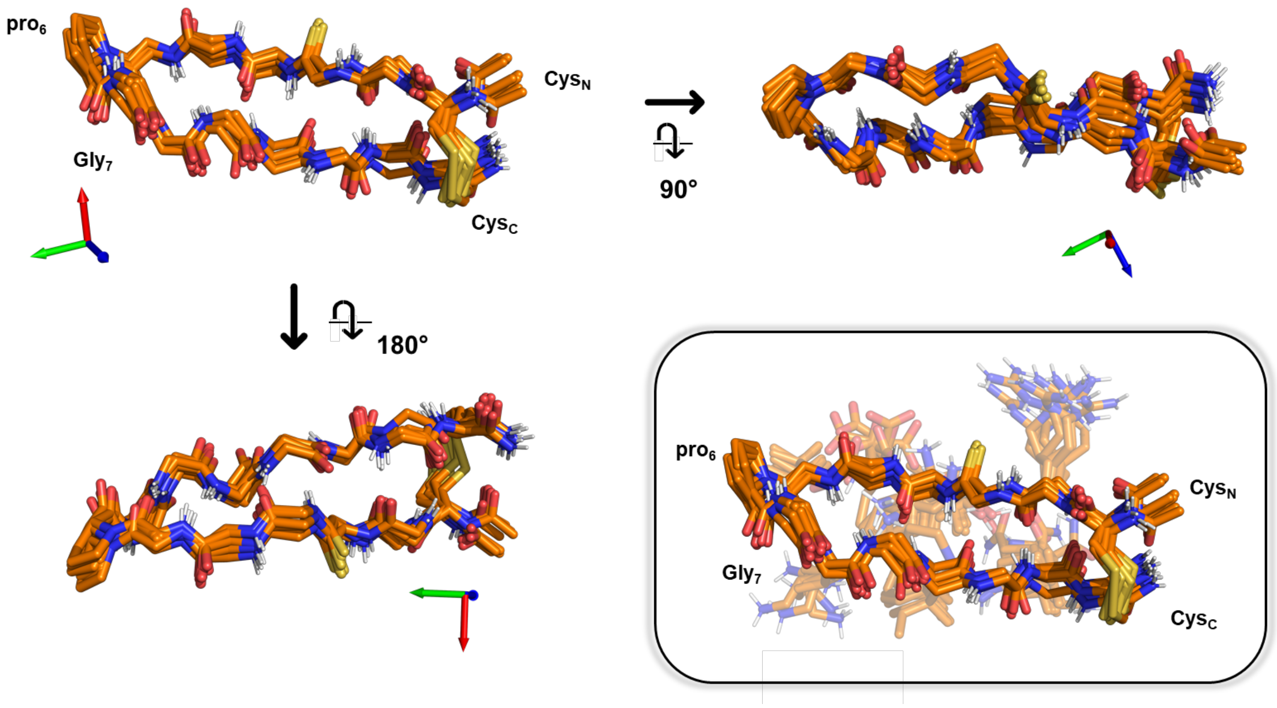

Figure S23. Ensemble generation of the HPT-Thr$^{S}_{2}$ Folded control peptide with the backrub protocol. Shown are the 10 lowest energy structures aligned**.** In the box, the initial orientation of the ensemble displayed, but with the side-chains shown.

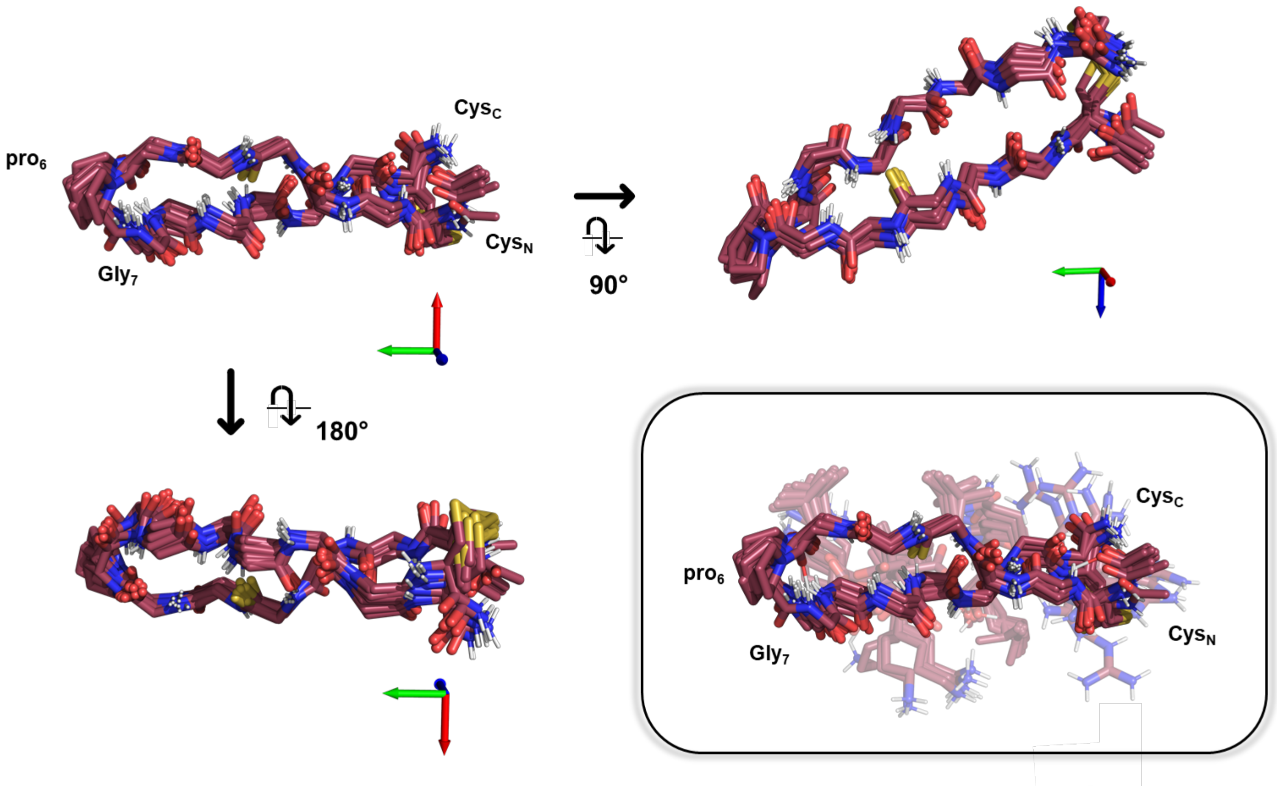

Figure S24. Ensemble generation of the HPT-Val$^{S}{}_{3}$ Folded control peptide with the backrub protocol. Shown are the 10 lowest energy structures aligned. In the box, the initial orientation of the ensemble displayed, but with the side-chains shown.

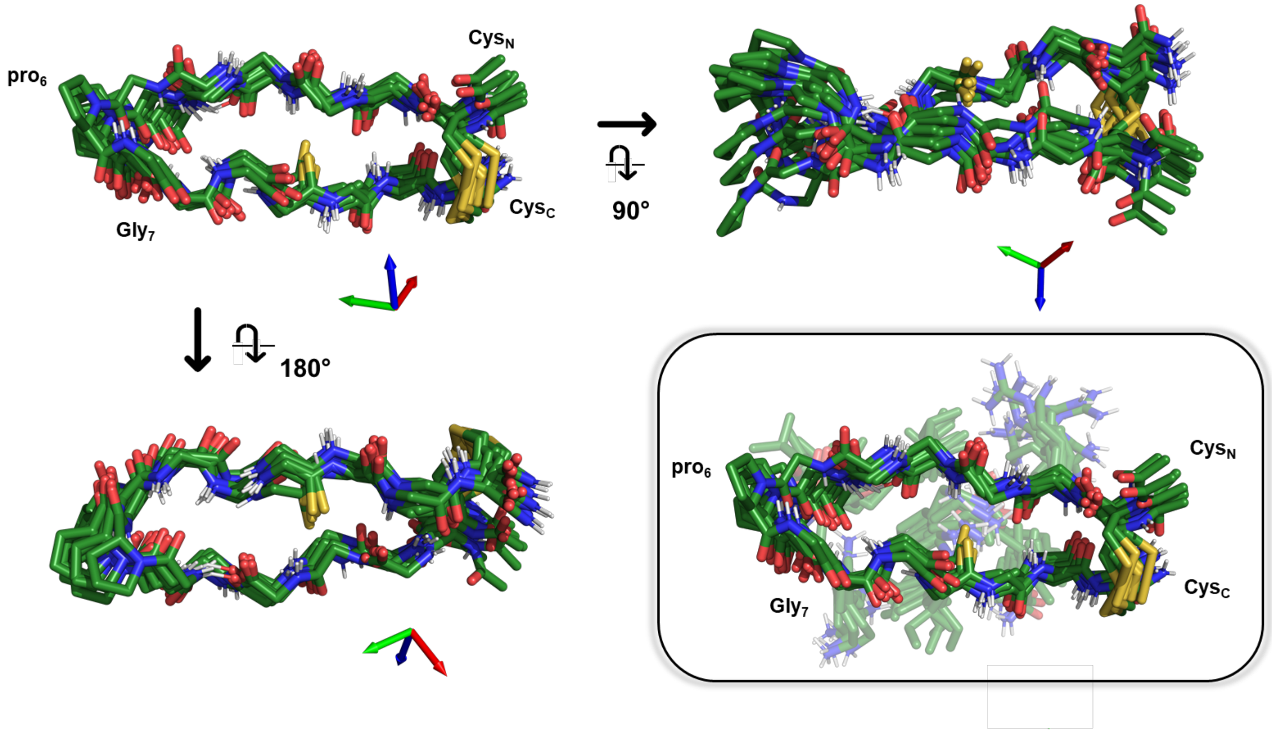

Figure S25. Ensemble generation of the HPT-Ile$^{S}_{10}$ Folded control peptide with the backrub protocol. Shown are the 10 lowest energy structures aligned. In the box, the initial orientation of the ensemble displayed, but with the side-chains shown. The Cα and full atom RMSD for the 10 lowest energy structures are considerably higher for this peptide. This flexibility is particularly prevalent in the β-turn and the β-strand opposite the thioamide.

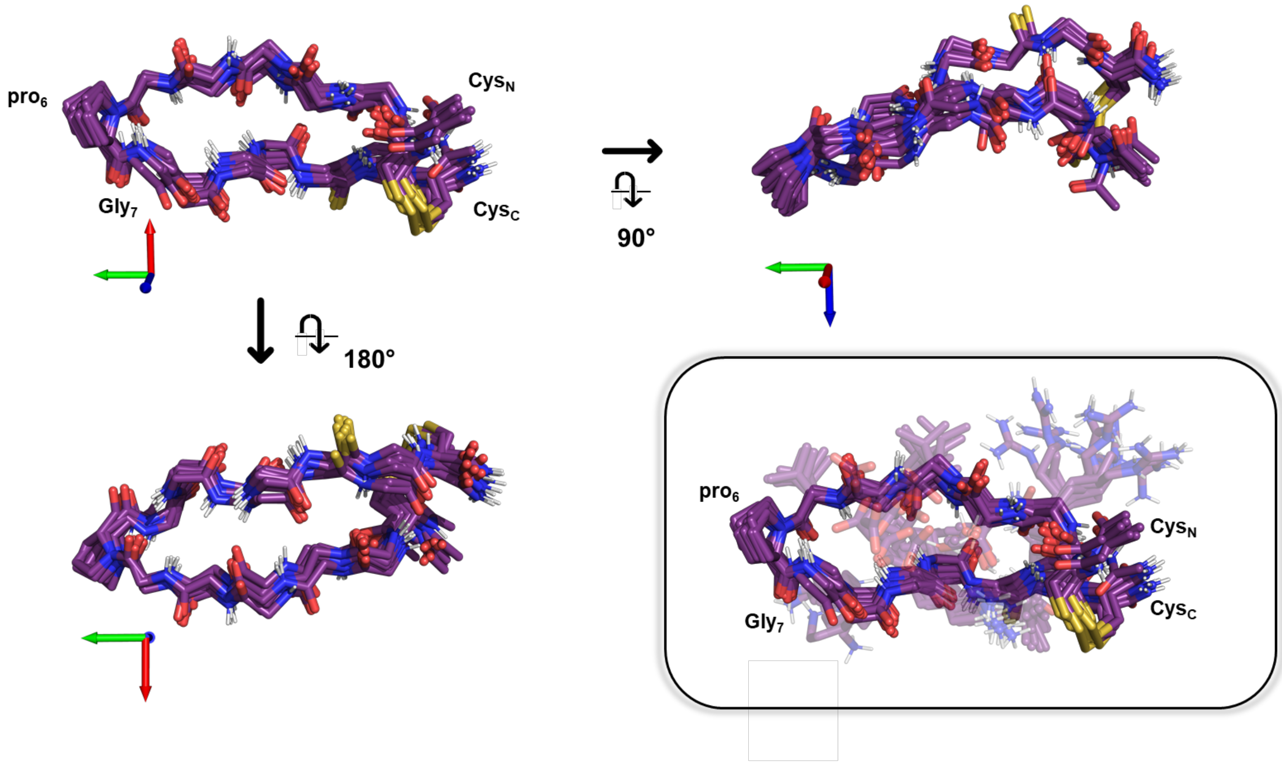

Figure S26. Ensemble generation of the HPT-Leu$^{S}_{11}$ Folded control peptide with the backrub protocol. Shown are the 10 lowest energy structures aligned. In the box, the initial orientation of the ensemble displayed, but with the side-chains shown.

Previous β-Hairpin Modification Studies

The following supplemental discussion provides a more detailed analysis of the effects of amino acid derivatives shown in Fig. 7. This discussion is generally limited to β-hairpins that are variations of YKL/HPT, in some cases replacing the proGly β-turn with AsnGly. Unless explicitly stated, all stability comparisons are made using $\Delta\Delta G_{Folding}$ with the parent peptide as a reference.

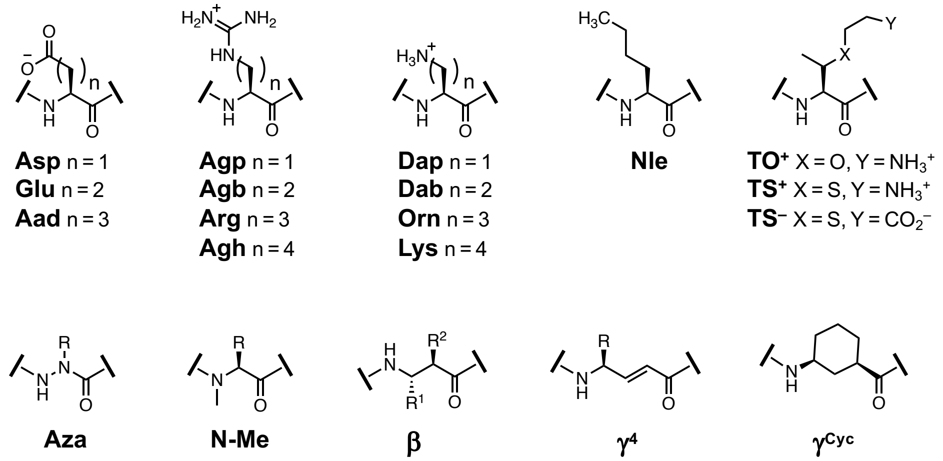

Figure S27. Structure of amino acid derivatives.

Steric Interactions. β-branched amino acids promote β-sheet propensity; however, the hydrophobicity of the γ-methyl groups of Val or Thr can cause solubility problems. Therefore, the ability of β-branched derivatives of charged amino acids to increase foldedness of a β-hairpin was explored by Gellman. TS^{-}_{4} replacement of Glu_4 and TS^{+}_{9} replacement of Lys_9 in the YKL scaffold stabilized the β-hairpin by -0.5 and -0.6 kcal/mol, respectively.[18] Notably, TO^{+} was slightly destabilizing (+0.1 kcal/mol), showing that the subtle change of going from a thioether to an ether can have a significant effect on hairpin stability.

Gellman and Muir found that adding β-branched residues to the ends of the YKL scaffold also increased stability up to a length of seven amino acids in each strand.[19] The addition of two Thr to each terminus stabilized the peptide by -0.3 kcal/mol, but further residues (with the exception of an all-Thr extension) did not increase stability, suggestive of an intrinsic limit to the length of isolated β-sheets.

π-Interactions. Waters and co-workers have used the YKL system to study a variety of π-system interactions of aromatic amino acids as part of their studies on the structural impacts of post-translational modifications. In a 2003 study of a 14-residue derivative of the YKL scaffold, the impact of salt bridges versus aromatic/aromatic interactions on stability was analyzed.[20] Switching from a lateral cross-strand Phe-Phe interaction to a Glu-Lys salt bridge at various positions resulted in a loss of stability (~0.3 kcal/mol). Their subsequent studies investigated interactions with a single aromatic side-chain.

The cation-π interaction was investigated with an AsnGly derivative of the YKL scaffold with Trp at position 2.[21] Methylation of Lys results in greater dispersion of the positive charge that should increase the strength of the cation-π interaction. Each methylation of Lys_9 (Lys-Me, Lys-Me2, and Lys-Me3) increased the stability of the hairpin by -0.2 kcal/mol. Acetylation of Lys decreased the positive charge of the side-chain, but surprisingly did not result in a less-folded β-hairpin.[22] Acetylation changed the geometry of the interaction, where the amide NH is aimed at the face of the Trp indole ring.

Replacement of Lys_9 with Arg in a AsnGly derivative of the YKL scaffold with Trp_2 increased the stability by -0.3 kcal/mol.[23] This is presumably a result of the stronger interaction of the delocalized π system of the guanidinium

group, which can sample face-to-face and edge-to-face interactions.[24] Consistent with the importance of the face-to-face binding mode, methylation of Arg increased the stability by -0.6 kcal/mol.[25] The methylated Arg derivatives exhibit the same stability as incorporation of Lys-Me3.

Sulfur-arene interactions have also been shown to be significant stabilizing forces in biological systems.[26] In an AsnGly derivative of the YKL scaffold, a stabilizing diagonal interaction of -0.3 to -0.5 kcal/mol between Phe_2 or Trp_2 with Met_9 was observed (in comparison to Lys_9).[27] Investigations using norleucine (Nle) at position 9 allowed Waters to identify a significant role for the methylene adjacent to the cationic Lys nitrogen and for the hydrophobic component of the Met-π interaction.[28] Nle_9 interacted with Trp_2 or Phe_2 in a nonspecific manner, whereas the Lys_9 interaction is specific and tighter.

In contrast to the cation-π and sulfur-arene interactions, Waters' investigation on the effect of phosphorylation cross-strand from an aromatic amino acid did not show evidence of a favourable anion-π interaction.[29, 30] Phosphoserine, phosphothreonine and phosphotyrosine were incorporated opposite Trp_2 in a AsnGly derivative of the YKL scaffold. This was generally destabilizing by ~ 1 kcal/mol. C-terminal phosphoserine incorporation was less destabilizing (0.4-0.5 kcal/mol).

Ion-pairing. Cheng has performed a highly systematic analysis of ion-pairing interactions in β-hairpins using the HPT scaffold. They studied cross-strand lateral ion-pairing by changing the side-chain length and charged groups at positions 4 and 9. The greatest stability was achieved when the side-chain lengths of the negatively charged Glu/Asp derivative (position 4) and the positivity charged Lys derivative (position 9) were matched.[1] Variations in stability with side-chain length result from a complex interplay between alterations in charge density with distance from the backbone, increased entropic penalties for longer side-chains, and steric effects of side-chain methylene groups. Similar trends were observed with positively charged Arg derivatives at position 9, with a preference for longer side-chains due to the more conformationally restricted guanidinium group.[31] A follow-up study in which the positively and negatively charged amino acids were swapped revealed that the right-handed twist of the backbone played a role as well in favoring certain matched lengths.[32]

Backbone. Among backbone modifications, only aza-amino acids have been studied in the YKL/HPT scaffold, therefore we will include studies using other small hairpins for this section. Aza-amino acids were incorporated by Proulx into the YKL scaffold, aza-Val at Val_3 was highly disruptive to foldedness (1.26 kcal/mol) whereas aza-Gly was better accepted at this position (0.75 kcal/mol), but still less stable than YKL.[33] Aza-Val has a similar fraction folded to D-Val incorporation, therefore aza-amino acids were assumed to be disruptive to β-hairpin stability due to the adaptive chirality at Nα**.**

Substitution of two adjacent α-amino acids by a β-amino acid was performed by Horne in a β-hairpin sequence derived from a C-terminal segment of GB1. The peptides could fold into hairpin structures, but are destabilized by 0.5-0.6 kcal/mol per αα-substitution.[34] Incorporation of linear (E)-vinylogous γ^4-residues in the same scaffold were also moderately destabilizing (0.5 kcal/mol), whereas a cyclically constrained γ-residue was stabilizing (-0.3 to -0.6 kcal/mol).[35, 36] Although N-methylated analogues were tested at non-hydrogen bonded positions, the correct isomers were still less stable than the other backbone modifications explored by Horne. This was especially prevalent for the positions near the turn where the N-methyl energetically restricts that backbone, disfavouring anti-parallel β-sheet structure.

References

1. H.-T. Kuo, C.-J. Fang, H.-Y. Tsai, M.-F. Yang, H.-C. Chang, S.-L. Liu, L.-H. Kuo, W.-R. Wang, P.-A. Yang, S.-J. Huang, S.-L. Huang and R. P. Cheng, *Biochemistry*, 2013, **52**, 9212-9222.
2. S. Batjargal, Y. Huang, Y. J. Wang and E. J. Petersson, *J. Pept. Sci.*, 2014, **20**, 87-91.
3. Y. Huang, J. J. Ferrie, X. Chen, Y. Zhang, D. M. Szantai-Kis, D. M. Chenoweth and E. J. Petersson, *Chem. Commun.*, 2016, **52**, 7798-7801.
4. C. R. Walters, D. M. Szantai-Kis, Y. Zhang, Z. E. Reinert, W. S. Horne, D. M. Chenoweth and E. J. Petersson, *Chem. Sci.*, 2017, **8**, 2868-2877.
5. M. M. Szantai-Kis, C. R. Walters, T. M. Barrett, E. M. Hoang and J. J. Petersson, *Synlett*, 2017, DOI: 10.1055/s-0036-1589027.
6. S. Mukherjee and J. Chatterjee, *Journal of Peptide Science*, 2016, **22**, 664-672.
7. B. Khatri, P. Bhat and J. Chatterjee, *Journal of Peptide Science*, 2020, **26**, e3248.
8. A. Bax and D. G. Davis, *J. Magn. Reson. (1969)*, 1985, **65**, 355-360.
9. A. A. Bothner-By, R. L. Stephens, J. Lee, C. D. Warren and R. W. Jeanloz, *J. Am. Chem. Soc.*, 1984, **106**, 811-813.
10. M. Piotto, V. Saudek and V. Sklenář, *J. Biomol. NMR*, 1992, **2**, 661-665.
11. S. Chaudhury, S. Lyskov and J. J. Gray, *Bioinformatics*, 2010, **26**, 689-691.
12. R. Mahalakshmi, S. Raghothama and P. Balaram, *J. Am. Chem. Soc.*, 2006, **128**, 1125-1138.
13. P. D. Thomas, V. J. Basus and T. L. James, *Proc. Natl. Acad. Sci. U. S. A.*, 1991, **88**, 1237.
14. S. Giannakoulias, S. R. Shringari, C. Liu, H. A. T. Phan, T. M. Barrett, J. J. Ferrie and E. J. Petersson, *J. Phys. Chem. B*, 2020, **124**, 8032-8041.
15. B. J. Lampkin and B. VanVeller, *J. Org. Chem.*, 2021, **86**, 18287-18291.
16. D. R. Artis and M. A. Lipton, *J. Am. Chem. Soc.*, 1998, **120**, 12200-12206.
17. C. A. Smith and T. Kortemme, *J. Mol. Biol.*, 2008, **380**, 742-756.
18. S. J. Maynard, A. M. Almeida, Y. Yoshimi and S. H. Gellman, *J. Am. Chem. Soc.*, 2014, **136**, 16683-16688.
19. H. E. Stanger, F. A. Syud, J. F. Espinosa, I. Giriat, T. Muir and S. H. Gellman, *Proc. Natl. Acad. Sci. U. S. A.*, 2001, **98**, 12015.
20. S. E. Kiehna and M. L. Waters, *Protein Sci.*, 2003, **12**, 2657-2667.
21. R. M. Hughes, M. L. Benshoff and M. L. Waters, *Chem. – Eur. J.*, 2007, **13**, 5753-5764.
22. R. M. Hughes and M. L. Waters, *J. Am. Chem. Soc.*, 2006, **128**, 13586-13591.
23. C. D. Tatko and M. L. Waters, *Protein Sci.*, 2003, **12**, 2443-2452.
24. J. C. Ma and D. A. Dougherty, *Chem. Rev.*, 1997, **97**, 1303-1324.
25. R. M. Hughes and M. L. Waters, *J. Am. Chem. Soc.*, 2006, **128**, 12735-12742.
26. L. M. Salonen, M. Ellermann and F. Diederich, *Angew. Chem., Int. Ed.*, 2011, **50**, 4808-4842.
27. C. D. Tatko and M. L. Waters, *Protein Sci.*, 2004, **13**, 2515-2522.
28. C. D. Tatko and M. L. Waters, *J. Am. Chem. Soc.*, 2004, **126**, 2028-2034.
29. A. J. Riemen and M. L. Waters, *Org. Biomol. Chem.*, 2010, **8**, 5411-5417.
30. A. J. Riemen and M. L. Waters, *J. Am. Chem. Soc.*, 2009, **131**, 14081-14087.
31. H.-T. Kuo, S.-L. Liu, W.-C. Chiu, C.-J. Fang, H.-C. Chang, W.-R. Wang, P.-A. Yang, J.-H. Li, S.-J. Huang, S.-L. Huang and R. P. Cheng, *Amino Acids*, 2015, **47**, 885-898.
32. C.-H. Huang, T. W. Wong, C.-H. Yu, J.-Y. Chang, S.-J. Huang, S.-L. Huang and R. P. Cheng, *Molecules*, 2021, **26**.
33. M. A. McMechen, E. L. Willis, P. C. Gourville and C. Proulx, *Molecules*, 2019, **24**.
34. G. A. Lengyel and W. S. Horne, *J. Am. Chem. Soc.*, 2012, **134**, 15906-15913.
35. G. A. Lengyel, Z. E. Reinert, B. D. Griffith and W. S. Horne, *Org. Biomol. Chem.*, 2014, **12**, 5375-5381.
36. G. A. Lengyel, G. A. Eddinger and W. S. Horne, *Org. Lett.*, 2013, **15**, 944-947.
